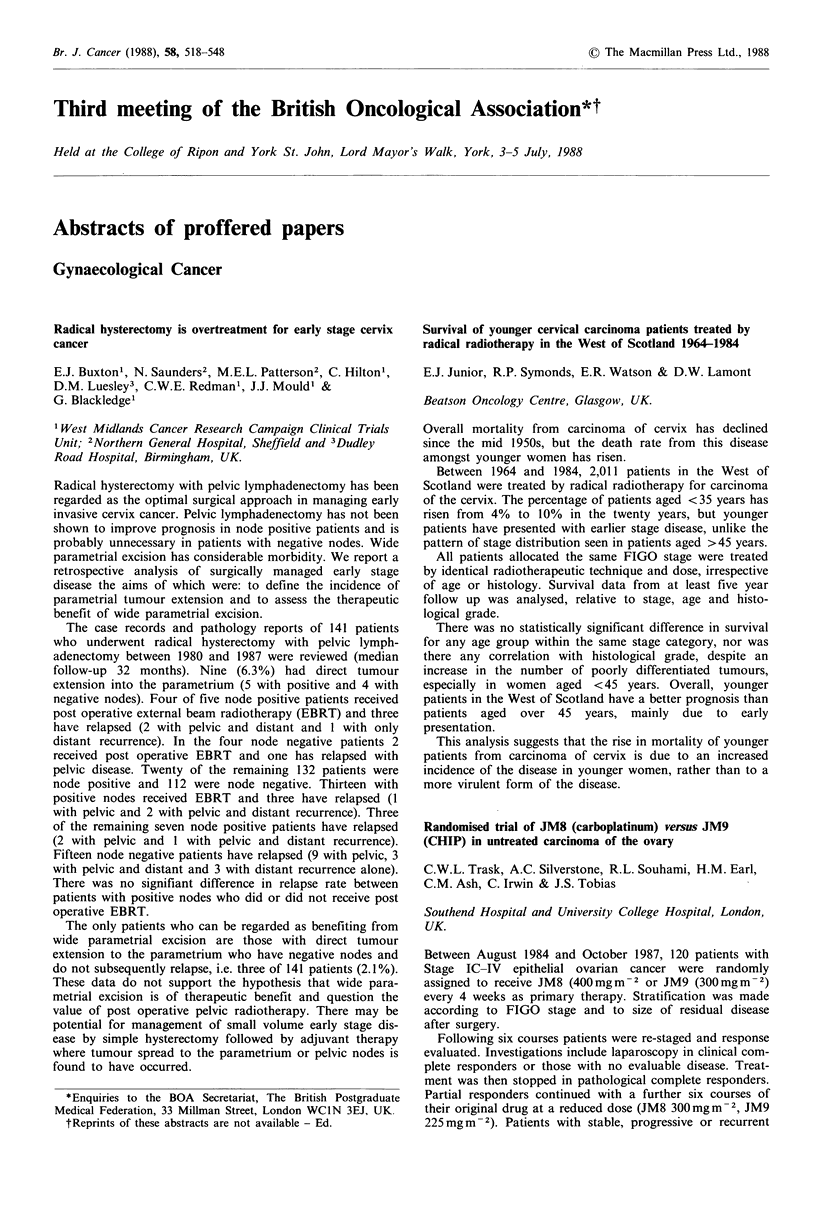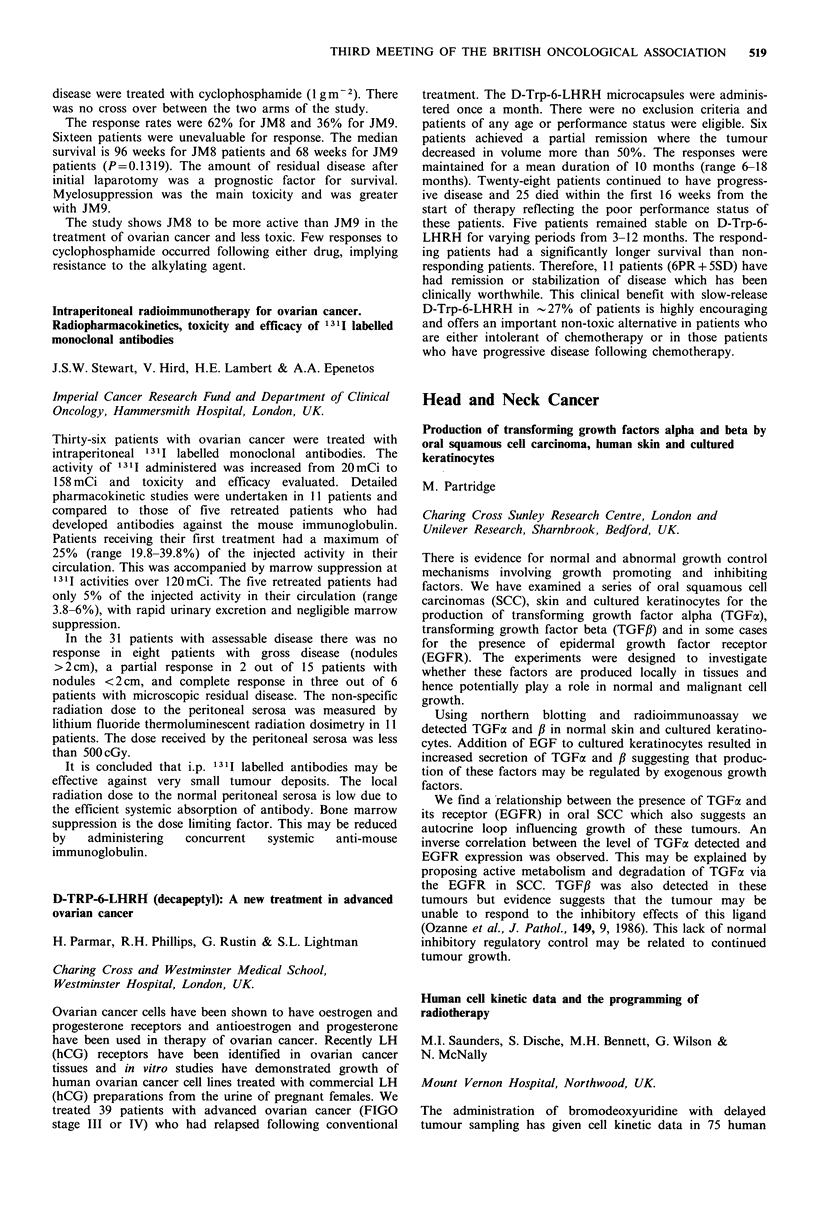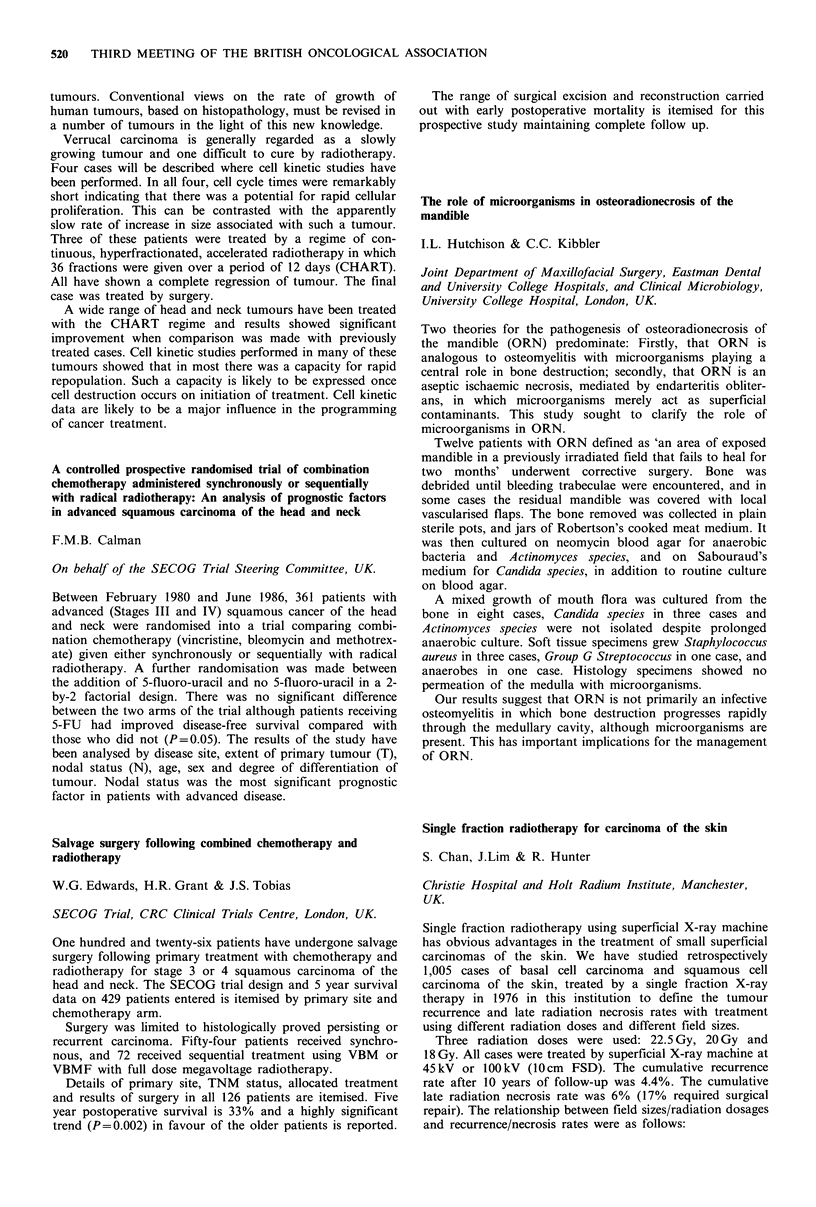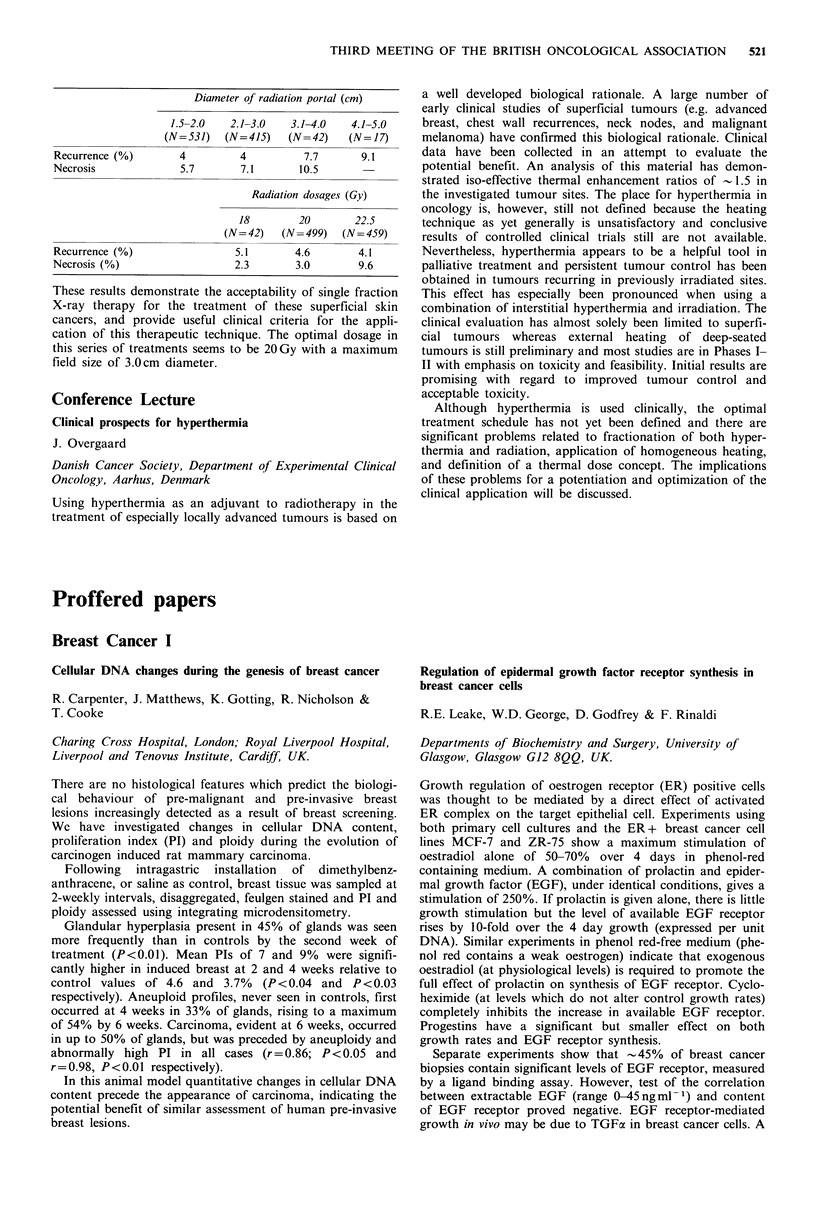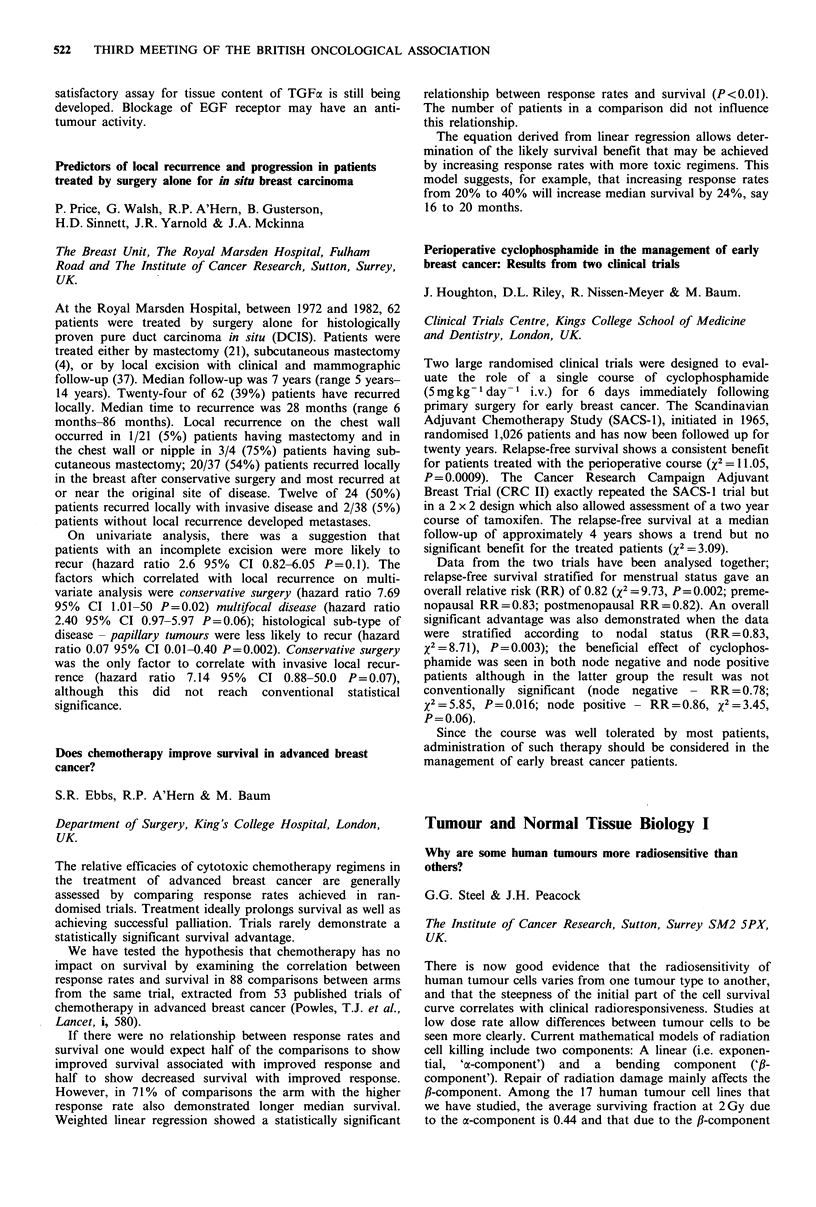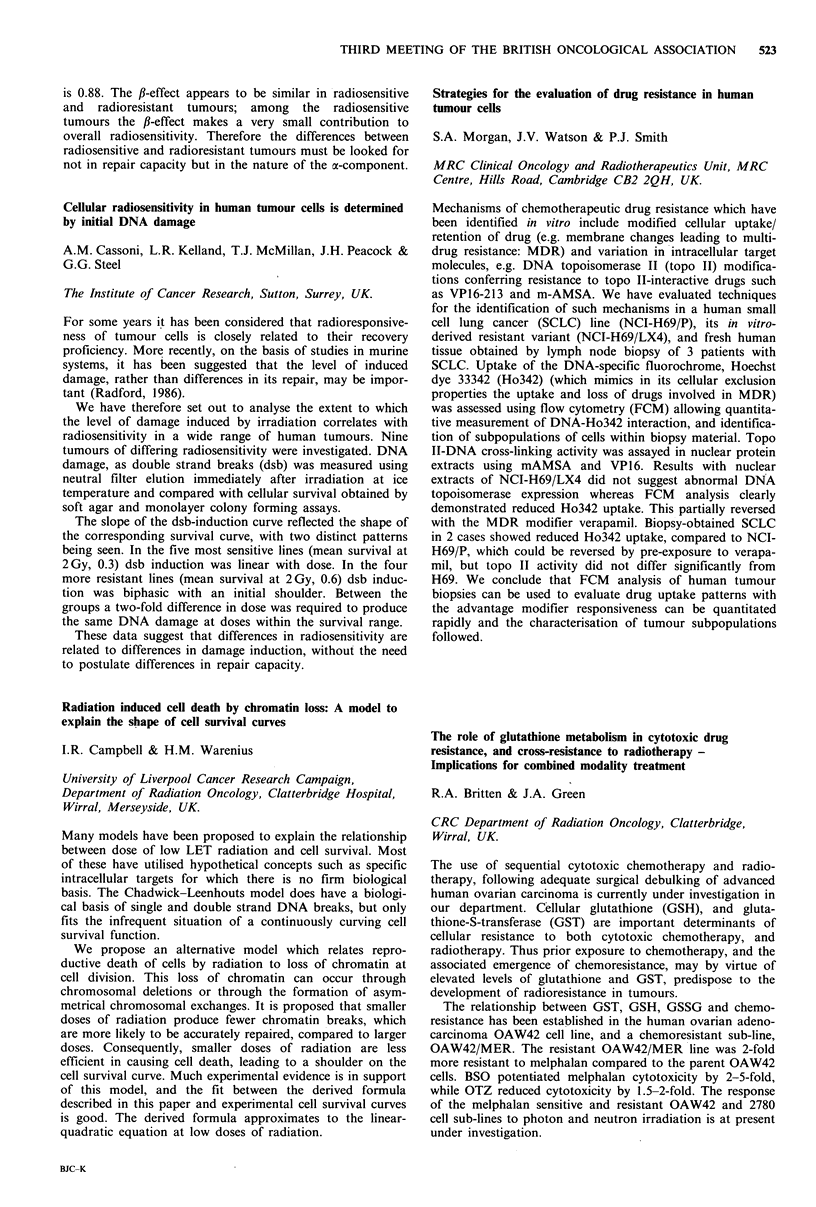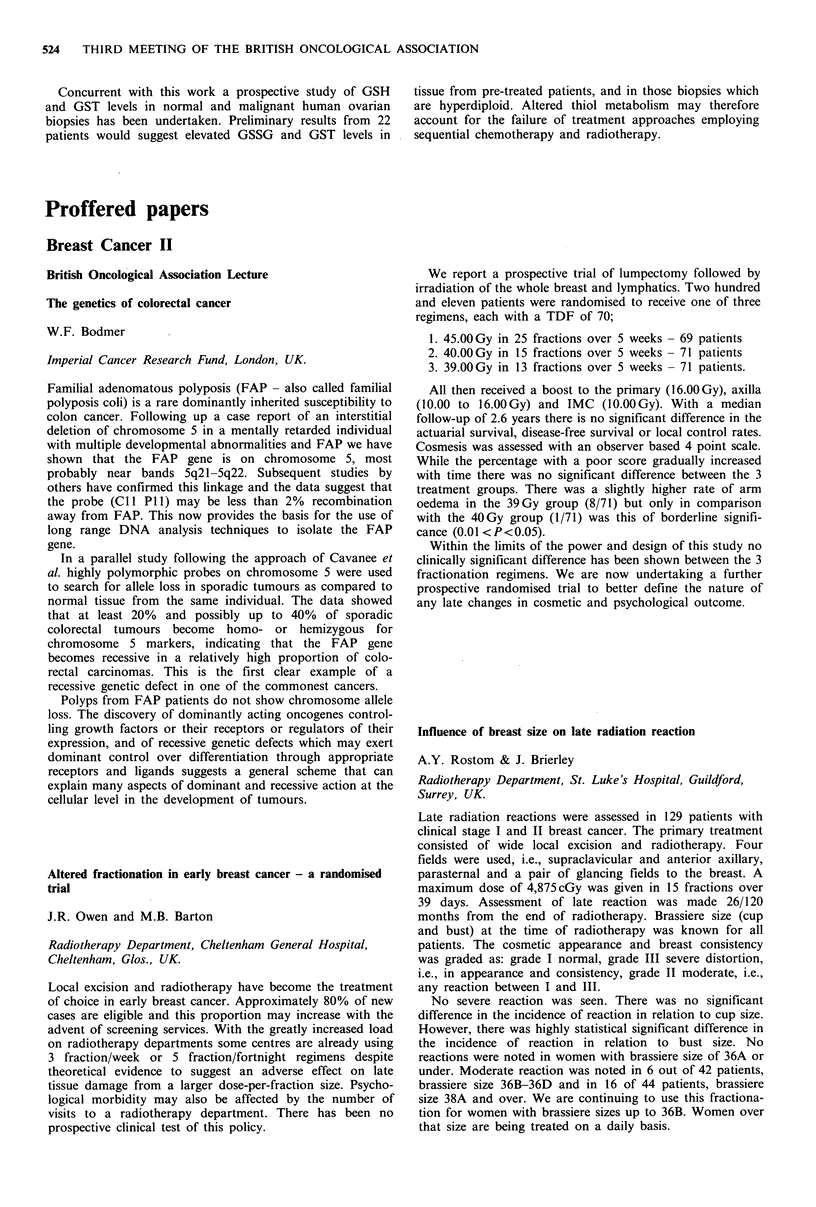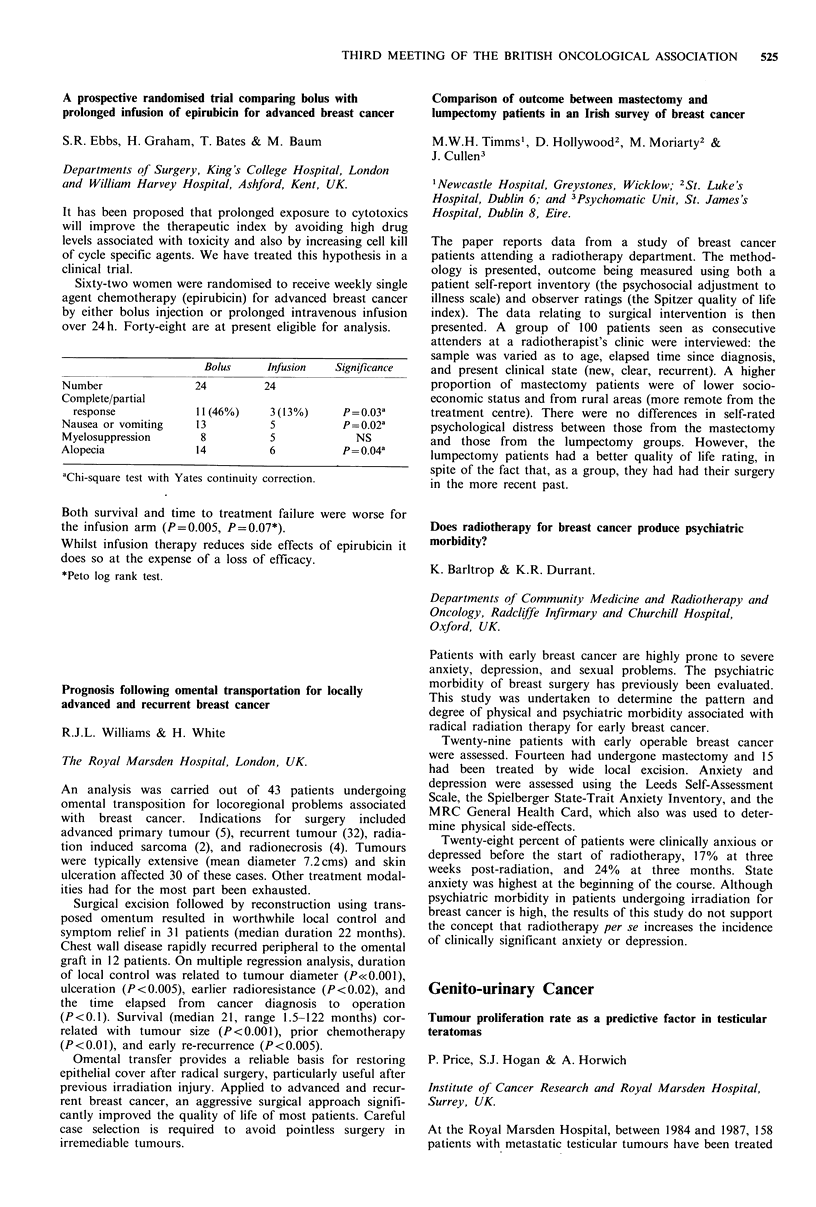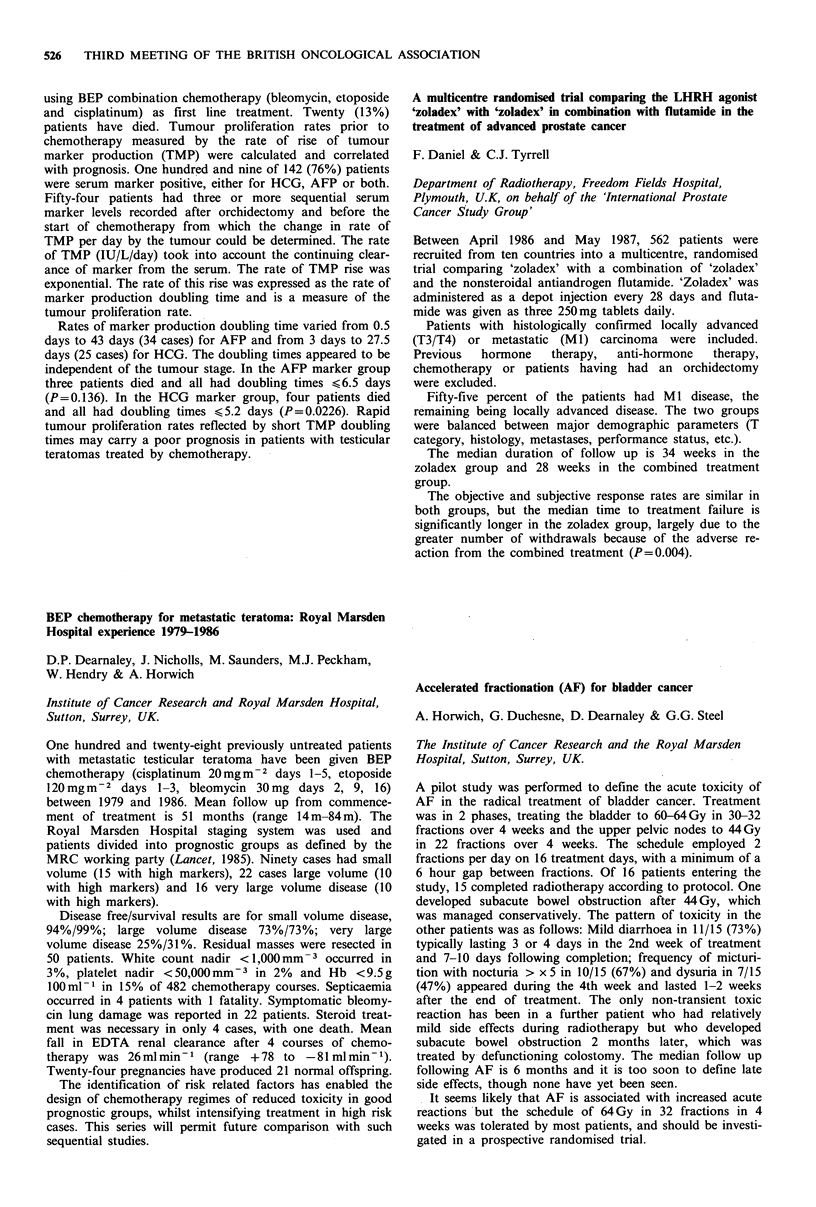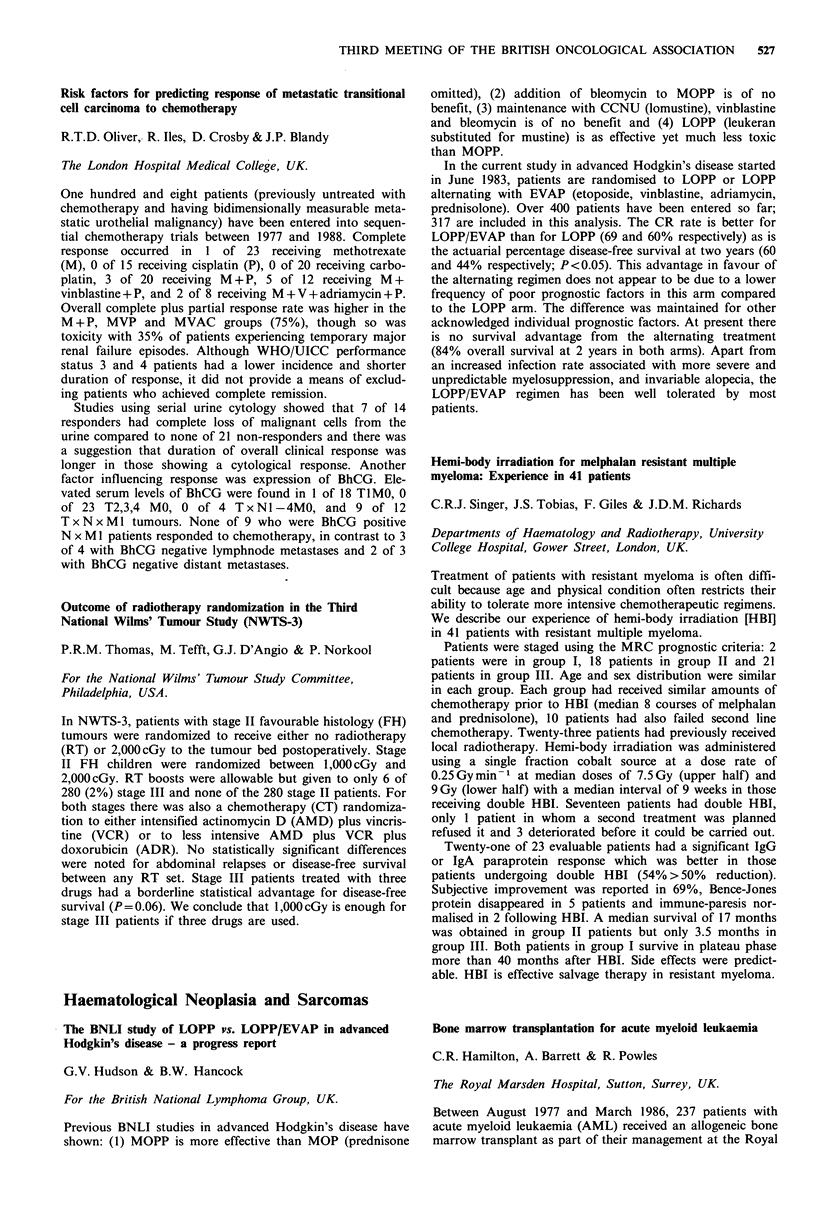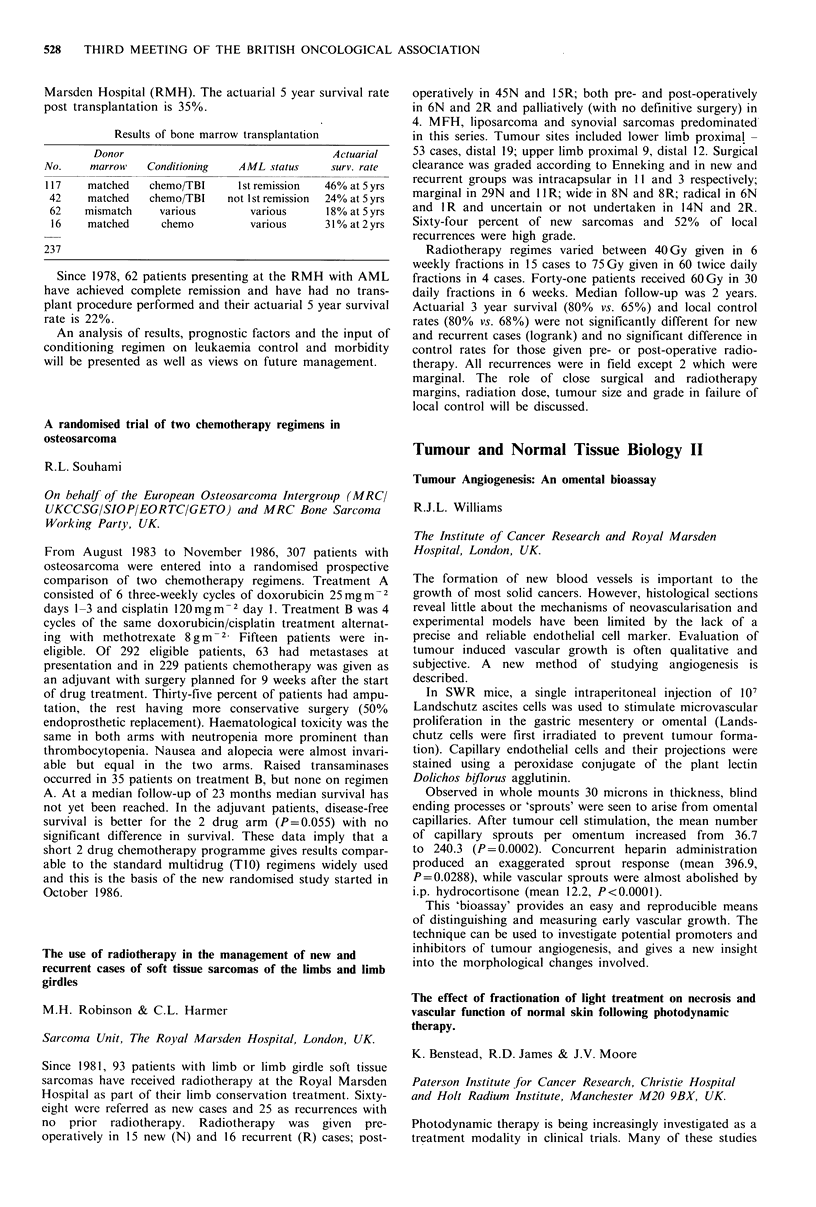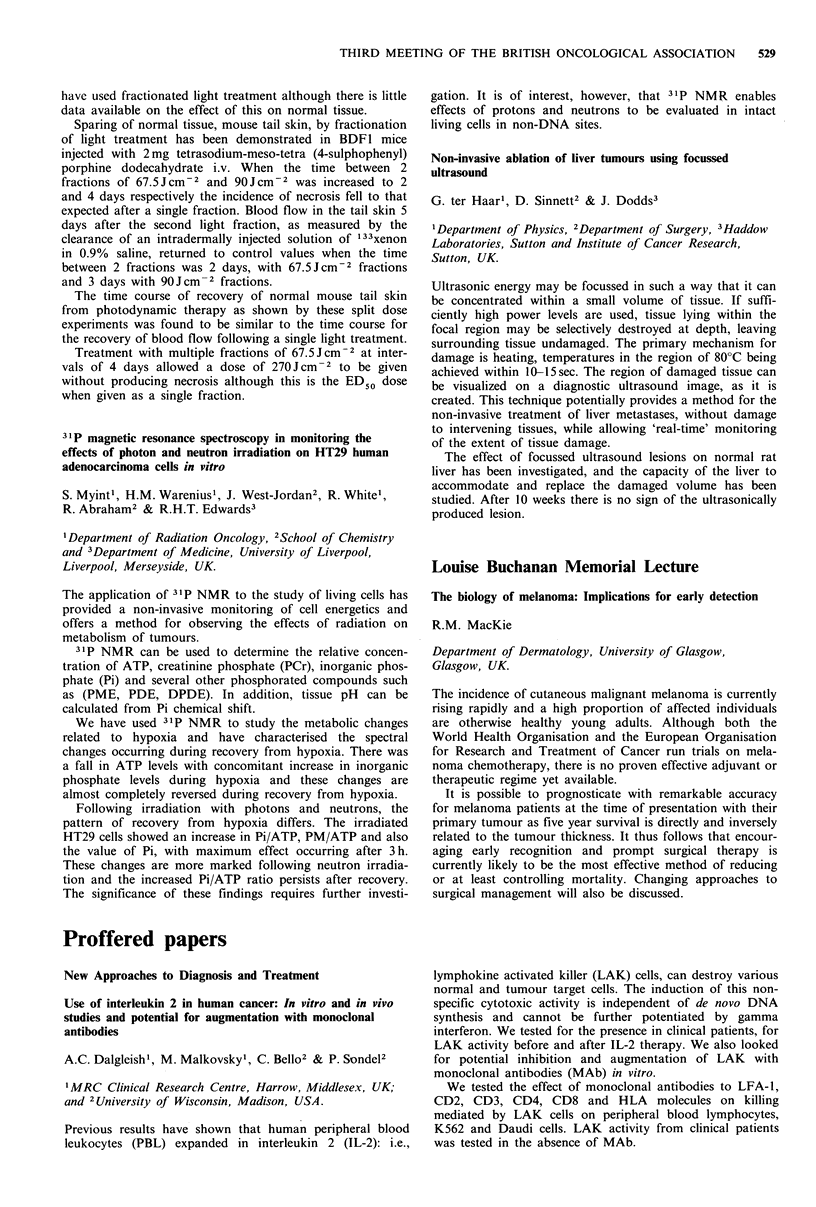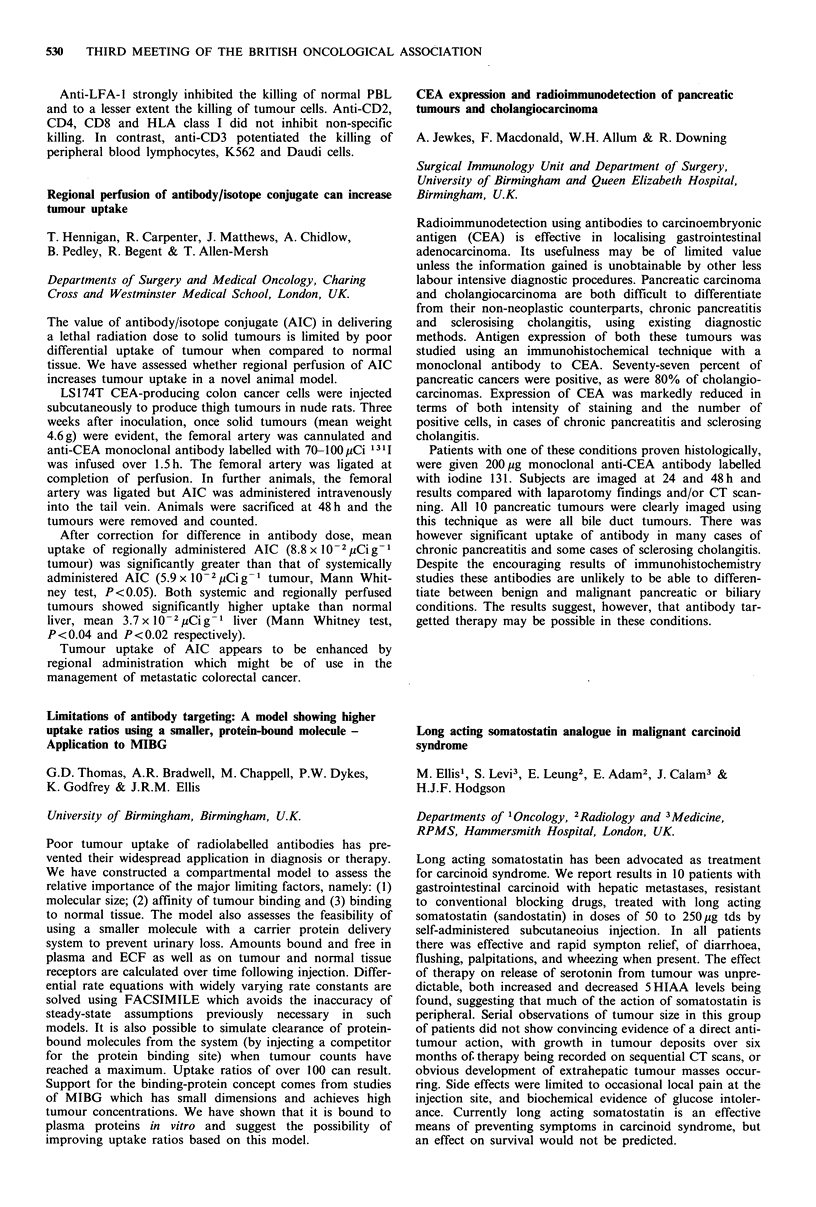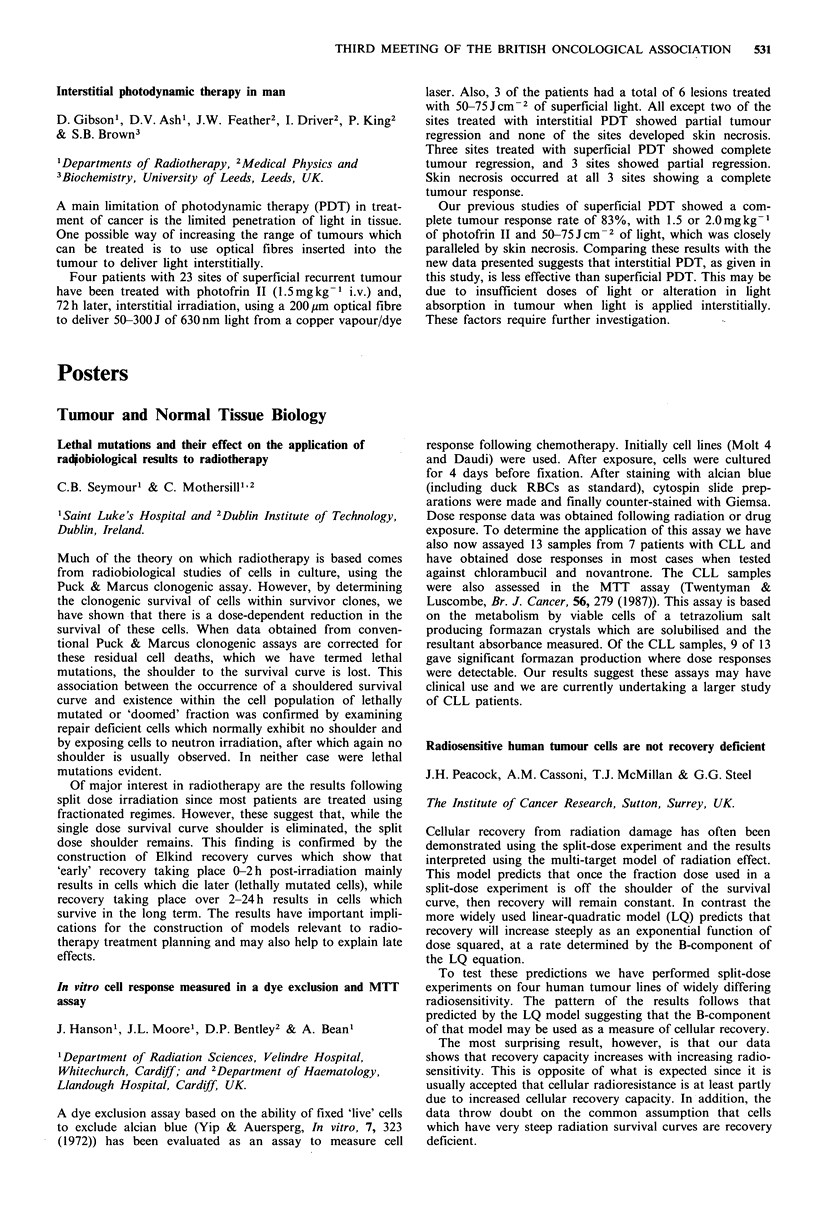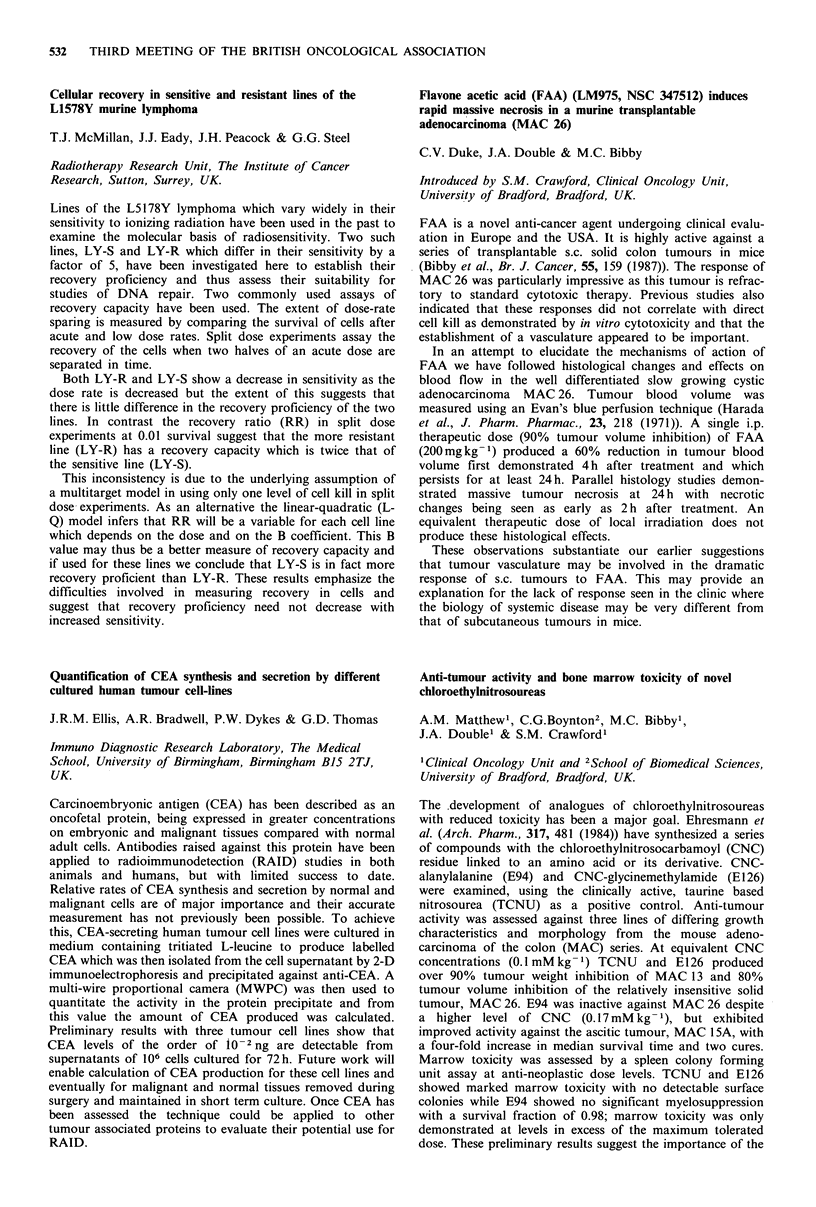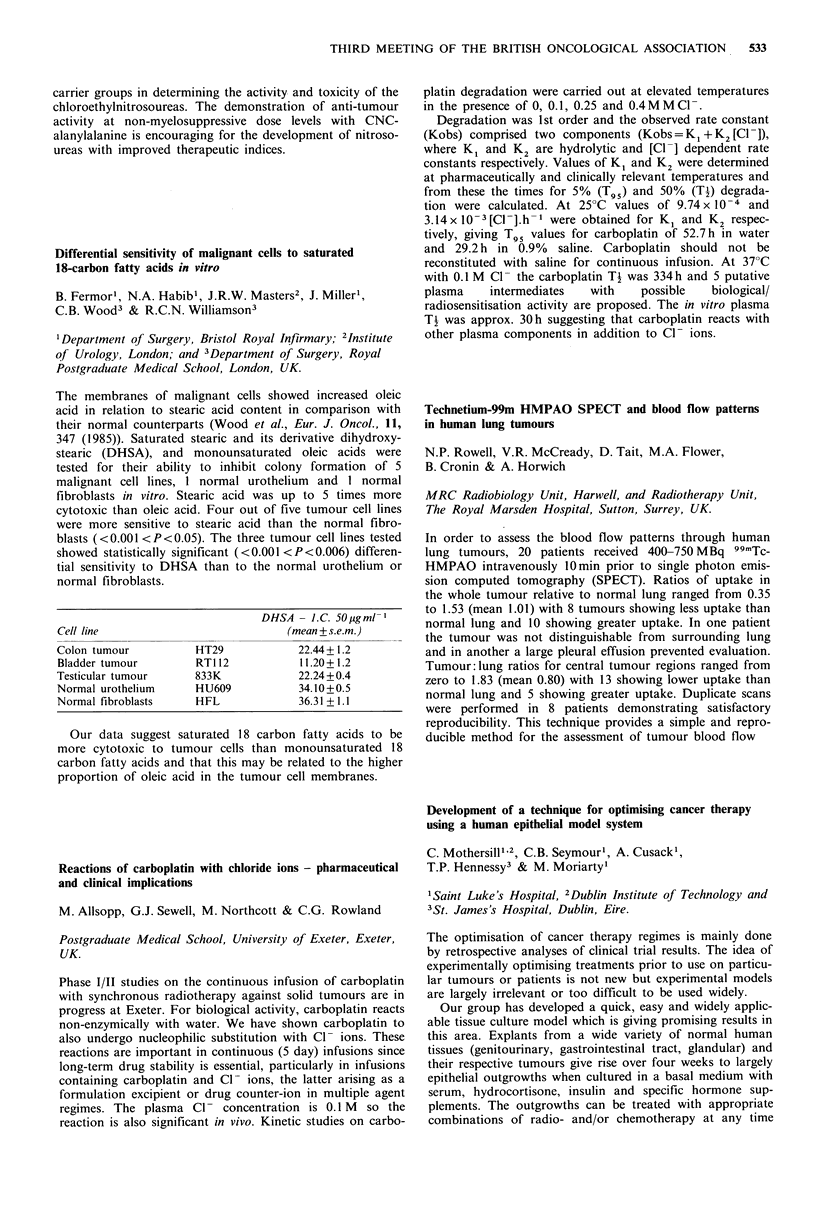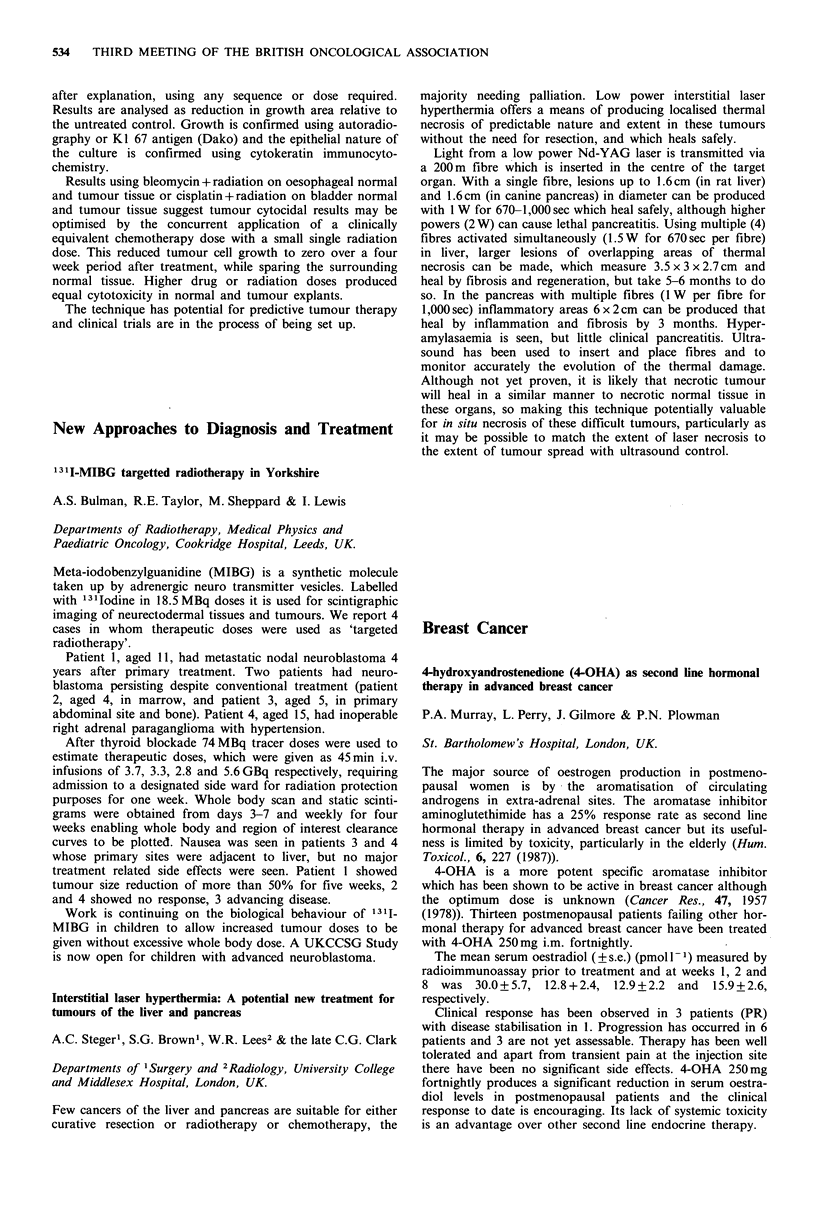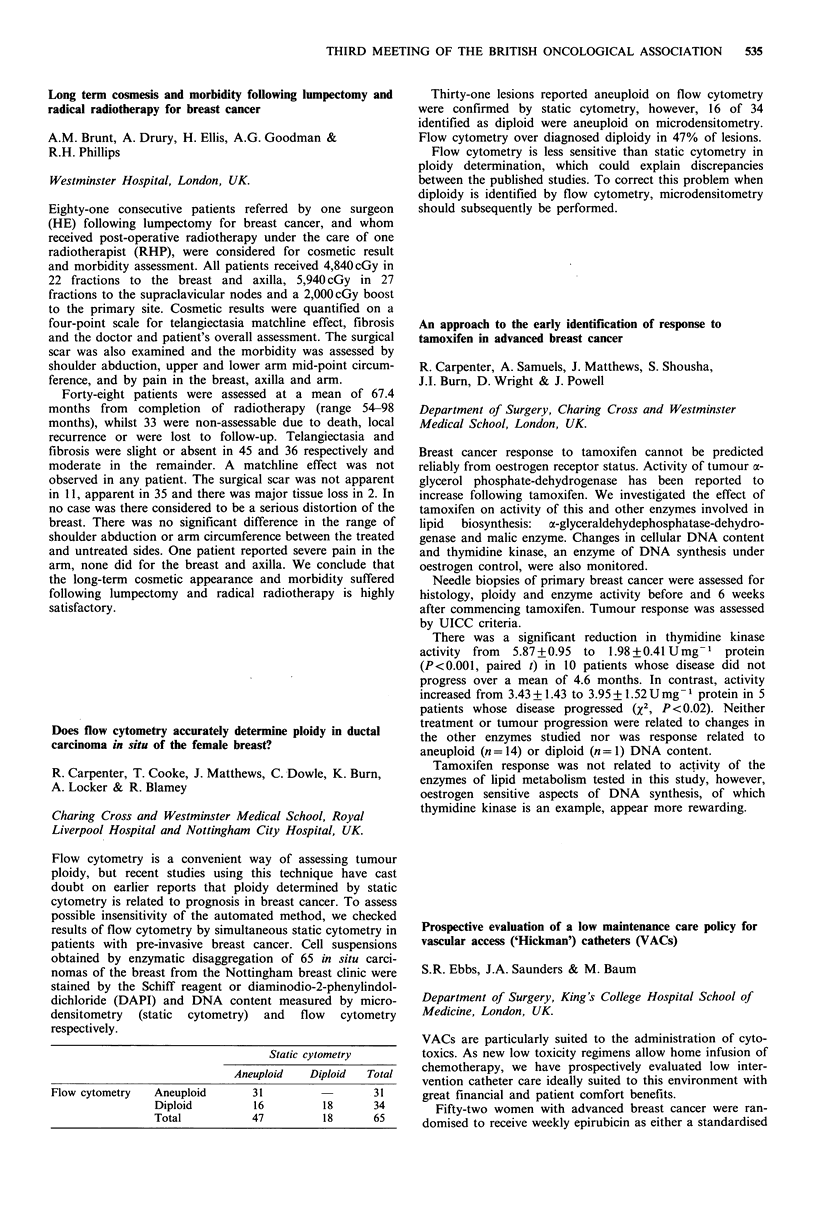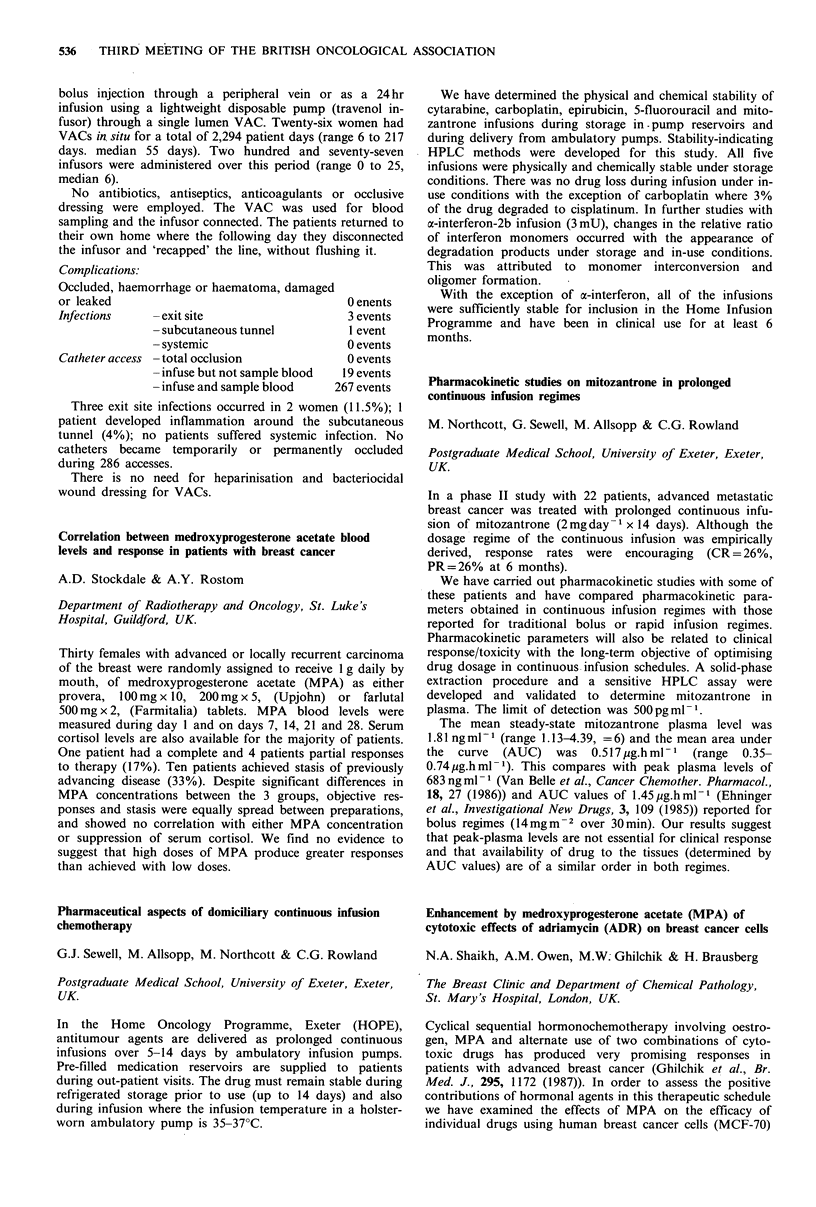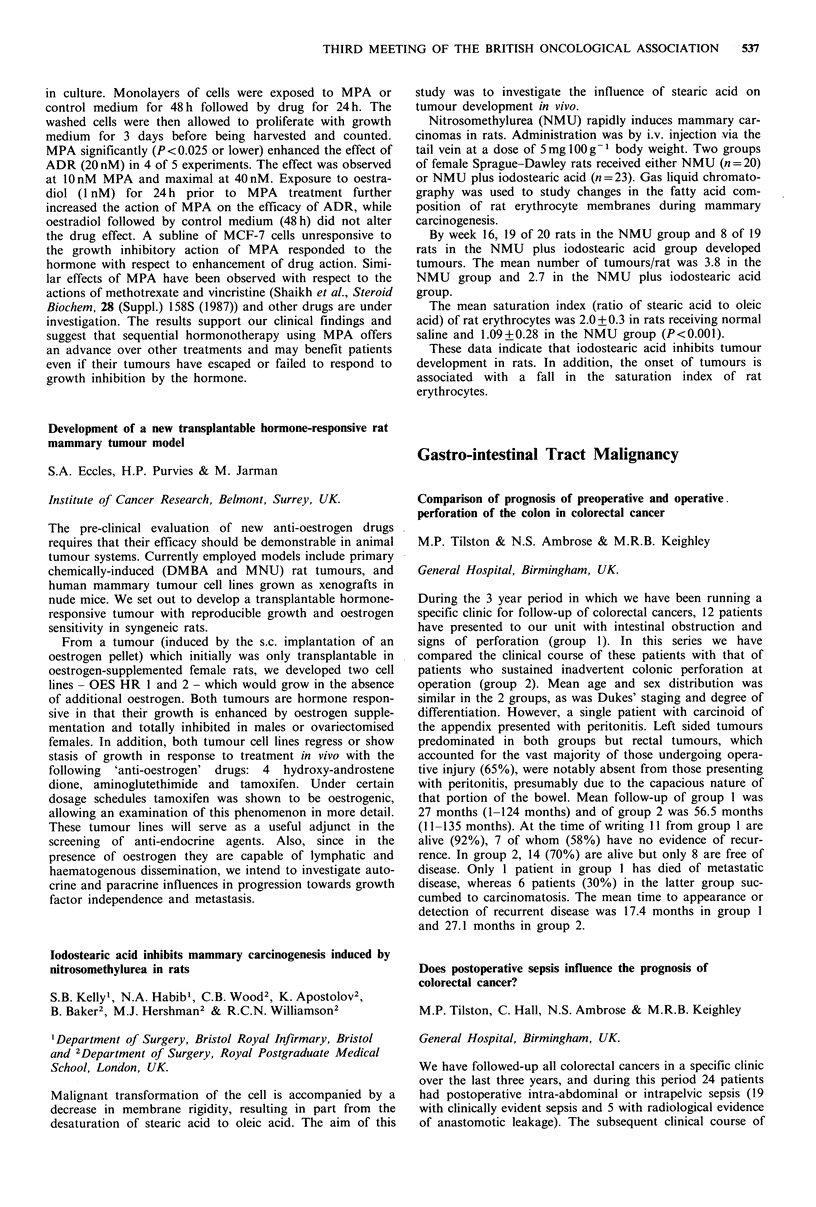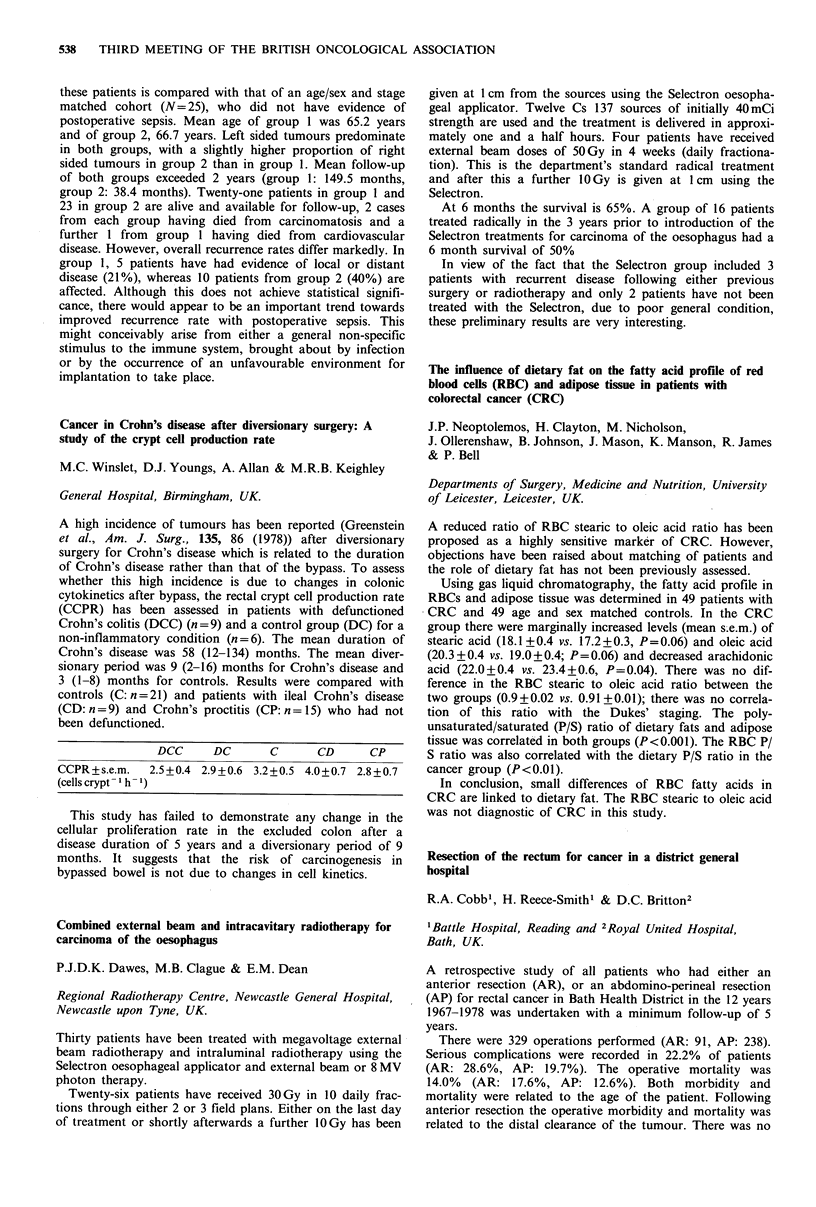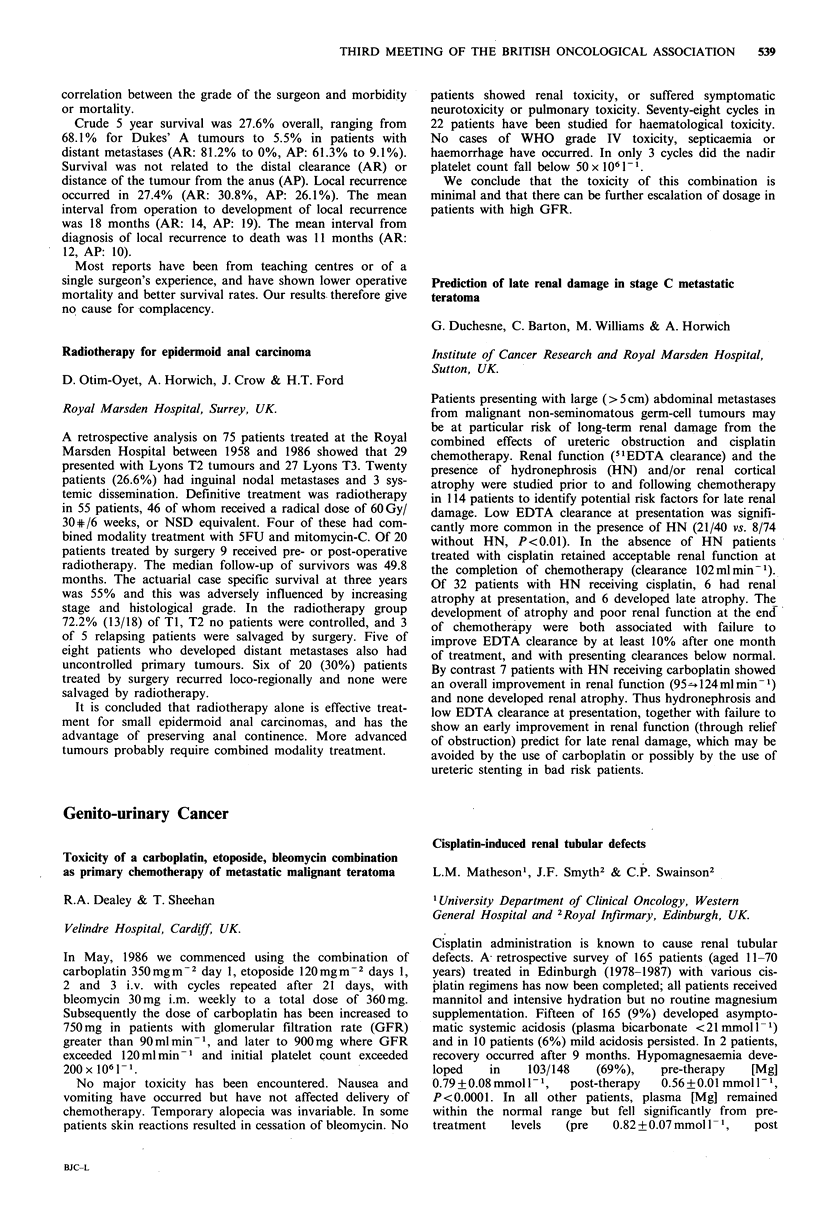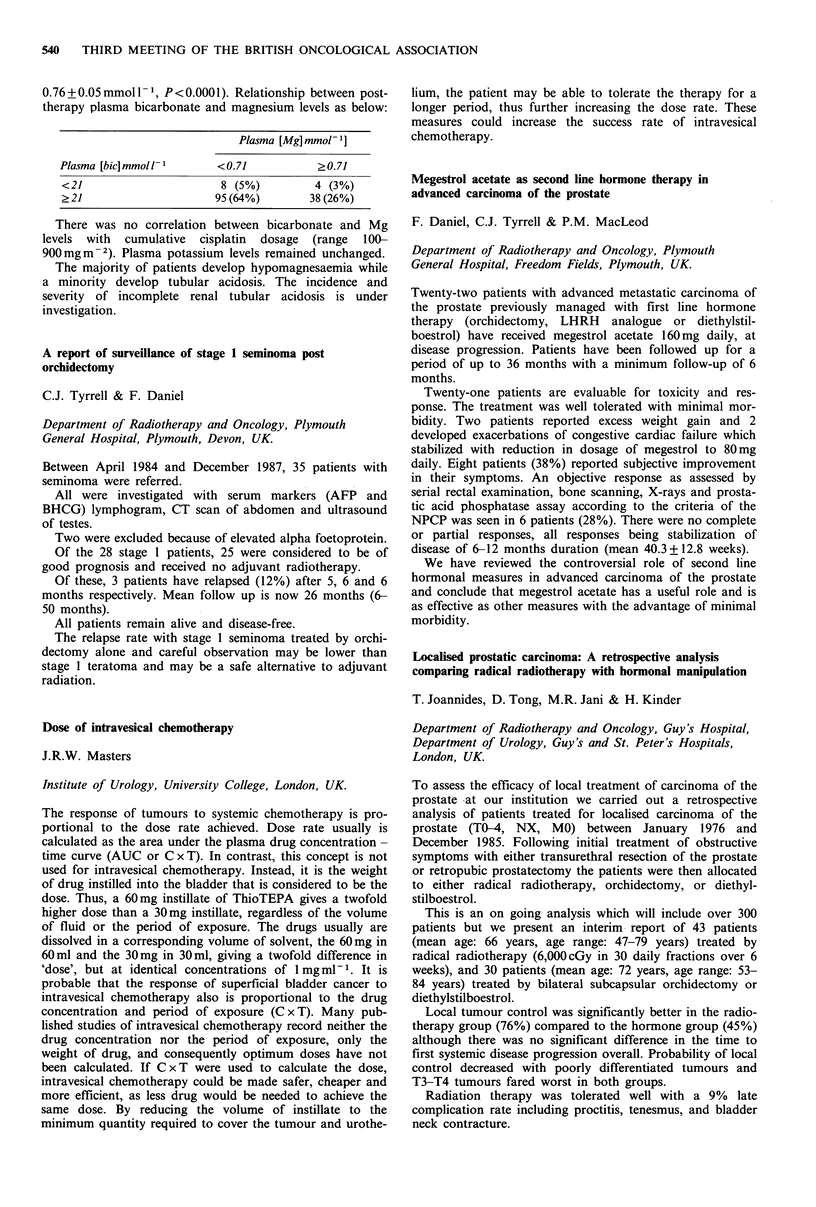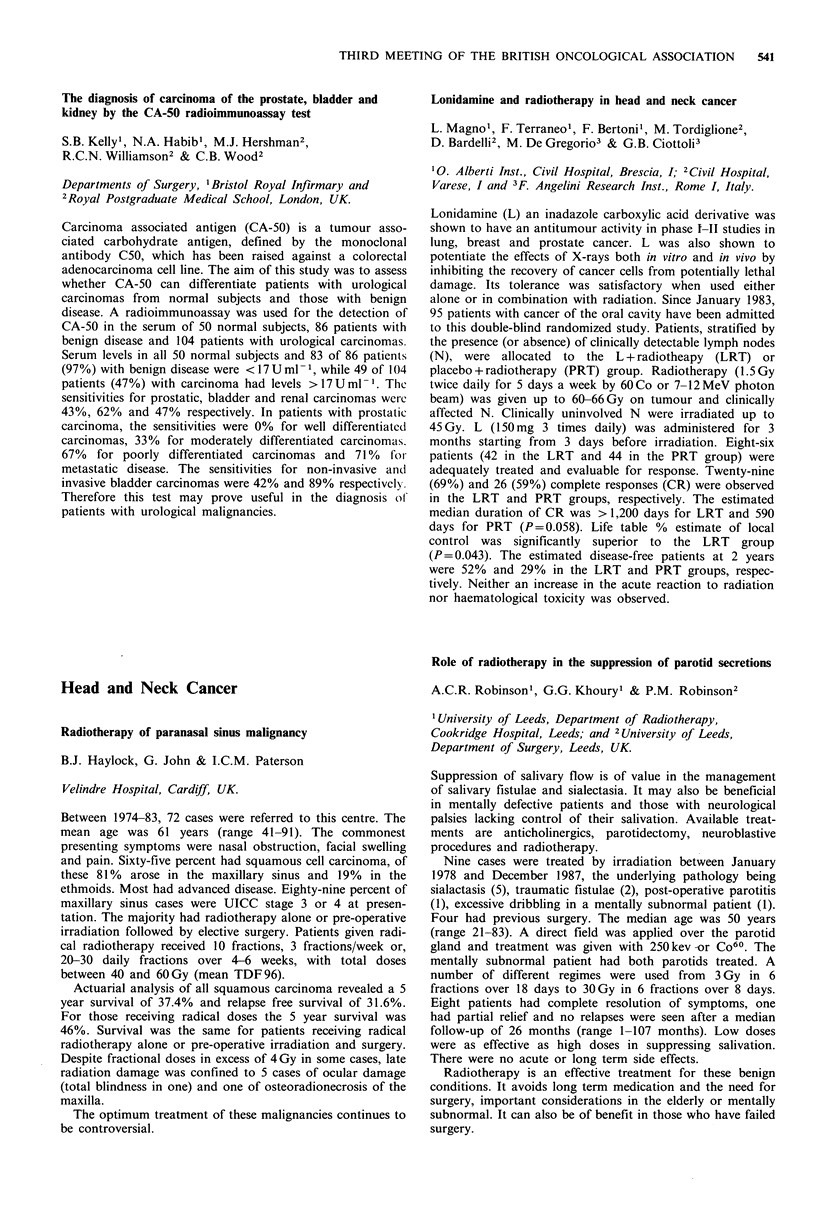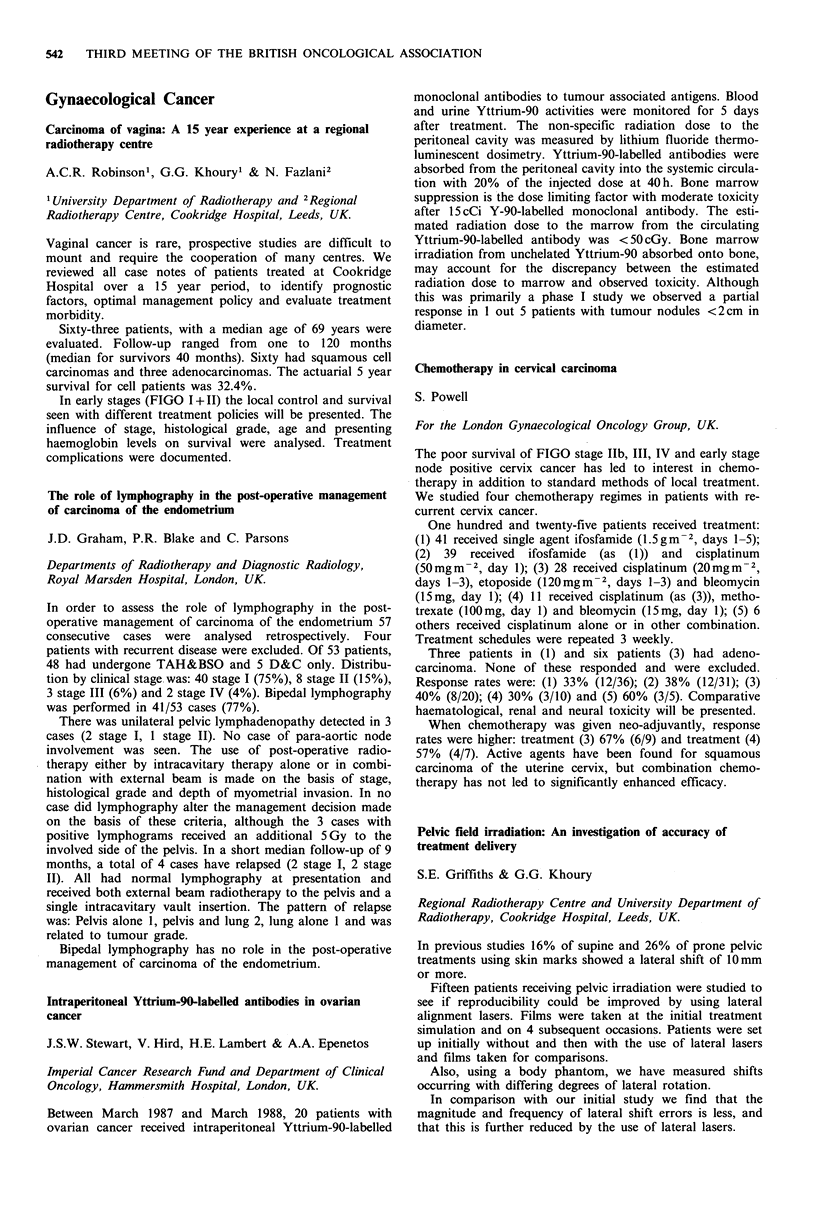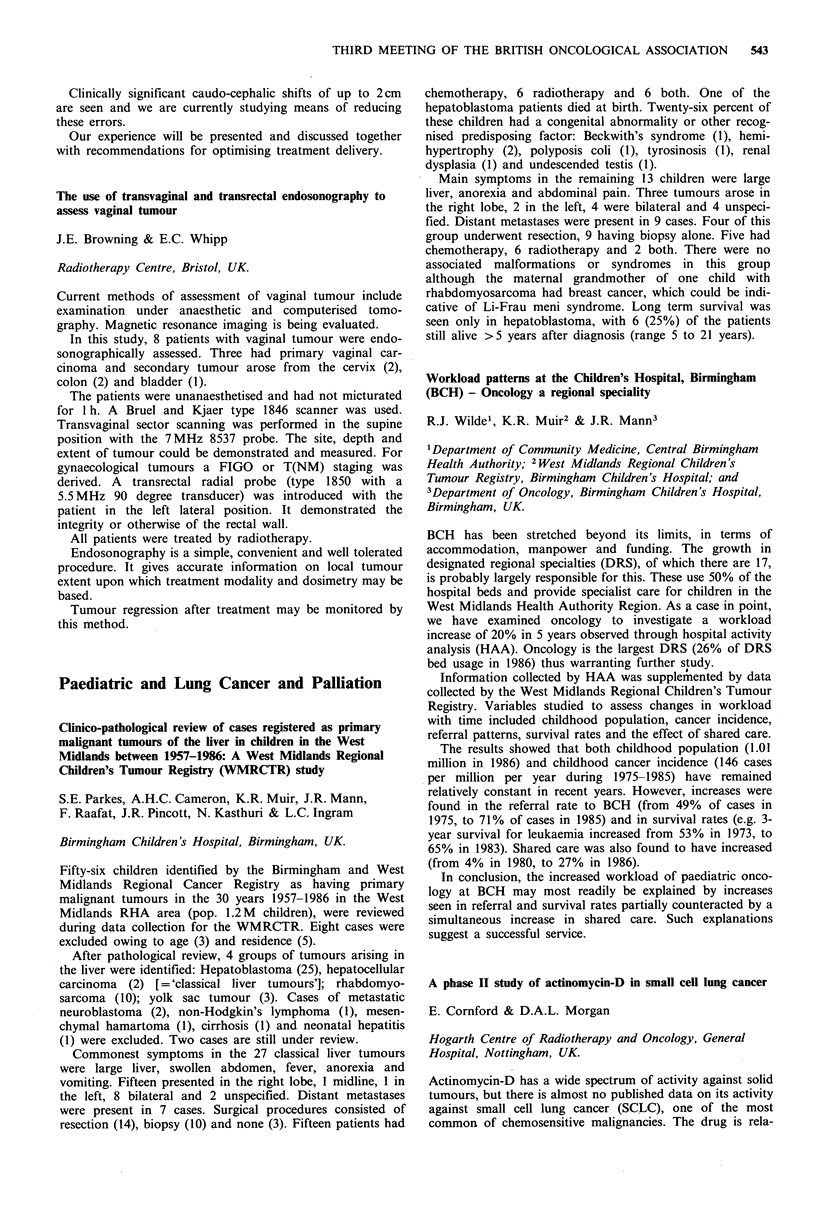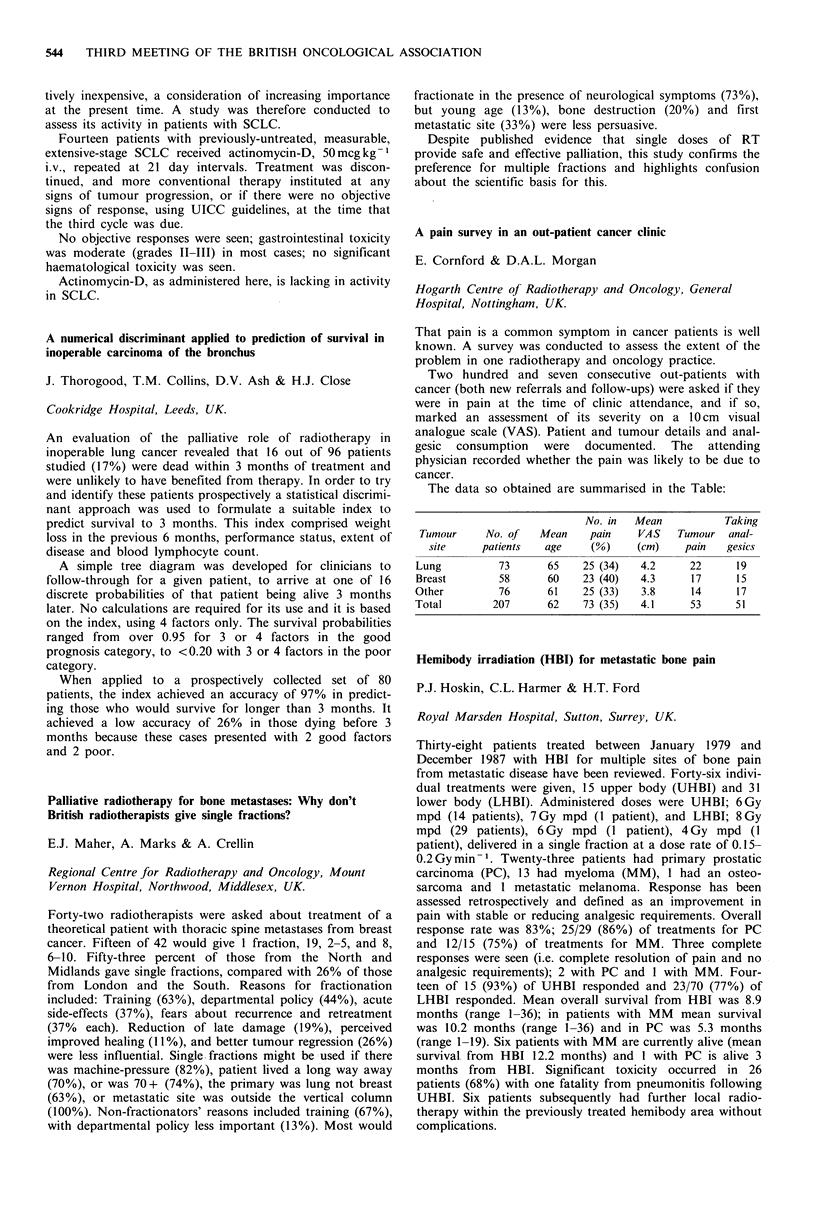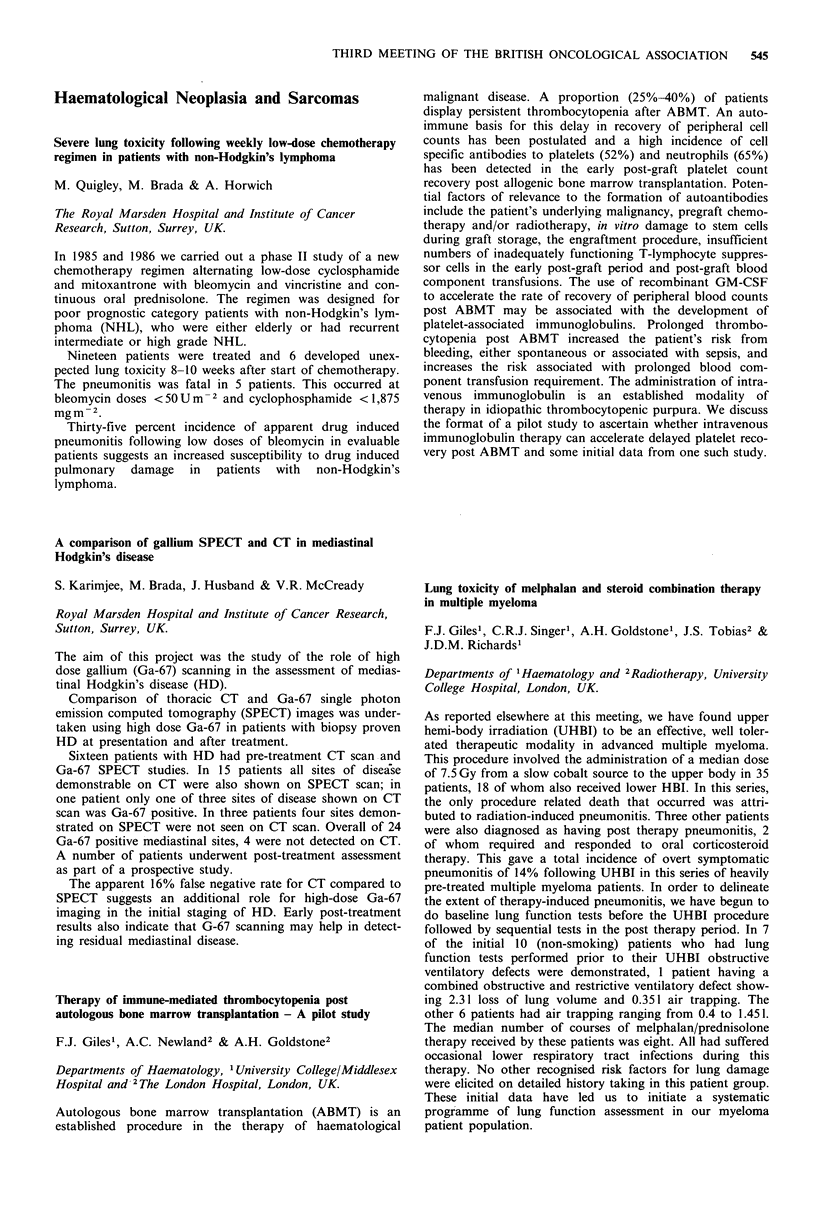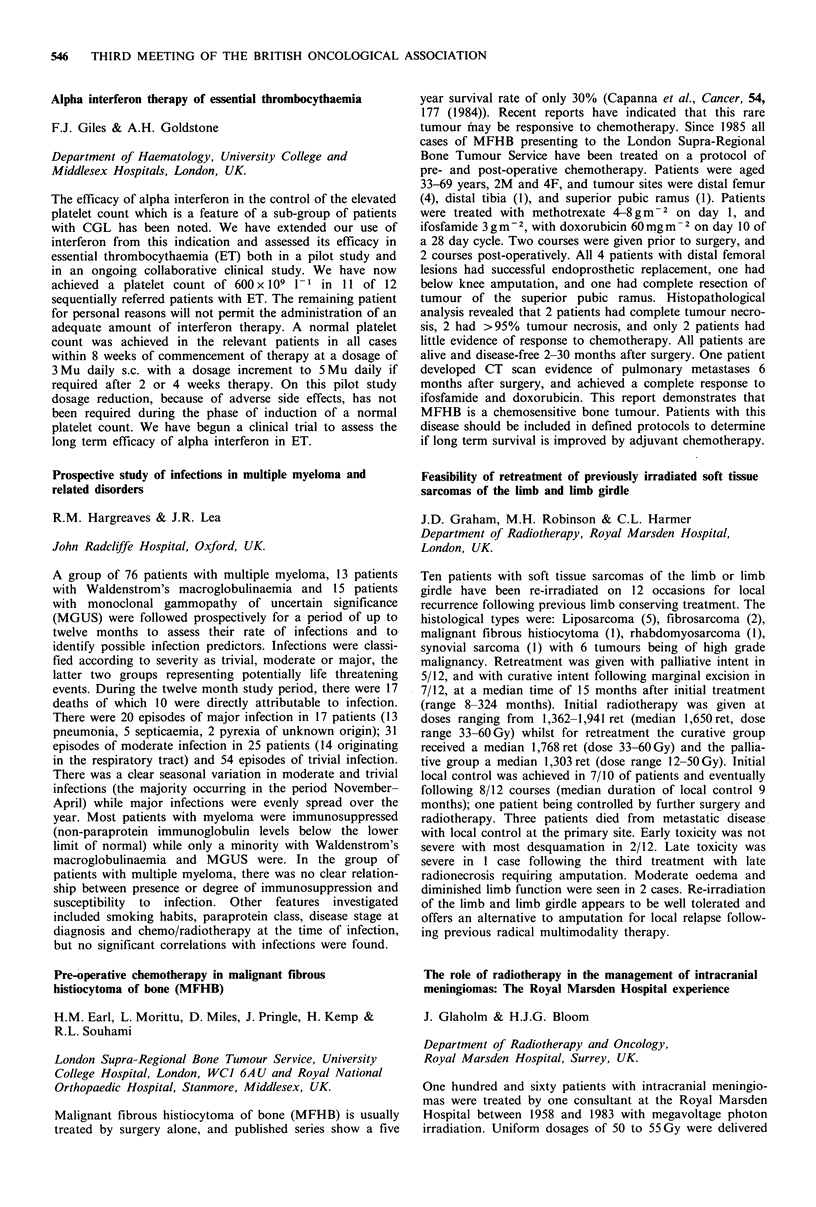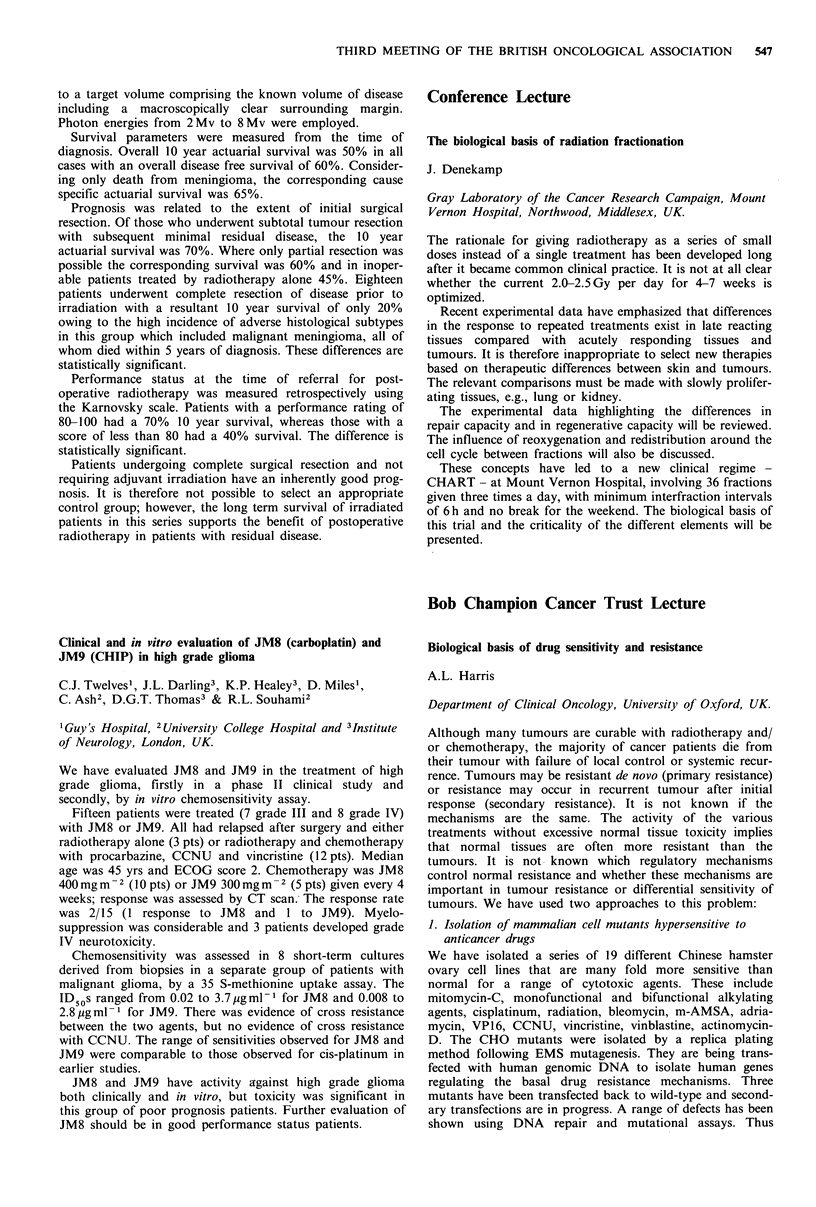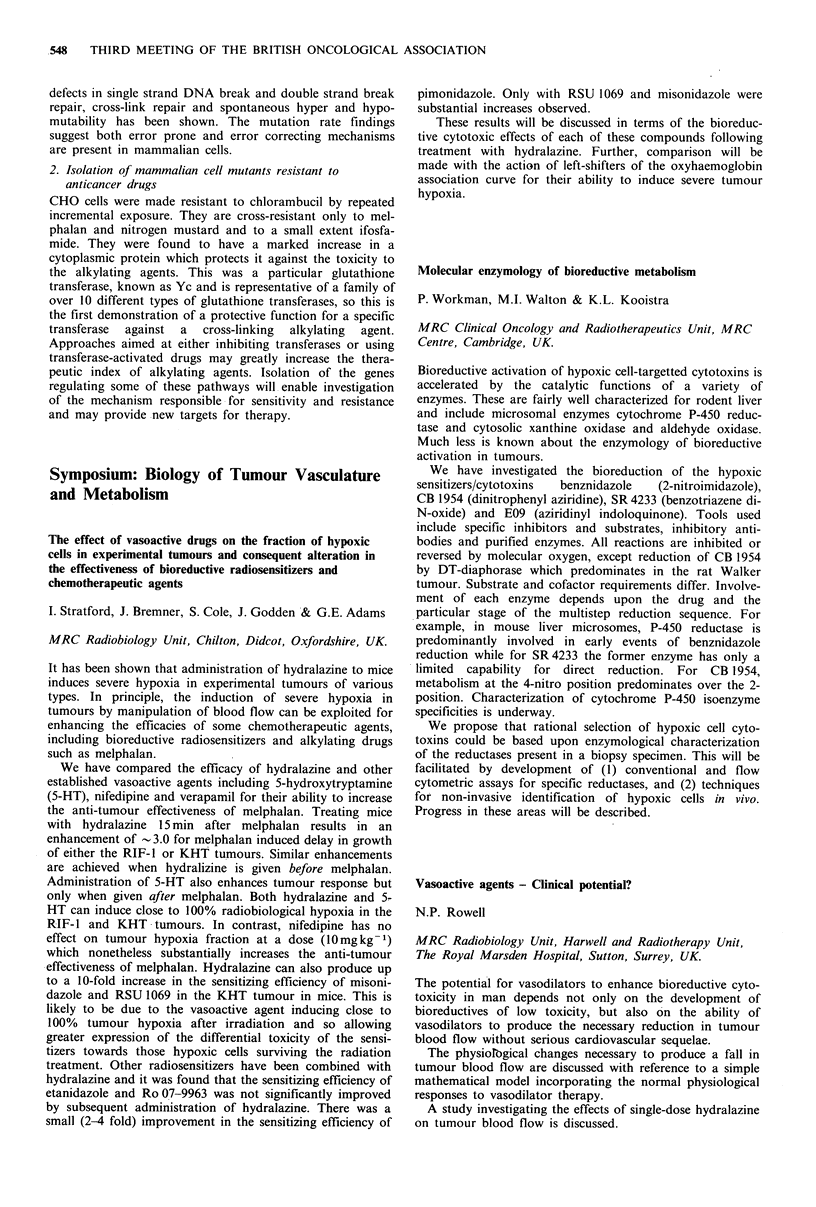# Third meeting of the British Oncological Association. York, 3-5 July, 1988. Abstracts.

**Published:** 1988-10

**Authors:** 


Br. J. Cancer (1988), 58, 518–548 © The Macmillan Press Ltd., 1988

### Third meeting of the British Oncological Association*†

*Held at the College of Ripon and York St. John, Lord Mayor's Walk, York, 3–5 July, 1988*

### Abstracts of proffered papers

### Gynaecological Cancer

### Radical hysterectomy is overtreatment for early stage cervix cancer

E.J. Buxton[1], N. Saunders[2], M.E.L. Patterson[2], C. Hilton[1], D.M. Luesley[3], C.W.E. Redman[1], J.J. Mould[1] & G. Blackledge[1]

[1]*West Midlands Cancer Research Campaign Clinical Trials Unit;* [2]*Northern General Hospital, Sheffield and* [3]*Dudley Road Hospital, Birmingham, UK.*

Radical hysterectomy with pelvic lymphadenectomy has been regarded as the optimal surgical approach in managing early invasive cervix cancer. Pelvic lymphadenectomy has not been shown to improve prognosis in node positive patients and is probably unnecessary in patients with negative nodes. Wide parametrial excision has considerable morbidity. We report a retrospective analysis of surgically managed early stage disease the aims of which were: to define the incidence of parametrial tumour extension and to assess the therapeutic benefit of wide parametrial excision.

The case records and pathology reports of 141 patients who underwent radical hysterectomy with pelvic lymphadenectomy between 1980 and 1987 were reviewed (median follow-up 32 months). Nine (6.3%) had direct tumour extension into the parametrium (5 with positive and 4 with negative nodes). Four of five node positive patients received post operative external beam radiotherapy (EBRT) and three have relapsed (2 with pelvic and distant and 1 with only distant recurrence). In the four node negative patients 2 received post operative EBRT and one has relapsed with pelvic disease. Twenty of the remaining 132 patients were node positive and 112 were node negative. Thirteen with positive nodes received EBRT and three have relapsed (1 with pelvic and 2 with pelvic and distant recurrence). Three of the remaining seven node positive patients have relapsed (2 with pelvic and 1 with pelvic and distant recurrence). Fifteen node negative patients have relapsed (9 with pelvic, 3 with pelvic and distant and 3 with distant recurrence alone). There was no signifiant difference in relapse rate between patients with positive nodes who did or did not receive post operative EBRT.

The only patients who can be regarded as benefiting from wide parametrial excision are those with direct tumour extension to the parametrium who have negative nodes and do not subsequently relapse, i.e. three of 141 patients (2.1%). These data do not support the hypothesis that wide parametrial excision is of therapeutic benefit and question the value of post operative pelvic radiotherapy. There may be potential for management of small volume early stage disease by simple hysterectomy followed by adjuvant therapy where tumour spread to the parametrium or pelvic nodes is found to have occurred.

### Survival of younger cervical carcinoma patients treated by radical radiotherapy in the West of Scotland 1964–1984

E.J. Junior, R.P. Symonds, E.R. Watson & D.W. Lamont

*Beatson Oncology Centre, Glasgow, UK.*

Overall mortality from carcinoma of cervix has declined since the mid 1950s, but the death rate from this disease amongst younger women has risen.

Between 1964 and 1984, 2,011 patients in the West of Scotland were treated by radical radiotherapy for carcinoma of the cervix. The percentage of patients aged <35 years has risen from 4% to 10% in the twenty years, but younger patients have presented with earlier stage disease, unlike the pattern of stage distribution seen in patients aged >45 years.

All patients allocated the same FIGO stage were treated by identical radiotherapeutic technique and dose, irrespective of age or histology. Survival data from at least five year follow up was analysed, relative to stage, age and histological grade.

There was no statistically significant difference in survival for any age group within the same stage category, nor was there any correlation with histological grade, despite an increase in the number of poorly differentiated tumours, especially in women aged <45 years. Overall, younger patients in the West of Scotland have a better prognosis than patients aged over 45 years, mainly due to early presentation.

This analysis suggests that the rise in mortality of younger patients from carcinoma of cervix is due to an increased incidence of the disease in younger women, rather than to a more virulent form of the disease.

### Randomised trial of JM8 (carboplatinum) *versus* JM9 (CHIP) in untreated carcinoma of the ovary

C.W.L. Trask, A.C. Silverstone, R.L. Souhami, H.M. Earl, C.M. Ash, C. Irwin & J.S. Tobias

*Southend Hospital and University College Hospital, London, UK.*

Between August 1984 and October 1987, 120 patients with Stage IC–IV epithelial ovarian cancer were randomly assigned to receive JM8 (400 mg m$^{-2}$ or JM9 (300 mg m$^{-2}$) every 4 weeks as primary therapy. Stratification was made according to FIGO stage and to size of residual disease after surgery.

Following six courses patients were re-staged and response evaluated. Investigations include laparoscopy in clinical complete responders or those with no evaluable disease. Treatment was then stopped in pathological complete responders. Partial responders continued with a further six courses of their original drug at a reduced dose (JM8 300 mg m$^{-2}$, JM9 225 mg m$^{-2}$). Patients with stable, progressive or recurrent

*Enquiries to the BOA Secretariat, The British Postgraduate Medical Federation, 33 Millman Street, London WC1N 3EJ, UK.

†Reprints of these abstracts are not available – Ed.

disease were treated with cyclophosphamide ($1\,gm^{-2}$). There was no cross over between the two arms of the study.

The response rates were 62% for JM8 and 36% for JM9. Sixteen patients were unevaluable for response. The median survival is 96 weeks for JM8 patients and 68 weeks for JM9 patients ($P=0.1319$). The amount of residual disease after initial laparotomy was a prognostic factor for survival. Myelosuppression was the main toxicity and was greater with JM9.

The study shows JM8 to be more active than JM9 in the treatment of ovarian cancer and less toxic. Few responses to cyclophosphamide occurred following either drug, implying resistance to the alkylating agent.

### Intraperitoneal radioimmunotherapy for ovarian cancer. Radiopharmacokinetics, toxicity and efficacy of $^{131}I$ labelled monoclonal antibodies

J.S.W. Stewart, V. Hird, H.E. Lambert & A.A. Epenetos

*Imperial Cancer Research Fund and Department of Clinical Oncology, Hammersmith Hospital, London, UK.*

Thirty-six patients with ovarian cancer were treated with intraperitoneal $^{131}I$ labelled monoclonal antibodies. The activity of $^{131}I$ administered was increased from 20 mCi to 158 mCi and toxicity and efficacy evaluated. Detailed pharmacokinetic studies were undertaken in 11 patients and compared to those of five retreated patients who had developed antibodies against the mouse immunoglobulin. Patients receiving their first treatment had a maximum of 25% (range 19.8–39.8%) of the injected activity in their circulation. This was accompanied by marrow suppression at $^{131}I$ activities over 120 mCi. The five retreated patients had only 5% of the injected activity in their circulation (range 3.8–6%), with rapid urinary excretion and negligible marrow suppression.

In the 31 patients with assessable disease there was no response in eight patients with gross disease (nodules $>2$ cm), a partial response in 2 out of 15 patients with nodules $<2$ cm, and complete response in three out of 6 patients with microscopic residual disease. The non-specific radiation dose to the peritoneal serosa was measured by lithium fluoride thermoluminescent radiation dosimetry in 11 patients. The dose received by the peritoneal serosa was less than 500 cGy.

It is concluded that i.p. $^{131}I$ labelled antibodies may be effective against very small tumour deposits. The local radiation dose to the normal peritoneal serosa is low due to the efficient systemic absorption of antibody. Bone marrow suppression is the dose limiting factor. This may be reduced by administering concurrent systemic anti-mouse immunoglobulin.

### D-TRP-6-LHRH (decapeptyl): A new treatment in advanced ovarian cancer

H. Parmar, R.H. Phillips, G. Rustin & S.L. Lightman

*Charing Cross and Westminster Medical School, Westminster Hospital, London, UK.*

Ovarian cancer cells have been shown to have oestrogen and progesterone receptors and antioestrogen and progesterone have been used in therapy of ovarian cancer. Recently LH (hCG) receptors have been identified in ovarian cancer tissues and *in vitro* studies have demonstrated growth of human ovarian cancer cell lines treated with commercial LH (hCG) preparations from the urine of pregnant females. We treated 39 patients with advanced ovarian cancer (FIGO stage III or IV) who had relapsed following conventional treatment. The D-Trp-6-LHRH microcapsules were administered once a month. There were no exclusion criteria and patients of any age or performance status were eligible. Six patients achieved a partial remission where the tumour decreased in volume more than 50%. The responses were maintained for a mean duration of 10 months (range 6–18 months). Twenty-eight patients continued to have progressive disease and 25 died within the first 16 weeks from the start of therapy reflecting the poor performance status of these patients. Five patients remained stable on D-Trp-6-LHRH for varying periods from 3–12 months. The responding patients had a significantly longer survival than non-responding patients. Therefore, 11 patients (6PR + 5SD) have had remission or stabilization of disease which has been clinically worthwhile. This clinical benefit with slow-release D-Trp-6-LHRH in $\sim$27% of patients is highly encouraging and offers an important non-toxic alternative in patients who are either intolerant of chemotherapy or in those patients who have progressive disease following chemotherapy.

### Head and Neck Cancer

### Production of transforming growth factors alpha and beta by oral squamous cell carcinoma, human skin and cultured keratinocytes

M. Partridge

*Charing Cross Sunley Research Centre, London and Unilever Research, Sharnbrook, Bedford, UK.*

There is evidence for normal and abnormal growth control mechanisms involving growth promoting and inhibiting factors. We have examined a series of oral squamous cell carcinomas (SCC), skin and cultured keratinocytes for the production of transforming growth factor alpha (TGF$\alpha$), transforming growth factor beta (TGF$\beta$) and in some cases for the presence of epidermal growth factor receptor (EGFR). The experiments were designed to investigate whether these factors are produced locally in tissues and hence potentially play a role in normal and malignant cell growth.

Using northern blotting and radioimmunoassay we detected TGF$\alpha$ and $\beta$ in normal skin and cultured keratinocytes. Addition of EGF to cultured keratinocytes resulted in increased secretion of TGF$\alpha$ and $\beta$ suggesting that production of these factors may be regulated by exogenous growth factors.

We find a relationship between the presence of TGF$\alpha$ and its receptor (EGFR) in oral SCC which also suggests an autocrine loop influencing growth of these tumours. An inverse correlation between the level of TGF$\alpha$ detected and EGFR expression was observed. This may be explained by proposing active metabolism and degradation of TGF$\alpha$ via the EGFR in SCC. TGF$\beta$ was also detected in these tumours but evidence suggests that the tumour may be unable to respond to the inhibitory effects of this ligand (Ozanne *et al.*, *J. Pathol.*, **149**, 9, 1986). This lack of normal inhibitory regulatory control may be related to continued tumour growth.

### Human cell kinetic data and the programming of radiotherapy

M.I. Saunders, S. Dische, M.H. Bennett, G. Wilson & N. McNally

*Mount Vernon Hospital, Northwood, UK.*

The administration of bromodeoxyuridine with delayed tumour sampling has given cell kinetic data in 75 human tumours. Conventional views on the rate of growth of human tumours, based on histopathology, must be revised in a number of tumours in the light of this new knowledge.

Verrucal carcinoma is generally regarded as a slowly growing tumour and one difficult to cure by radiotherapy. Four cases will be described where cell kinetic studies have been performed. In all four, cell cycle times were remarkably short indicating that there was a potential for rapid cellular proliferation. This can be contrasted with the apparently slow rate of increase in size associated with such a tumour. Three of these patients were treated by a regime of continuous, hyperfractionated, accelerated radiotherapy in which 36 fractions were given over a period of 12 days (CHART). All have shown a complete regression of tumour. The final case was treated by surgery.

A wide range of head and neck tumours have been treated with the CHART regime and results showed significant improvement when comparison was made with previously treated cases. Cell kinetic studies performed in many of these tumours showed that in most there was a capacity for rapid repopulation. Such a capacity is likely to be expressed once cell destruction occurs on initiation of treatment. Cell kinetic data are likely to be a major influence in the programming of cancer treatment.

**A controlled prospective randomised trial of combination chemotherapy administered synchronously or sequentially with radical radiotherapy: An analysis of prognostic factors in advanced squamous carcinoma of the head and neck**

F.M.B. Calman

*On behalf of the SECOG Trial Steering Committee, UK.*

Between February 1980 and June 1986, 361 patients with advanced (Stages III and IV) squamous cancer of the head and neck were randomised into a trial comparing combination chemotherapy (vincristine, bleomycin and methotrexate) given either synchronously or sequentially with radical radiotherapy. A further randomisation was made between the addition of 5-fluoro-uracil and no 5-fluoro-uracil in a 2-by-2 factorial design. There was no significant difference between the two arms of the trial although patients receiving 5-FU had improved disease-free survival compared with those who did not ($P=0.05$). The results of the study have been analysed by disease site, extent of primary tumour (T), nodal status (N), age, sex and degree of differentiation of tumour. Nodal status was the most significant prognostic factor in patients with advanced disease.

**Salvage surgery following combined chemotherapy and radiotherapy**

W.G. Edwards, H.R. Grant & J.S. Tobias

*SECOG Trial, CRC Clinical Trials Centre, London, UK.*

One hundred and twenty-six patients have undergone salvage surgery following primary treatment with chemotherapy and radiotherapy for stage 3 or 4 squamous carcinoma of the head and neck. The SECOG trial design and 5 year survival data on 429 patients entered is itemised by primary site and chemotherapy arm.

Surgery was limited to histologically proved persisting or recurrent carcinoma. Fifty-four patients received synchronous, and 72 received sequential treatment using VBM or VBMF with full dose megavoltage radiotherapy.

Details of primary site, TNM status, allocated treatment and results of surgery in all 126 patients are itemised. Five year postoperative survival is 33% and a highly significant trend ($P=0.002$) in favour of the older patients is reported.

The range of surgical excision and reconstruction carried out with early postoperative mortality is itemised for this prospective study maintaining complete follow up.

**The role of microorganisms in osteoradionecrosis of the mandible**

I.L. Hutchison & C.C. Kibbler

*Joint Department of Maxillofacial Surgery, Eastman Dental and University College Hospitals, and Clinical Microbiology, University College Hospital, London, UK.*

Two theories for the pathogenesis of osteoradionecrosis of the mandible (ORN) predominate: Firstly, that ORN is analogous to osteomyelitis with microorganisms playing a central role in bone destruction; secondly, that ORN is an aseptic ischaemic necrosis, mediated by endarteritis obliterans, in which microorganisms merely act as superficial contaminants. This study sought to clarify the role of microorganisms in ORN.

Twelve patients with ORN defined as 'an area of exposed mandible in a previously irradiated field that fails to heal for two months' underwent corrective surgery. Bone was debrided until bleeding trabeculae were encountered, and in some cases the residual mandible was covered with local vascularised flaps. The bone removed was collected in plain sterile pots, and jars of Robertson's cooked meat medium. It was then cultured on neomycin blood agar for anaerobic bacteria and *Actinomyces species*, and on Sabouraud's medium for *Candida species*, in addition to routine culture on blood agar.

A mixed growth of mouth flora was cultured from the bone in eight cases, *Candida species* in three cases and *Actinomyces species* were not isolated despite prolonged anaerobic culture. Soft tissue specimens grew *Staphylococcus aureus* in three cases, *Group G Streptococcus* in one case, and anaerobes in one case. Histology specimens showed no permeation of the medulla with microorganisms.

Our results suggest that ORN is not primarily an infective osteomyelitis in which bone destruction progresses rapidly through the medullary cavity, although microorganisms are present. This has important implications for the management of ORN.

**Single fraction radiotherapy for carcinoma of the skin**

S. Chan, J.Lim & R. Hunter

*Christie Hospital and Holt Radium Institute, Manchester, UK.*

Single fraction radiotherapy using superficial X-ray machine has obvious advantages in the treatment of small superficial carcinomas of the skin. We have studied retrospectively 1,005 cases of basal cell carcinoma and squamous cell carcinoma of the skin, treated by a single fraction X-ray therapy in 1976 in this institution to define the tumour recurrence and late radiation necrosis rates with treatment using different radiation doses and different field sizes.

Three radiation doses were used: 22.5 Gy, 20 Gy and 18 Gy. All cases were treated by superficial X-ray machine at 45 kV or 100 kV (10 cm FSD). The cumulative recurrence rate after 10 years of follow-up was 4.4%. The cumulative late radiation necrosis rate was 6% (17% required surgical repair). The relationship between field sizes/radiation dosages and recurrence/necrosis rates were as follows:

| | Diameter of radiation portal (cm) | | | |
|---|---|---|---|---|
| | 1.5–2.0 (N=531) | 2.1–3.0 (N=415) | 3.1–4.0 (N=42) | 4.1–5.0 (N=17) |
| Recurrence (%) | 4 | 4 | 7.7 | 9.1 |
| Necrosis | 5.7 | 7.1 | 10.5 | — |

| | Radiation dosages (Gy) | | |
|---|---|---|---|
| | 18 (N=42) | 20 (N=499) | 22.5 (N=459) |
| Recurrence (%) | 5.1 | 4.6 | 4.1 |
| Necrosis (%) | 2.3 | 3.0 | 9.6 |

These results demonstrate the acceptability of single fraction X-ray therapy for the treatment of these superficial skin cancers, and provide useful clinical criteria for the application of this therapeutic technique. The optimal dosage in this series of treatments seems to be 20 Gy with a maximum field size of 3.0 cm diameter.

### Conference Lecture

### Clinical prospects for hyperthermia

J. Overgaard

*Danish Cancer Society, Department of Experimental Clinical Oncology, Aarhus, Denmark*

Using hyperthermia as an adjuvant to radiotherapy in the treatment of especially locally advanced tumours is based on a well developed biological rationale. A large number of early clinical studies of superficial tumours (e.g. advanced breast, chest wall recurrences, neck nodes, and malignant melanoma) have confirmed this biological rationale. Clinical data have been collected in an attempt to evaluate the potential benefit. An analysis of this material has demonstrated iso-effective thermal enhancement ratios of ~1.5 in the investigated tumour sites. The place for hyperthermia in oncology is, however, still not defined because the heating technique as yet generally is unsatisfactory and conclusive results of controlled clinical trials still are not available. Nevertheless, hyperthermia appears to be a helpful tool in palliative treatment and persistent tumour control has been obtained in tumours recurring in previously irradiated sites. This effect has especially been pronounced when using a combination of interstitial hyperthermia and irradiation. The clinical evaluation has almost solely been limited to superficial tumours whereas external heating of deep-seated tumours is still preliminary and most studies are in Phases I–II with emphasis on toxicity and feasibility. Initial results are promising with regard to improved tumour control and acceptable toxicity.

Although hyperthermia is used clinically, the optimal treatment schedule has not yet been defined and there are significant problems related to fractionation of both hyperthermia and radiation, application of homogeneous heating, and definition of a thermal dose concept. The implications of these problems for a potentiation and optimization of the clinical application will be discussed.

### Proffered papers

### Breast Cancer I

### Cellular DNA changes during the genesis of breast cancer

R. Carpenter, J. Matthews, K. Gotting, R. Nicholson & T. Cooke

*Charing Cross Hospital, London; Royal Liverpool Hospital, Liverpool and Tenovus Institute, Cardiff, UK.*

There are no histological features which predict the biological behaviour of pre-malignant and pre-invasive breast lesions increasingly detected as a result of breast screening. We have investigated changes in cellular DNA content, proliferation index (PI) and ploidy during the evolution of carcinogen induced rat mammary carcinoma.

Following intragastric installation of dimethylbenzanthracene, or saline as control, breast tissue was sampled at 2-weekly intervals, disaggregated, feulgen stained and PI and ploidy assessed using integrating microdensitometry.

Glandular hyperplasia present in 45% of glands was seen more frequently than in controls by the second week of treatment ($P<0.01$). Mean PIs of 7 and 9% were significantly higher in induced breast at 2 and 4 weeks relative to control values of 4.6 and 3.7% ($P<0.04$ and $P<0.03$ respectively). Aneuploid profiles, never seen in controls, first occurred at 4 weeks in 33% of glands, rising to a maximum of 54% by 6 weeks. Carcinoma, evident at 6 weeks, occurred in up to 50% of glands, but was preceded by aneuploidy and abnormally high PI in all cases ($r=0.86$; $P<0.05$ and $r=0.98$, $P<0.01$ respectively).

In this animal model quantitative changes in cellular DNA content precede the appearance of carcinoma, indicating the potential benefit of similar assessment of human pre-invasive breast lesions.

### Regulation of epidermal growth factor receptor synthesis in breast cancer cells

R.E. Leake, W.D. George, D. Godfrey & F. Rinaldi

*Departments of Biochemistry and Surgery, University of Glasgow, Glasgow G12 8QQ, UK.*

Growth regulation of oestrogen receptor (ER) positive cells was thought to be mediated by a direct effect of activated ER complex on the target epithelial cell. Experiments using both primary cell cultures and the ER+ breast cancer cell lines MCF-7 and ZR-75 show a maximum stimulation of oestradiol alone of 50–70% over 4 days in phenol-red containing medium. A combination of prolactin and epidermal growth factor (EGF), under identical conditions, gives a stimulation of 250%. If prolactin is given alone, there is little growth stimulation but the level of available EGF receptor rises by 10-fold over the 4 day growth (expressed per unit DNA). Similar experiments in phenol red-free medium (phenol red contains a weak oestrogen) indicate that exogenous oestradiol (at physiological levels) is required to promote the full effect of prolactin on synthesis of EGF receptor. Cycloheximide (at levels which do not alter control growth rates) completely inhibits the increase in available EGF receptor. Progestins have a significant but smaller effect on both growth rates and EGF receptor synthesis.

Separate experiments show that ~45% of breast cancer biopsies contain significant levels of EGF receptor, measured by a ligand binding assay. However, test of the correlation between extractable EGF (range 0–45 ng ml$^{-1}$) and content of EGF receptor proved negative. EGF receptor-mediated growth *in vivo* may be due to TGF$\alpha$ in breast cancer cells. A

satisfactory assay for tissue content of TGFα is still being developed. Blockage of EGF receptor may have an anti-tumour activity.

### Predictors of local recurrence and progression in patients treated by surgery alone for *in situ* breast carcinoma

P. Price, G. Walsh, R.P. A'Hern, B. Gusterson, H.D. Sinnett, J.R. Yarnold & J.A. Mckinna

*The Breast Unit, The Royal Marsden Hospital, Fulham Road and The Institute of Cancer Research, Sutton, Surrey, UK.*

At the Royal Marsden Hospital, between 1972 and 1982, 62 patients were treated by surgery alone for histologically proven pure duct carcinoma *in situ* (DCIS). Patients were treated either by mastectomy (21), subcutaneous mastectomy (4), or by local excision with clinical and mammographic follow-up (37). Median follow-up was 7 years (range 5 years–14 years). Twenty-four of 62 (39%) patients have recurred locally. Median time to recurrence was 28 months (range 6 months–86 months). Local recurrence on the chest wall occurred in 1/21 (5%) patients having mastectomy and in the chest wall or nipple in 3/4 (75%) patients having subcutaneous mastectomy; 20/37 (54%) patients recurred locally in the breast after conservative surgery and most recurred at or near the original site of disease. Twelve of 24 (50%) patients recurred locally with invasive disease and 2/38 (5%) patients without local recurrence developed metastases.

On univariate analysis, there was a suggestion that patients with an incomplete excision were more likely to recur (hazard ratio 2.6 95% CI 0.82–6.05 $P=0.1$). The factors which correlated with local recurrence on multivariate analysis were *conservative surgery* (hazard ratio 7.69 95% CI 1.01–50 $P=0.02$) *multifocal disease* (hazard ratio 2.40 95% CI 0.97–5.97 $P=0.06$); histological sub-type of disease – *papillary tumours* were less likely to recur (hazard ratio 0.07 95% CI 0.01–0.40 $P=0.002$). *Conservative surgery* was the only factor to correlate with invasive local recurrence (hazard ratio 7.14 95% CI 0.88–50.0 $P=0.07$), although this did not reach conventional statistical significance.

### Does chemotherapy improve survival in advanced breast cancer?

S.R. Ebbs, R.P. A'Hern & M. Baum

*Department of Surgery, King's College Hospital, London, UK.*

The relative efficacies of cytotoxic chemotherapy regimens in the treatment of advanced breast cancer are generally assessed by comparing response rates achieved in randomised trials. Treatment ideally prolongs survival as well as achieving successful palliation. Trials rarely demonstrate a statistically significant survival advantage.

We have tested the hypothesis that chemotherapy has no impact on survival by examining the correlation between response rates and survival in 88 comparisons between arms from the same trial, extracted from 53 published trials of chemotherapy in advanced breast cancer (Powles, T.J. *et al.*, *Lancet*, **i**, 580).

If there were no relationship between response rates and survival one would expect half of the comparisons to show improved survival associated with improved response and half to show decreased survival with improved response. However, in 71% of comparisons the arm with the higher response rate also demonstrated longer median survival. Weighted linear regression showed a statistically significant relationship between response rates and survival ($P<0.01$). The number of patients in a comparison did not influence this relationship.

The equation derived from linear regression allows determination of the likely survival benefit that may be achieved by increasing response rates with more toxic regimens. This model suggests, for example, that increasing response rates from 20% to 40% will increase median survival by 24%, say 16 to 20 months.

### Perioperative cyclophosphamide in the management of early breast cancer: Results from two clinical trials

J. Houghton, D.L. Riley, R. Nissen-Meyer & M. Baum.

*Clinical Trials Centre, Kings College School of Medicine and Dentistry, London, UK.*

Two large randomised clinical trials were designed to evaluate the role of a single course of cyclophosphamide ($5\,mg\,kg^{-1}\,day^{-1}$ i.v.) for 6 days immediately following primary surgery for early breast cancer. The Scandinavian Adjuvant Chemotherapy Study (SACS-1), initiated in 1965, randomised 1,026 patients and has now been followed up for twenty years. Relapse-free survival shows a consistent benefit for patients treated with the perioperative course ($\chi^2=11.05$, $P=0.0009$). The Cancer Research Campaign Adjuvant Breast Trial (CRC II) exactly repeated the SACS-1 trial but in a $2\times2$ design which also allowed assessment of a two year course of tamoxifen. The relapse-free survival at a median follow-up of approximately 4 years shows a trend but no significant benefit for the treated patients ($\chi^2=3.09$).

Data from the two trials have been analysed together; relapse-free survival stratified for menstrual status gave an overall relative risk (RR) of 0.82 ($\chi^2=9.73$, $P=0.002$; premenopausal RR=0.83; postmenopausal RR=0.82). An overall significant advantage was also demonstrated when the data were stratified according to nodal status (RR=0.83, $\chi^2=8.71$), $P=0.003$); the beneficial effect of cyclophosphamide was seen in both node negative and node positive patients although in the latter group the result was not conventionally significant (node negative – RR=0.78; $\chi^2=5.85$, $P=0.016$; node positive – RR=0.86, $\chi^2=3.45$, $P=0.06$).

Since the course was well tolerated by most patients, administration of such therapy should be considered in the management of early breast cancer patients.

### Tumour and Normal Tissue Biology I

### Why are some human tumours more radiosensitive than others?

G.G. Steel & J.H. Peacock

*The Institute of Cancer Research, Sutton, Surrey SM2 5PX, UK.*

There is now good evidence that the radiosensitivity of human tumour cells varies from one tumour type to another, and that the steepness of the initial part of the cell survival curve correlates with clinical radioresponsiveness. Studies at low dose rate allow differences between tumour cells to be seen more clearly. Current mathematical models of radiation cell killing include two components: A linear (i.e. exponential, 'α-component') and a bending component ('β-component'). Repair of radiation damage mainly affects the β-component. Among the 17 human tumour cell lines that we have studied, the average surviving fraction at 2 Gy due to the α-component is 0.44 and that due to the β-component is 0.88. The $\beta$-effect appears to be similar in radiosensitive and radioresistant tumours; among the radiosensitive tumours the $\beta$-effect makes a very small contribution to overall radiosensitivity. Therefore the differences between radiosensitive and radioresistant tumours must be looked for not in repair capacity but in the nature of the $\alpha$-component.

**Cellular radiosensitivity in human tumour cells is determined by initial DNA damage**

A.M. Cassoni, L.R. Kelland, T.J. McMillan, J.H. Peacock & G.G. Steel

*The Institute of Cancer Research, Sutton, Surrey, UK.*

For some years it has been considered that radioresponsiveness of tumour cells is closely related to their recovery proficiency. More recently, on the basis of studies in murine systems, it has been suggested that the level of induced damage, rather than differences in its repair, may be important (Radford, 1986).

We have therefore set out to analyse the extent to which the level of damage induced by irradiation correlates with radiosensitivity in a wide range of human tumours. Nine tumours of differing radiosensitivity were investigated. DNA damage, as double strand breaks (dsb) was measured using neutral filter elution immediately after irradiation at ice temperature and compared with cellular survival obtained by soft agar and monolayer colony forming assays.

The slope of the dsb-induction curve reflected the shape of the corresponding survival curve, with two distinct patterns being seen. In the five most sensitive lines (mean survival at 2 Gy, 0.3) dsb induction was linear with dose. In the four more resistant lines (mean survival at 2 Gy, 0.6) dsb induction was biphasic with an initial shoulder. Between the groups a two-fold difference in dose was required to produce the same DNA damage at doses within the survival range.

These data suggest that differences in radiosensitivity are related to differences in damage induction, without the need to postulate differences in repair capacity.

**Radiation induced cell death by chromatin loss: A model to explain the shape of cell survival curves**

I.R. Campbell & H.M. Warenius

*University of Liverpool Cancer Research Campaign, Department of Radiation Oncology, Clatterbridge Hospital, Wirral, Merseyside, UK.*

Many models have been proposed to explain the relationship between dose of low LET radiation and cell survival. Most of these have utilised hypothetical concepts such as specific intracellular targets for which there is no firm biological basis. The Chadwick–Leenhouts model does have a biological basis of single and double strand DNA breaks, but only fits the infrequent situation of a continuously curving cell survival function.

We propose an alternative model which relates reproductive death of cells by radiation to loss of chromatin at cell division. This loss of chromatin can occur through chromosomal deletions or through the formation of asymmetrical chromosomal exchanges. It is proposed that smaller doses of radiation produce fewer chromatin breaks, which are more likely to be accurately repaired, compared to larger doses. Consequently, smaller doses of radiation are less efficient in causing cell death, leading to a shoulder on the cell survival curve. Much experimental evidence is in support of this model, and the fit between the derived formula described in this paper and experimental cell survival curves is good. The derived formula approximates to the linear-quadratic equation at low doses of radiation.

**Strategies for the evaluation of drug resistance in human tumour cells**

S.A. Morgan, J.V. Watson & P.J. Smith

*MRC Clinical Oncology and Radiotherapeutics Unit, MRC Centre, Hills Road, Cambridge CB2 2QH, UK.*

Mechanisms of chemotherapeutic drug resistance which have been identified *in vitro* include modified cellular uptake/retention of drug (e.g. membrane changes leading to multidrug resistance: MDR) and variation in intracellular target molecules, e.g. DNA topoisomerase II (topo II) modifications conferring resistance to topo II-interactive drugs such as VP16-213 and m-AMSA. We have evaluated techniques for the identification of such mechanisms in a human small cell lung cancer (SCLC) line (NCI-H69/P), its *in vitro*-derived resistant variant (NCI-H69/LX4), and fresh human tissue obtained by lymph node biopsy of 3 patients with SCLC. Uptake of the DNA-specific fluorochrome, Hoechst dye 33342 (Ho342) (which mimics in its cellular exclusion properties the uptake and loss of drugs involved in MDR) was assessed using flow cytometry (FCM) allowing quantitative measurement of DNA-Ho342 interaction, and identification of subpopulations of cells within biopsy material. Topo II-DNA cross-linking activity was assayed in nuclear protein extracts using mAMSA and VP16. Results with nuclear extracts of NCI-H69/LX4 did not suggest abnormal DNA topoisomerase expression whereas FCM analysis clearly demonstrated reduced Ho342 uptake. This partially reversed with the MDR modifier verapamil. Biopsy-obtained SCLC in 2 cases showed reduced Ho342 uptake, compared to NCI-H69/P, which could be reversed by pre-exposure to verapamil, but topo II activity did not differ significantly from H69. We conclude that FCM analysis of human tumour biopsies can be used to evaluate drug uptake patterns with the advantage modifier responsiveness can be quantitated rapidly and the characterisation of tumour subpopulations followed.

**The role of glutathione metabolism in cytotoxic drug resistance, and cross-resistance to radiotherapy – Implications for combined modality treatment**

R.A. Britten & J.A. Green

*CRC Department of Radiation Oncology, Clatterbridge, Wirral, UK.*

The use of sequential cytotoxic chemotherapy and radiotherapy, following adequate surgical debulking of advanced human ovarian carcinoma is currently under investigation in our department. Cellular glutathione (GSH), and glutathione-S-transferase (GST) are important determinants of cellular resistance to both cytotoxic chemotherapy, and radiotherapy. Thus prior exposure to chemotherapy, and the associated emergence of chemoresistance, may by virtue of elevated levels of glutathione and GST, predispose to the development of radioresistance in tumours.

The relationship between GST, GSH, GSSG and chemoresistance has been established in the human ovarian adenocarcinoma OAW42 cell line, and a chemoresistant sub-line, OAW42/MER. The resistant OAW42/MER line was 2-fold more resistant to melphalan compared to the parent OAW42 cells. BSO potentiated melphalan cytotoxicity by 2–5-fold, while OTZ reduced cytotoxicity by 1.5–2-fold. The response of the melphalan sensitive and resistant OAW42 and 2780 cell sub-lines to photon and neutron irradiation is at present under investigation.

Concurrent with this work a prospective study of GSH and GST levels in normal and malignant human ovarian biopsies has been undertaken. Preliminary results from 22 patients would suggest elevated GSSG and GST levels in tissue from pre-treated patients, and in those biopsies which are hyperdiploid. Altered thiol metabolism may therefore account for the failure of treatment approaches employing sequential chemotherapy and radiotherapy.

### Proffered papers

### Breast Cancer II

**British Oncological Association Lecture**

### The genetics of colorectal cancer

W.F. Bodmer

*Imperial Cancer Research Fund, London, UK.*

Familial adenomatous polyposis (FAP – also called familial polyposis coli) is a rare dominantly inherited susceptibility to colon cancer. Following up a case report of an interstitial deletion of chromosome 5 in a mentally retarded individual with multiple developmental abnormalities and FAP we have shown that the FAP gene is on chromosome 5, most probably near bands 5q21–5q22. Subsequent studies by others have confirmed this linkage and the data suggest that the probe (C11 P11) may be less than 2% recombination away from FAP. This now provides the basis for the use of long range DNA analysis techniques to isolate the FAP gene.

In a parallel study following the approach of Cavanee *et al.* highly polymorphic probes on chromosome 5 were used to search for allele loss in sporadic tumours as compared to normal tissue from the same individual. The data showed that at least 20% and possibly up to 40% of sporadic colorectal tumours become homo- or hemizygous for chromosome 5 markers, indicating that the FAP gene becomes recessive in a relatively high proportion of colorectal carcinomas. This is the first clear example of a recessive genetic defect in one of the commonest cancers.

Polyps from FAP patients do not show chromosome allele loss. The discovery of dominantly acting oncogenes controlling growth factors or their receptors or regulators of their expression, and of recessive genetic defects which may exert dominant control over differentiation through appropriate receptors and ligands suggests a general scheme that can explain many aspects of dominant and recessive action at the cellular level in the development of tumours.

### Altered fractionation in early breast cancer – a randomised trial

J.R. Owen and M.B. Barton

*Radiotherapy Department, Cheltenham General Hospital, Cheltenham, Glos., UK.*

Local excision and radiotherapy have become the treatment of choice in early breast cancer. Approximately 80% of new cases are eligible and this proportion may increase with the advent of screening services. With the greatly increased load on radiotherapy departments some centres are already using 3 fraction/week or 5 fraction/fortnight regimens despite theoretical evidence to suggest an adverse effect on late tissue damage from a larger dose-per-fraction size. Psychological morbidity may also be affected by the number of visits to a radiotherapy department. There has been no prospective clinical test of this policy.

We report a prospective trial of lumpectomy followed by irradiation of the whole breast and lymphatics. Two hundred and eleven patients were randomised to receive one of three regimens, each with a TDF of 70;

1. 45.00 Gy in 25 fractions over 5 weeks – 69 patients
2. 40.00 Gy in 15 fractions over 5 weeks – 71 patients
3. 39.00 Gy in 13 fractions over 5 weeks – 71 patients.

All then received a boost to the primary (16.00 Gy), axilla (10.00 to 16.00 Gy) and IMC (10.00 Gy). With a median follow-up of 2.6 years there is no significant difference in the actuarial survival, disease-free survival or local control rates. Cosmesis was assessed with an observer based 4 point scale. While the percentage with a poor score gradually increased with time there was no significant difference between the 3 treatment groups. There was a slightly higher rate of arm oedema in the 39 Gy group (8/71) but only in comparison with the 40 Gy group (1/71) was this of borderline significance ($0.01 < P < 0.05$).

Within the limits of the power and design of this study no clinically significant difference has been shown between the 3 fractionation regimens. We are now undertaking a further prospective randomised trial to better define the nature of any late changes in cosmetic and psychological outcome.

### Influence of breast size on late radiation reaction

A.Y. Rostom & J. Brierley

*Radiotherapy Department, St. Luke's Hospital, Guildford, Surrey, UK.*

Late radiation reactions were assessed in 129 patients with clinical stage I and II breast cancer. The primary treatment consisted of wide local excision and radiotherapy. Four fields were used, i.e., supraclavicular and anterior axillary, parasternal and a pair of glancing fields to the breast. A maximum dose of 4,875 cGy was given in 15 fractions over 39 days. Assessment of late reaction was made 26/120 months from the end of radiotherapy. Brassiere size (cup and bust) at the time of radiotherapy was known for all patients. The cosmetic appearance and breast consistency was graded as: grade I normal, grade III severe distortion, i.e., in appearance and consistency, grade II moderate, i.e., any reaction between I and III.

No severe reaction was seen. There was no significant difference in the incidence of reaction in relation to cup size. However, there was highly statistical significant difference in the incidence of reaction in relation to bust size. No reactions were noted in women with brassiere size of 36A or under. Moderate reaction was noted in 6 out of 42 patients, brassiere size 36B–36D and in 16 of 44 patients, brassiere size 38A and over. We are continuing to use this fractionation for women with brassiere sizes up to 36B. Women over that size are being treated on a daily basis.

### A prospective randomised trial comparing bolus with prolonged infusion of epirubicin for advanced breast cancer

S.R. Ebbs, H. Graham, T. Bates & M. Baum

*Departments of Surgery, King's College Hospital, London and William Harvey Hospital, Ashford, Kent, UK.*

It has been proposed that prolonged exposure to cytotoxics will improve the therapeutic index by avoiding high drug levels associated with toxicity and also by increasing cell kill of cycle specific agents. We have treated this hypothesis in a clinical trial.

Sixty-two women were randomised to receive weekly single agent chemotherapy (epirubicin) for advanced breast cancer by either bolus injection or prolonged intravenous infusion over 24h. Forty-eight are at present eligible for analysis.

| | *Bolus* | *Infusion* | *Significance* |
|---|---|---|---|
| Number | 24 | 24 | |
| Complete/partial response | 11 (46%) | 3 (13%) | $P=0.03^a$ |
| Nausea or vomiting | 13 | 5 | $P=0.02^a$ |
| Myelosuppression | 8 | 5 | NS |
| Alopecia | 14 | 6 | $P=0.04^a$ |

[a]Chi-square test with Yates continuity correction.

Both survival and time to treatment failure were worse for the infusion arm ($P=0.005$, $P=0.07$*).

Whilst infusion therapy reduces side effects of epirubicin it does so at the expense of a loss of efficacy.

*Peto log rank test.

### Prognosis following omental transportation for locally advanced and recurrent breast cancer

R.J.L. Williams & H. White

*The Royal Marsden Hospital, London, UK.*

An analysis was carried out of 43 patients undergoing omental transposition for locoregional problems associated with breast cancer. Indications for surgery included advanced primary tumour (5), recurrent tumour (32), radiation induced sarcoma (2), and radionecrosis (4). Tumours were typically extensive (mean diameter 7.2cms) and skin ulceration affected 30 of these cases. Other treatment modalities had for the most part been exhausted.

Surgical excision followed by reconstruction using transposed omentum resulted in worthwhile local control and symptom relief in 31 patients (median duration 22 months). Chest wall disease rapidly recurred peripheral to the omental graft in 12 patients. On multiple regression analysis, duration of local control was related to tumour diameter ($P\ll0.001$), ulceration ($P<0.005$), earlier radioresistance ($P<0.02$), and the time elapsed from cancer diagnosis to operation ($P<0.1$). Survival (median 21, range 1.5–122 months) correlated with tumour size ($P<0.001$), prior chemotherapy ($P<0.01$), and early re-recurrence ($P<0.005$).

Omental transfer provides a reliable basis for restoring epithelial cover after radical surgery, particularly useful after previous irradiation injury. Applied to advanced and recurrent breast cancer, an aggressive surgical approach significantly improved the quality of life of most patients. Careful case selection is required to avoid pointless surgery in irremediable tumours.

### Comparison of outcome between mastectomy and lumpectomy patients in an Irish survey of breast cancer

M.W.H. Timms[1], D. Hollywood[2], M. Moriarty[2] & J. Cullen[3]

[1]*Newcastle Hospital, Greystones, Wicklow;* [2]*St. Luke's Hospital, Dublin 6; and* [3]*Psychomatic Unit, St. James's Hospital, Dublin 8, Eire.*

The paper reports data from a study of breast cancer patients attending a radiotherapy department. The methodology is presented, outcome being measured using both a patient self-report inventory (the psychosocial adjustment to illness scale) and observer ratings (the Spitzer quality of life index). The data relating to surgical intervention is then presented. A group of 100 patients seen as consecutive attenders at a radiotherapist's clinic were interviewed: the sample was varied as to age, elapsed time since diagnosis, and present clinical state (new, clear, recurrent). A higher proportion of mastectomy patients were of lower socio-economic status and from rural areas (more remote from the treatment centre). There were no differences in self-rated psychological distress between those from the mastectomy and those from the lumpectomy groups. However, the lumpectomy patients had a better quality of life rating, in spite of the fact that, as a group, they had had their surgery in the more recent past.

### Does radiotherapy for breast cancer produce psychiatric morbidity?

K. Barltrop & K.R. Durrant.

*Departments of Community Medicine and Radiotherapy and Oncology, Radcliffe Infirmary and Churchill Hospital, Oxford, UK.*

Patients with early breast cancer are highly prone to severe anxiety, depression, and sexual problems. The psychiatric morbidity of breast surgery has previously been evaluated. This study was undertaken to determine the pattern and degree of physical and psychiatric morbidity associated with radical radiation therapy for early breast cancer.

Twenty-nine patients with early operable breast cancer were assessed. Fourteen had undergone mastectomy and 15 had been treated by wide local excision. Anxiety and depression were assessed using the Leeds Self-Assessment Scale, the Spielberger State-Trait Anxiety Inventory, and the MRC General Health Card, which also was used to determine physical side-effects.

Twenty-eight percent of patients were clinically anxious or depressed before the start of radiotherapy, 17% at three weeks post-radiation, and 24% at three months. State anxiety was highest at the beginning of the course. Although psychiatric morbidity in patients undergoing irradiation for breast cancer is high, the results of this study do not support the concept that radiotherapy *per se* increases the incidence of clinically significant anxiety or depression.

### Genito-urinary Cancer

### Tumour proliferation rate as a predictive factor in testicular teratomas

P. Price, S.J. Hogan & A. Horwich

*Institute of Cancer Research and Royal Marsden Hospital, Surrey, UK.*

At the Royal Marsden Hospital, between 1984 and 1987, 158 patients with metastatic testicular tumours have been treated using BEP combination chemotherapy (bleomycin, etoposide and cisplatinum) as first line treatment. Twenty (13%) patients have died. Tumour proliferation rates prior to chemotherapy measured by the rate of rise of tumour marker production (TMP) were calculated and correlated with prognosis. One hundred and nine of 142 (76%) patients were serum marker positive, either for HCG, AFP or both. Fifty-four patients had three or more sequential serum marker levels recorded after orchidectomy and before the start of chemotherapy from which the change in rate of TMP per day by the tumour could be determined. The rate of TMP (IU/L/day) took into account the continuing clearance of marker from the serum. The rate of TMP rise was exponential. The rate of this rise was expressed as the rate of marker production doubling time and is a measure of the tumour proliferation rate.

Rates of marker production doubling time varied from 0.5 days to 43 days (34 cases) for AFP and from 3 days to 27.5 days (25 cases) for HCG. The doubling times appeared to be independent of the tumour stage. In the AFP marker group three patients died and all had doubling times $\leqslant$6.5 days ($P=0.136$). In the HCG marker group, four patients died and all had doubling times $\leqslant$5.2 days ($P=0.0226$). Rapid tumour proliferation rates reflected by short TMP doubling times may carry a poor prognosis in patients with testicular teratomas treated by chemotherapy.

**A multicentre randomised trial comparing the LHRH agonist 'zoladex' with 'zoladex' in combination with flutamide in the treatment of advanced prostate cancer**

F. Daniel & C.J. Tyrrell

*Department of Radiotherapy, Freedom Fields Hospital, Plymouth, U.K, on behalf of the 'International Prostate Cancer Study Group'*

Between April 1986 and May 1987, 562 patients were recruited from ten countries into a multicentre, randomised trial comparing 'zoladex' with a combination of 'zoladex' and the nonsteroidal antiandrogen flutamide. 'Zoladex' was administered as a depot injection every 28 days and flutamide was given as three 250 mg tablets daily.

Patients with histologically confirmed locally advanced (T3/T4) or metastatic (M1) carcinoma were included. Previous hormone therapy, anti-hormone therapy, chemotherapy or patients having had an orchidectomy were excluded.

Fifty-five percent of the patients had M1 disease, the remaining being locally advanced disease. The two groups were balanced between major demographic parameters (T category, histology, metastases, performance status, etc.).

The median duration of follow up is 34 weeks in the zoladex group and 28 weeks in the combined treatment group.

The objective and subjective response rates are similar in both groups, but the median time to treatment failure is significantly longer in the zoladex group, largely due to the greater number of withdrawals because of the adverse reaction from the combined treatment ($P=0.004$).

**BEP chemotherapy for metastatic teratoma: Royal Marsden Hospital experience 1979–1986**

D.P. Dearnaley, J. Nicholls, M. Saunders, M.J. Peckham, W. Hendry & A. Horwich

*Institute of Cancer Research and Royal Marsden Hospital, Sutton, Surrey, UK.*

One hundred and twenty-eight previously untreated patients with metastatic testicular teratoma have been given BEP chemotherapy (cisplatinum 20 mg m$^{-2}$ days 1–5, etoposide 120 mg m$^{-2}$ days 1–3, bleomycin 30 mg days 2, 9, 16) between 1979 and 1986. Mean follow up from commencement of treatment is 51 months (range 14 m–84 m). The Royal Marsden Hospital staging system was used and patients divided into prognostic groups as defined by the MRC working party (*Lancet*, 1985). Ninety cases had small volume (15 with high markers), 22 cases large volume (10 with high markers) and 16 very large volume disease (10 with high markers).

Disease free/survival results are for small volume disease, 94%/99%; large volume disease 73%/73%; very large volume disease 25%/31%. Residual masses were resected in 50 patients. White count nadir <1,000 mm$^{-3}$ occurred in 3%, platelet nadir <50,000 mm$^{-3}$ in 2% and Hb <9.5 g 100 ml$^{-1}$ in 15% of 482 chemotherapy courses. Septicaemia occurred in 4 patients with 1 fatality. Symptomatic bleomycin lung damage was reported in 22 patients. Steroid treatment was necessary in only 4 cases, with one death. Mean fall in EDTA renal clearance after 4 courses of chemotherapy was 26 ml min$^{-1}$ (range +78 to −81 ml min$^{-1}$). Twenty-four pregnancies have produced 21 normal offspring.

The identification of risk related factors has enabled the design of chemotherapy regimes of reduced toxicity in good prognostic groups, whilst intensifying treatment in high risk cases. This series will permit future comparison with such sequential studies.

**Accelerated fractionation (AF) for bladder cancer**

A. Horwich, G. Duchesne, D. Dearnaley & G.G. Steel

*The Institute of Cancer Research and the Royal Marsden Hospital, Sutton, Surrey, UK.*

A pilot study was performed to define the acute toxicity of AF in the radical treatment of bladder cancer. Treatment was in 2 phases, treating the bladder to 60–64 Gy in 30–32 fractions over 4 weeks and the upper pelvic nodes to 44 Gy in 22 fractions over 4 weeks. The schedule employed 2 fractions per day on 16 treatment days, with a minimum of a 6 hour gap between fractions. Of 16 patients entering the study, 15 completed radiotherapy according to protocol. One developed subacute bowel obstruction after 44 Gy, which was managed conservatively. The pattern of toxicity in the other patients was as follows: Mild diarrhoea in 11/15 (73%) typically lasting 3 or 4 days in the 2nd week of treatment and 7–10 days following completion; frequency of micturition with nocturia $>\times 5$ in 10/15 (67%) and dysuria in 7/15 (47%) appeared during the 4th week and lasted 1–2 weeks after the end of treatment. The only non-transient toxic reaction has been in a further patient who had relatively mild side effects during radiotherapy but who developed subacute bowel obstruction 2 months later, which was treated by defunctioning colostomy. The median follow up following AF is 6 months and it is too soon to define late side effects, though none have yet been seen.

It seems likely that AF is associated with increased acute reactions but the schedule of 64 Gy in 32 fractions in 4 weeks was tolerated by most patients, and should be investigated in a prospective randomised trial.

### Risk factors for predicting response of metastatic transitional cell carcinoma to chemotherapy

R.T.D. Oliver, R. Iles, D. Crosby & J.P. Blandy

*The London Hospital Medical College, UK.*

One hundred and eight patients (previously untreated with chemotherapy and having bidimensionally measurable metastatic urothelial malignancy) have been entered into sequential chemotherapy trials between 1977 and 1988. Complete response occurred in 1 of 23 receiving methotrexate (M), 0 of 15 receiving cisplatin (P), 0 of 20 receiving carboplatin, 3 of 20 receiving M+P, 5 of 12 receiving M+ vinblastine+P, and 2 of 8 receiving M+V+adriamycin+P. Overall complete plus partial response rate was higher in the M+P, MVP and MVAC groups (75%), though so was toxicity with 35% of patients experiencing temporary major renal failure episodes. Although WHO/UICC performance status 3 and 4 patients had a lower incidence and shorter duration of response, it did not provide a means of excluding patients who achieved complete remission.

Studies using serial urine cytology showed that 7 of 14 responders had complete loss of malignant cells from the urine compared to none of 21 non-responders and there was a suggestion that duration of overall clinical response was longer in those showing a cytological response. Another factor influencing response was expression of BhCG. Elevated serum levels of BhCG were found in 1 of 18 T1M0, 0 of 23 T2,3,4 M0, 0 of 4 T×N1−4M0, and 9 of 12 T×N×M1 tumours. None of 9 who were BhCG positive N×M1 patients responded to chemotherapy, in contrast to 3 of 4 with BhCG negative lymphnode metastases and 2 of 3 with BhCG negative distant metastases.

### Outcome of radiotherapy randomization in the Third National Wilms' Tumour Study (NWTS-3)

P.R.M. Thomas, M. Tefft, G.J. D'Angio & P. Norkool

*For the National Wilms' Tumour Study Committee, Philadelphia, USA.*

In NWTS-3, patients with stage II favourable histology (FH) tumours were randomized to receive either no radiotherapy (RT) or 2,000 cGy to the tumour bed postoperatively. Stage II FH children were randomized between 1,000 cGy and 2,000 cGy. RT boosts were allowable but given to only 6 of 280 (2%) stage III and none of the 280 stage II patients. For both stages there was also a chemotherapy (CT) randomization to either intensified actinomycin D (AMD) plus vincristine (VCR) or to less intensive AMD plus VCR plus doxorubicin (ADR). No statistically significant differences were noted for abdominal relapses or disease-free survival between any RT set. Stage III patients treated with three drugs had a borderline statistical advantage for disease-free survival ($P=0.06$). We conclude that 1,000 cGy is enough for stage III patients if three drugs are used.

### Haematological Neoplasia and Sarcomas

### The BNLI study of LOPP *vs.* LOPP/EVAP in advanced Hodgkin's disease – a progress report

G.V. Hudson & B.W. Hancock

*For the British National Lymphoma Group, UK.*

Previous BNLI studies in advanced Hodgkin's disease have shown: (1) MOPP is more effective than MOP (prednisone omitted), (2) addition of bleomycin to MOPP is of no benefit, (3) maintenance with CCNU (lomustine), vinblastine and bleomycin is of no benefit and (4) LOPP (leukeran substituted for mustine) is as effective yet much less toxic than MOPP.

In the current study in advanced Hodgkin's disease started in June 1983, patients are randomised to LOPP or LOPP alternating with EVAP (etoposide, vinblastine, adriamycin, prednisolone). Over 400 patients have been entered so far; 317 are included in this analysis. The CR rate is better for LOPP/EVAP than for LOPP (69 and 60% respectively) as is the actuarial percentage disease-free survival at two years (60 and 44% respectively; $P<0.05$). This advantage in favour of the alternating regimen does not appear to be due to a lower frequency of poor prognostic factors in this arm compared to the LOPP arm. The difference was maintained for other acknowledged individual prognostic factors. At present there is no survival advantage from the alternating treatment (84% overall survival at 2 years in both arms). Apart from an increased infection rate associated with more severe and unpredictable myelosuppression, and invariable alopecia, the LOPP/EVAP regimen has been well tolerated by most patients.

### Hemi-body irradiation for melphalan resistant multiple myeloma: Experience in 41 patients

C.R.J. Singer, J.S. Tobias, F. Giles & J.D.M. Richards

*Departments of Haematology and Radiotherapy, University College Hospital, Gower Street, London, UK.*

Treatment of patients with resistant myeloma is often difficult because age and physical condition often restricts their ability to tolerate more intensive chemotherapeutic regimens. We describe our experience of hemi-body irradiation [HBI] in 41 patients with resistant multiple myeloma.

Patients were staged using the MRC prognostic criteria: 2 patients were in group I, 18 patients in group II and 21 patients in group III. Age and sex distribution were similar in each group. Each group had received similar amounts of chemotherapy prior to HBI (median 8 courses of melphalan and prednisolone), 10 patients had also failed second line chemotherapy. Twenty-three patients had previously received local radiotherapy. Hemi-body irradiation was administered using a single fraction cobalt source at a dose rate of $0.25\,Gy\,min^{-1}$ at median doses of 7.5 Gy (upper half) and 9 Gy (lower half) with a median interval of 9 weeks in those receiving double HBI. Seventeen patients had double HBI, only 1 patient in whom a second treatment was planned refused it and 3 deteriorated before it could be carried out.

Twenty-one of 23 evaluable patients had a significant IgG or IgA paraprotein response which was better in those patients undergoing double HBI (54%>50% reduction). Subjective improvement was reported in 69%, Bence-Jones protein disappeared in 5 patients and immune-paresis normalised in 2 following HBI. A median survival of 17 months was obtained in group II patients but only 3.5 months in group III. Both patients in group I survive in plateau phase more than 40 months after HBI. Side effects were predictable. HBI is effective salvage therapy in resistant myeloma.

### Bone marrow transplantation for acute myeloid leukaemia

C.R. Hamilton, A. Barrett & R. Powles

*The Royal Marsden Hospital, Sutton, Surrey, UK.*

Between August 1977 and March 1986, 237 patients with acute myeloid leukaemia (AML) received an allogeneic bone marrow transplant as part of their management at the Royal

Marsden Hospital (RMH). The actuarial 5 year survival rate post transplantation is 35%.

Results of bone marrow transplantation

| No. | Donor marrow | Conditioning | AML status | Actuarial surv. rate |
|---|---|---|---|---|
| 117 | matched | chemo/TBI | 1st remission | 46% at 5 yrs |
| 42 | matched | chemo/TBI | not 1st remission | 24% at 5 yrs |
| 62 | mismatch | various | various | 18% at 5 yrs |
| 16 | matched | chemo | various | 31% at 2 yrs |
| 237 | | | | |

Since 1978, 62 patients presenting at the RMH with AML have achieved complete remission and have had no transplant procedure performed and their actuarial 5 year survival rate is 22%.

An analysis of results, prognostic factors and the input of conditioning regimen on leukaemia control and morbidity will be presented as well as views on future management.

### A randomised trial of two chemotherapy regimens in osteosarcoma

R.L. Souhami

*On behalf of the European Osteosarcoma Intergroup (MRC/UKCCSG/SIOP/EORTC/GETO) and MRC Bone Sarcoma Working Party, UK.*

From August 1983 to November 1986, 307 patients with osteosarcoma were entered into a randomised prospective comparison of two chemotherapy regimens. Treatment A consisted of 6 three-weekly cycles of doxorubicin $25\,mg\,m^{-2}$ days 1–3 and cisplatin $120\,mg\,m^{-2}$ day 1. Treatment B was 4 cycles of the same doxorubicin/cisplatin treatment alternating with methotrexate $8\,g\,m^{-2}$. Fifteen patients were ineligible. Of 292 eligible patients, 63 had metastases at presentation and in 229 patients chemotherapy was given as an adjuvant with surgery planned for 9 weeks after the start of drug treatment. Thirty-five percent of patients had amputation, the rest having more conservative surgery (50% endoprosthetic replacement). Haematological toxicity was the same in both arms with neutropenia more prominent than thrombocytopenia. Nausea and alopecia were almost invariable but equal in the two arms. Raised transaminases occurred in 35 patients on treatment B, but none on regimen A. At a median follow-up of 23 months median survival has not yet been reached. In the adjuvant patients, disease-free survival is better for the 2 drug arm ($P=0.055$) with no significant difference in survival. These data imply that a short 2 drug chemotherapy programme gives results comparable to the standard multidrug (T10) regimens widely used and this is the basis of the new randomised study started in October 1986.

### The use of radiotherapy in the management of new and recurrent cases of soft tissue sarcomas of the limbs and limb girdles

M.H. Robinson & C.L. Harmer

*Sarcoma Unit, The Royal Marsden Hospital, London, UK.*

Since 1981, 93 patients with limb or limb girdle soft tissue sarcomas have received radiotherapy at the Royal Marsden Hospital as part of their limb conservation treatment. Sixty-eight were referred as new cases and 25 as recurrences with no prior radiotherapy. Radiotherapy was given pre-operatively in 15 new (N) and 16 recurrent (R) cases; post-operatively in 45N and 15R; both pre- and post-operatively in 6N and 2R and palliatively (with no definitive surgery) in 4. MFH, liposarcoma and synovial sarcomas predominated in this series. Tumour sites included lower limb proximal – 53 cases, distal 19; upper limb proximal 9, distal 12. Surgical clearance was graded according to Enneking and in new and recurrent groups was intracapsular in 11 and 3 respectively; marginal in 29N and 11R; wide in 8N and 8R; radical in 6N and 1R and uncertain or not undertaken in 14N and 2R. Sixty-four percent of new sarcomas and 52% of local recurrences were high grade.

Radiotherapy regimes varied between 40 Gy given in 6 weekly fractions in 15 cases to 75 Gy given in 60 twice daily fractions in 4 cases. Forty-one patients received 60 Gy in 30 daily fractions in 6 weeks. Median follow-up was 2 years. Actuarial 3 year survival (80% *vs.* 65%) and local control rates (80% *vs.* 68%) were not significantly different for new and recurrent cases (logrank) and no significant difference in control rates for those given pre- or post-operative radiotherapy. All recurrences were in field except 2 which were marginal. The role of close surgical and radiotherapy margins, radiation dose, tumour size and grade in failure of local control will be discussed.

### Tumour and Normal Tissue Biology II

### Tumour Angiogenesis: An omental bioassay

R.J.L. Williams

*The Institute of Cancer Research and Royal Marsden Hospital, London, UK.*

The formation of new blood vessels is important to the growth of most solid cancers. However, histological sections reveal little about the mechanisms of neovascularisation and experimental models have been limited by the lack of a precise and reliable endothelial cell marker. Evaluation of tumour induced vascular growth is often qualitative and subjective. A new method of studying angiogenesis is described.

In SWR mice, a single intraperitoneal injection of $10^7$ Landschutz ascites cells was used to stimulate microvascular proliferation in the gastric mesentery or omental (Landschutz cells were first irradiated to prevent tumour formation). Capillary endothelial cells and their projections were stained using a peroxidase conjugate of the plant lectin *Dolichos biflorus* agglutinin.

Observed in whole mounts 30 microns in thickness, blind ending processes or 'sprouts' were seen to arise from omental capillaries. After tumour cell stimulation, the mean number of capillary sprouts per omentum increased from 36.7 to 240.3 ($P=0.0002$). Concurrent heparin administration produced an exaggerated sprout response (mean 396.9, $P=0.0288$), while vascular sprouts were almost abolished by i.p. hydrocortisone (mean 12.2, $P<0.0001$).

This 'bioassay' provides an easy and reproducible means of distinguishing and measuring early vascular growth. The technique can be used to investigate potential promoters and inhibitors of tumour angiogenesis, and gives a new insight into the morphological changes involved.

### The effect of fractionation of light treatment on necrosis and vascular function of normal skin following photodynamic therapy.

K. Benstead, R.D. James & J.V. Moore

*Paterson Institute for Cancer Research, Christie Hospital and Holt Radium Institute, Manchester M20 9BX, UK.*

Photodynamic therapy is being increasingly investigated as a treatment modality in clinical trials. Many of these studies have used fractionated light treatment although there is little data available on the effect of this on normal tissue.

Sparing of normal tissue, mouse tail skin, by fractionation of light treatment has been demonstrated in BDF1 mice injected with 2 mg tetrasodium-meso-tetra (4-sulphophenyl) porphine dodecahydrate i.v. When the time between 2 fractions of $67.5\,J\,cm^{-2}$ and $90\,J\,cm^{-2}$ was increased to 2 and 4 days respectively the incidence of necrosis fell to that expected after a single fraction. Blood flow in the tail skin 5 days after the second light fraction, as measured by the clearance of an intradermally injected solution of $^{133}$xenon in 0.9% saline, returned to control values when the time between 2 fractions was 2 days, with $67.5\,J\,cm^{-2}$ fractions and 3 days with $90\,J\,cm^{-2}$ fractions.

The time course of recovery of normal mouse tail skin from photodynamic therapy as shown by these split dose experiments was found to be similar to the time course for the recovery of blood flow following a single light treatment.

Treatment with multiple fractions of $67.5\,J\,cm^{-2}$ at intervals of 4 days allowed a dose of $270\,J\,cm^{-2}$ to be given without producing necrosis although this is the $ED_{50}$ dose when given as a single fraction.

### $^{31}$P magnetic resonance spectroscopy in monitoring the effects of photon and neutron irradiation on HT29 human adenocarcinoma cells *in vitro*

S. Myint[1], H.M. Warenius[1], J. West-Jordan[2], R. White[1], R. Abraham[2] & R.H.T. Edwards[3]

[1]*Department of Radiation Oncology,* [2]*School of Chemistry and* [3]*Department of Medicine, University of Liverpool, Liverpool, Merseyside, UK.*

The application of $^{31}$P NMR to the study of living cells has provided a non-invasive monitoring of cell energetics and offers a method for observing the effects of radiation on metabolism of tumours.

$^{31}$P NMR can be used to determine the relative concentration of ATP, creatinine phosphate (PCr), inorganic phosphate (Pi) and several other phosphorated compounds such as (PME, PDE, DPDE). In addition, tissue pH can be calculated from Pi chemical shift.

We have used $^{31}$P NMR to study the metabolic changes related to hypoxia and have characterised the spectral changes occurring during recovery from hypoxia. There was a fall in ATP levels with concomitant increase in inorganic phosphate levels during hypoxia and these changes are almost completely reversed during recovery from hypoxia.

Following irradiation with photons and neutrons, the pattern of recovery from hypoxia differs. The irradiated HT29 cells showed an increase in Pi/ATP, PM/ATP and also the value of Pi, with maximum effect occurring after 3 h. These changes are more marked following neutron irradiation and the increased Pi/ATP ratio persists after recovery. The significance of these findings requires further investigation. It is of interest, however, that $^{31}$P NMR enables effects of protons and neutrons to be evaluated in intact living cells in non-DNA sites.

### Non-invasive ablation of liver tumours using focussed ultrasound

G. ter Haar[1], D. Sinnett[2] & J. Dodds[3]

[1]*Department of Physics,* [2]*Department of Surgery,* [3]*Haddow Laboratories, Sutton and Institute of Cancer Research, Sutton, UK.*

Ultrasonic energy may be focussed in such a way that it can be concentrated within a small volume of tissue. If sufficiently high power levels are used, tissue lying within the focal region may be selectively destroyed at depth, leaving surrounding tissue undamaged. The primary mechanism for damage is heating, temperatures in the region of 80°C being achieved within 10–15 sec. The region of damaged tissue can be visualized on a diagnostic ultrasound image, as it is created. This technique potentially provides a method for the non-invasive treatment of liver metastases, without damage to intervening tissues, while allowing 'real-time' monitoring of the extent of tissue damage.

The effect of focussed ultrasound lesions on normal rat liver has been investigated, and the capacity of the liver to accommodate and replace the damaged volume has been studied. After 10 weeks there is no sign of the ultrasonically produced lesion.

### Louise Buchanan Memorial Lecture

### The biology of melanoma: Implications for early detection

R.M. MacKie

*Department of Dermatology, University of Glasgow, Glasgow, UK.*

The incidence of cutaneous malignant melanoma is currently rising rapidly and a high proportion of affected individuals are otherwise healthy young adults. Although both the World Health Organisation and the European Organisation for Research and Treatment of Cancer run trials on melanoma chemotherapy, there is no proven effective adjuvant or therapeutic regime yet available.

It is possible to prognosticate with remarkable accuracy for melanoma patients at the time of presentation with their primary tumour as five year survival is directly and inversely related to the tumour thickness. It thus follows that encouraging early recognition and prompt surgical therapy is currently likely to be the most effective method of reducing or at least controlling mortality. Changing approaches to surgical management will also be discussed.

### Proffered papers

### New Approaches to Diagnosis and Treatment

### Use of interleukin 2 in human cancer: *In vitro* and *in vivo* studies and potential for augmentation with monoclonal antibodies

A.C. Dalgleish[1], M. Malkovsky[1], C. Bello[2] & P. Sondel[2]

[1]*MRC Clinical Research Centre, Harrow, Middlesex, UK; and* [2]*University of Wisconsin, Madison, USA.*

Previous results have shown that human peripheral blood leukocytes (PBL) expanded in interleukin 2 (IL-2): i.e., lymphokine activated killer (LAK) cells, can destroy various normal and tumour target cells. The induction of this non-specific cytotoxic activity is independent of *de novo* DNA synthesis and cannot be further potentiated by gamma interferon. We tested for the presence in clinical patients, for LAK activity before and after IL-2 therapy. We also looked for potential inhibition and augmentation of LAK with monoclonal antibodies (MAb) *in vitro*.

We tested the effect of monoclonal antibodies to LFA-1, CD2, CD3, CD4, CD8 and HLA molecules on killing mediated by LAK cells on peripheral blood lymphocytes, K562 and Daudi cells. LAK activity from clinical patients was tested in the absence of MAb.

Anti-LFA-1 strongly inhibited the killing of normal PBL and to a lesser extent the killing of tumour cells. Anti-CD2, CD4, CD8 and HLA class I did not inhibit non-specific killing. In contrast, anti-CD3 potentiated the killing of peripheral blood lymphocytes, K562 and Daudi cells.

### Regional perfusion of antibody/isotope conjugate can increase tumour uptake

T. Hennigan, R. Carpenter, J. Matthews, A. Chidlow, B. Pedley, R. Begent & T. Allen-Mersh

*Departments of Surgery and Medical Oncology, Charing Cross and Westminster Medical School, London, UK.*

The value of antibody/isotope conjugate (AIC) in delivering a lethal radiation dose to solid tumours is limited by poor differential uptake of tumour when compared to normal tissue. We have assessed whether regional perfusion of AIC increases tumour uptake in a novel animal model.

LS174T CEA-producing colon cancer cells were injected subcutaneously to produce thigh tumours in nude rats. Three weeks after inoculation, once solid tumours (mean weight 4.6 g) were evident, the femoral artery was cannulated and anti-CEA monoclonal antibody labelled with 70–100 $\mu$Ci $^{131}$I was infused over 1.5 h. The femoral artery was ligated at completion of perfusion. In further animals, the femoral artery was ligated but AIC was administered intravenously into the tail vein. Animals were sacrificed at 48 h and the tumours were removed and counted.

After correction for difference in antibody dose, mean uptake of regionally administered AIC ($8.8\times10^{-2}\,\mu$Ci g$^{-1}$ tumour) was significantly greater than that of systemically administered AIC ($5.9\times10^{-2}\,\mu$Ci g$^{-1}$ tumour, Mann Whitney test, $P<0.05$). Both systemic and regionally perfused tumours showed significantly higher uptake than normal liver, mean $3.7\times10^{-2}\,\mu$Ci g$^{-1}$ liver (Mann Whitney test, $P<0.04$ and $P<0.02$ respectively).

Tumour uptake of AIC appears to be enhanced by regional administration which might be of use in the management of metastatic colorectal cancer.

### Limitations of antibody targeting: A model showing higher uptake ratios using a smaller, protein-bound molecule – Application to MIBG

G.D. Thomas, A.R. Bradwell, M. Chappell, P.W. Dykes, K. Godfrey & J.R.M. Ellis

*University of Birmingham, Birmingham, U.K.*

Poor tumour uptake of radiolabelled antibodies has prevented their widespread application in diagnosis or therapy. We have constructed a compartmental model to assess the relative importance of the major limiting factors, namely: (1) molecular size; (2) affinity of tumour binding and (3) binding to normal tissue. The model also assesses the feasibility of using a smaller molecule with a carrier protein delivery system to prevent urinary loss. Amounts bound and free in plasma and ECF as well as on tumour and normal tissue receptors are calculated over time following injection. Differential rate equations with widely varying rate constants are solved using FACSIMILE which avoids the inaccuracy of steady-state assumptions previously necessary in such models. It is also possible to simulate clearance of protein-bound molecules from the system (by injecting a competitor for the protein binding site) when tumour counts have reached a maximum. Uptake ratios of over 100 can result. Support for the binding-protein concept comes from studies of MIBG which has small dimensions and achieves high tumour concentrations. We have shown that it is bound to plasma proteins *in vitro* and suggest the possibility of improving uptake ratios based on this model.

### CEA expression and radioimmunodetection of pancreatic tumours and cholangiocarcinoma

A. Jewkes, F. Macdonald, W.H. Allum & R. Downing

*Surgical Immunology Unit and Department of Surgery, University of Birmingham and Queen Elizabeth Hospital, Birmingham, U.K.*

Radioimmunodetection using antibodies to carcinoembryonic antigen (CEA) is effective in localising gastrointestinal adenocarcinoma. Its usefulness may be of limited value unless the information gained is unobtainable by other less labour intensive diagnostic procedures. Pancreatic carcinoma and cholangiocarcinoma are both difficult to differentiate from their non-neoplastic counterparts, chronic pancreatitis and sclerosising cholangitis, using existing diagnostic methods. Antigen expression of both these tumours was studied using an immunohistochemical technique with a monoclonal antibody to CEA. Seventy-seven percent of pancreatic cancers were positive, as were 80% of cholangiocarcinomas. Expression of CEA was markedly reduced in terms of both intensity of staining and the number of positive cells, in cases of chronic pancreatitis and sclerosing cholangitis.

Patients with one of these conditions proven histologically, were given 200 $\mu$g monoclonal anti-CEA antibody labelled with iodine 131. Subjects are imaged at 24 and 48 h and results compared with laparotomy findings and/or CT scanning. All 10 pancreatic tumours were clearly imaged using this technique as were all bile duct tumours. There was however significant uptake of antibody in many cases of chronic pancreatitis and some cases of sclerosing cholangitis. Despite the encouraging results of immunohistochemistry studies these antibodies are unlikely to be able to differentiate between benign and malignant pancreatic or biliary conditions. The results suggest, however, that antibody targetted therapy may be possible in these conditions.

### Long acting somatostatin analogue in malignant carcinoid syndrome

M. Ellis[1], S. Levi[3], E. Leung[2], E. Adam[2], J. Calam[3] & H.J.F. Hodgson

*Departments of [1]Oncology, [2]Radiology and [3]Medicine, RPMS, Hammersmith Hospital, London, UK.*

Long acting somatostatin has been advocated as treatment for carcinoid syndrome. We report results in 10 patients with gastrointestinal carcinoid with hepatic metastases, resistant to conventional blocking drugs, treated with long acting somatostatin (sandostatin) in doses of 50 to 250 $\mu$g tds by self-administered subcutaneoius injection. In all patients there was effective and rapid sympton relief, of diarrhoea, flushing, palpitations, and wheezing when present. The effect of therapy on release of serotonin from tumour was unpredictable, both increased and decreased 5 HIAA levels being found, suggesting that much of the action of somatostatin is peripheral. Serial observations of tumour size in this group of patients did not show convincing evidence of a direct anti-tumour action, with growth in tumour deposits over six months of therapy being recorded on sequential CT scans, or obvious development of extrahepatic tumour masses occurring. Side effects were limited to occasional local pain at the injection site, and biochemical evidence of glucose intolerance. Currently long acting somatostatin is an effective means of preventing symptoms in carcinoid syndrome, but an effect on survival would not be predicted.

### Interstitial photodynamic therapy in man

D. Gibson[1], D.V. Ash[1], J.W. Feather[2], I. Driver[2], P. King[2] & S.B. Brown[3]

[1]*Departments of Radiotherapy,* [2]*Medical Physics and* [3]*Biochemistry, University of Leeds, Leeds, UK.*

A main limitation of photodynamic therapy (PDT) in treatment of cancer is the limited penetration of light in tissue. One possible way of increasing the range of tumours which can be treated is to use optical fibres inserted into the tumour to deliver light interstitially.

Four patients with 23 sites of superficial recurrent tumour have been treated with photofrin II ($1.5\,mg\,kg^{-1}$ i.v.) and, 72 h later, interstitial irradiation, using a $200\,\mu m$ optical fibre to deliver 50–300 J of 630 nm light from a copper vapour/dye laser. Also, 3 of the patients had a total of 6 lesions treated with $50–75\,J\,cm^{-2}$ of superficial light. All except two of the sites treated with interstitial PDT showed partial tumour regression and none of the sites developed skin necrosis. Three sites treated with superficial PDT showed complete tumour regression, and 3 sites showed partial regression. Skin necrosis occurred at all 3 sites showing a complete tumour response.

Our previous studies of superficial PDT showed a complete tumour response rate of 83%, with 1.5 or $2.0\,mg\,kg^{-1}$ of photofrin II and $50–75\,J\,cm^{-2}$ of light, which was closely paralleled by skin necrosis. Comparing these results with the new data presented suggests that interstitial PDT, as given in this study, is less effective than superficial PDT. This may be due to insufficient doses of light or alteration in light absorption in tumour when light is applied interstitially. These factors require further investigation.

### Posters

### Tumour and Normal Tissue Biology

### Lethal mutations and their effect on the application of radiobiological results to radiotherapy

C.B. Seymour[1] & C. Mothersill[1,2]

[1]*Saint Luke's Hospital and* [2]*Dublin Institute of Technology, Dublin, Ireland.*

Much of the theory on which radiotherapy is based comes from radiobiological studies of cells in culture, using the Puck & Marcus clonogenic assay. However, by determining the clonogenic survival of cells within survivor clones, we have shown that there is a dose-dependent reduction in the survival of these cells. When data obtained from conventional Puck & Marcus clonogenic assays are corrected for these residual cell deaths, which we have termed lethal mutations, the shoulder to the survival curve is lost. This association between the occurrence of a shouldered survival curve and existence within the cell population of lethally mutated or 'doomed' fraction was confirmed by examining repair deficient cells which normally exhibit no shoulder and by exposing cells to neutron irradiation, after which again no shoulder is usually observed. In neither case were lethal mutations evident.

Of major interest in radiotherapy are the results following split dose irradiation since most patients are treated using fractionated regimes. However, these suggest that, while the single dose survival curve shoulder is eliminated, the split dose shoulder remains. This finding is confirmed by the construction of Elkind recovery curves which show that 'early' recovery taking place 0–2 h post-irradiation mainly results in cells which die later (lethally mutated cells), while recovery taking place over 2–24 h results in cells which survive in the long term. The results have important implications for the construction of models relevant to radiotherapy treatment planning and may also help to explain late effects.

### *In vitro* cell response measured in a dye exclusion and MTT assay

J. Hanson[1], J.L. Moore[1], D.P. Bentley[2] & A. Bean[1]

[1]*Department of Radiation Sciences, Velindre Hospital, Whitechurch, Cardiff; and* [2]*Department of Haematology, Llandough Hospital, Cardiff, UK.*

A dye exclusion assay based on the ability of fixed 'live' cells to exclude alcian blue (Yip & Auersperg, *In vitro,* **7,** 323 (1972)) has been evaluated as an assay to measure cell response following chemotherapy. Initially cell lines (Molt 4 and Daudi) were used. After exposure, cells were cultured for 4 days before fixation. After staining with alcian blue (including duck RBCs as standard), cytospin slide preparations were made and finally counter-stained with Giemsa. Dose response data was obtained following radiation or drug exposure. To determine the application of this assay we have also now assayed 13 samples from 7 patients with CLL and have obtained dose responses in most cases when tested against chlorambucil and novantrone. The CLL samples were also assessed in the MTT assay (Twentyman & Luscombe, *Br. J. Cancer,* **56,** 279 (1987)). This assay is based on the metabolism by viable cells of a tetrazolium salt producing formazan crystals which are solubilised and the resultant absorbance measured. Of the CLL samples, 9 of 13 gave significant formazan production where dose responses were detectable. Our results suggest these assays may have clinical use and we are currently undertaking a larger study of CLL patients.

### Radiosensitive human tumour cells are not recovery deficient

J.H. Peacock, A.M. Cassoni, T.J. McMillan & G.G. Steel

*The Institute of Cancer Research, Sutton, Surrey, UK.*

Cellular recovery from radiation damage has often been demonstrated using the split-dose experiment and the results interpreted using the multi-target model of radiation effect. This model predicts that once the fraction dose used in a split-dose experiment is off the shoulder of the survival curve, then recovery will remain constant. In contrast the more widely used linear-quadratic model (LQ) predicts that recovery will increase steeply as an exponential function of dose squared, at a rate determined by the B-component of the LQ equation.

To test these predictions we have performed split-dose experiments on four human tumour lines of widely differing radiosensitivity. The pattern of the results follows that predicted by the LQ model suggesting that the B-component of that model may be used as a measure of cellular recovery.

The most surprising result, however, is that our data shows that recovery capacity increases with increasing radiosensitivity. This is opposite of what is expected since it is usually accepted that cellular radioresistance is at least partly due to increased cellular recovery capacity. In addition, the data throw doubt on the common assumption that cells which have very steep radiation survival curves are recovery deficient.

### Cellular recovery in sensitive and resistant lines of the L1578Y murine lymphoma

T.J. McMillan, J.J. Eady, J.H. Peacock & G.G. Steel

*Radiotherapy Research Unit, The Institute of Cancer Research, Sutton, Surrey, UK.*

Lines of the L5178Y lymphoma which vary widely in their sensitivity to ionizing radiation have been used in the past to examine the molecular basis of radiosensitivity. Two such lines, LY-S and LY-R which differ in their sensitivity by a factor of 5, have been investigated here to establish their recovery proficiency and thus assess their suitability for studies of DNA repair. Two commonly used assays of recovery capacity have been used. The extent of dose-rate sparing is measured by comparing the survival of cells after acute and low dose rates. Split dose experiments assay the recovery of the cells when two halves of an acute dose are separated in time.

Both LY-R and LY-S show a decrease in sensitivity as the dose rate is decreased but the extent of this suggests that there is little difference in the recovery proficiency of the two lines. In contrast the recovery ratio (RR) in split dose experiments at 0.01 survival suggest that the more resistant line (LY-R) has a recovery capacity which is twice that of the sensitive line (LY-S).

This inconsistency is due to the underlying assumption of a multitarget model in using only one level of cell kill in split dose experiments. As an alternative the linear-quadratic (L-Q) model infers that RR will be a variable for each cell line which depends on the dose and on the B coefficient. This B value may thus be a better measure of recovery capacity and if used for these lines we conclude that LY-S is in fact more recovery proficient than LY-R. These results emphasize the difficulties involved in measuring recovery in cells and suggest that recovery proficiency need not decrease with increased sensitivity.

### Flavone acetic acid (FAA) (LM975, NSC 347512) induces rapid massive necrosis in a murine transplantable adenocarcinoma (MAC 26)

C.V. Duke, J.A. Double & M.C. Bibby

*Introduced by S.M. Crawford, Clinical Oncology Unit, University of Bradford, Bradford, UK.*

FAA is a novel anti-cancer agent undergoing clinical evaluation in Europe and the USA. It is highly active against a series of transplantable s.c. solid colon tumours in mice (Bibby *et al.*, *Br. J. Cancer*, **55**, 159 (1987)). The response of MAC 26 was particularly impressive as this tumour is refractory to standard cytotoxic therapy. Previous studies also indicated that these responses did not correlate with direct cell kill as demonstrated by *in vitro* cytotoxicity and that the establishment of a vasculature appeared to be important.

In an attempt to elucidate the mechanisms of action of FAA we have followed histological changes and effects on blood flow in the well differentiated slow growing cystic adenocarcinoma MAC 26. Tumour blood volume was measured using an Evan's blue perfusion technique (Harada *et al.*, *J. Pharm. Pharmac.*, **23**, 218 (1971)). A single i.p. therapeutic dose (90% tumour volume inhibition) of FAA ($200 \mathrm{mg\,kg^{-1}}$) produced a 60% reduction in tumour blood volume first demonstrated 4 h after treatment and which persists for at least 24 h. Parallel histology studies demonstrated massive tumour necrosis at 24 h with necrotic changes being seen as early as 2 h after treatment. An equivalent therapeutic dose of local irradiation does not produce these histological effects.

These observations substantiate our earlier suggestions that tumour vasculature may be involved in the dramatic response of s.c. tumours to FAA. This may provide an explanation for the lack of response seen in the clinic where the biology of systemic disease may be very different from that of subcutaneous tumours in mice.

### Quantification of CEA synthesis and secretion by different cultured human tumour cell-lines

J.R.M. Ellis, A.R. Bradwell, P.W. Dykes & G.D. Thomas

*Immuno Diagnostic Research Laboratory, The Medical School, University of Birmingham, Birmingham B15 2TJ, UK.*

Carcinoembryonic antigen (CEA) has been described as an oncofetal protein, being expressed in greater concentrations on embryonic and malignant tissues compared with normal adult cells. Antibodies raised against this protein have been applied to radioimmunodetection (RAID) studies in both animals and humans, but with limited success to date. Relative rates of CEA synthesis and secretion by normal and malignant cells are of major importance and their accurate measurement has not previously been possible. To achieve this, CEA-secreting human tumour cell lines were cultured in medium containing tritiated L-leucine to produce labelled CEA which was then isolated from the cell supernatant by 2-D immunoelectrophoresis and precipitated against anti-CEA. A multi-wire proportional camera (MWPC) was then used to quantitate the activity in the protein precipitate and from this value the amount of CEA produced was calculated. Preliminary results with three tumour cell lines show that CEA levels of the order of $10^{-2}$ ng are detectable from supernatants of $10^6$ cells cultured for 72 h. Future work will enable calculation of CEA production for these cell lines and eventually for malignant and normal tissues removed during surgery and maintained in short term culture. Once CEA has been assessed the technique could be applied to other tumour associated proteins to evaluate their potential use for RAID.

### Anti-tumour activity and bone marrow toxicity of novel chloroethylnitrosoureas

A.M. Matthew[1], C.G.Boynton[2], M.C. Bibby[1], J.A. Double[1] & S.M. Crawford[1]

[1]*Clinical Oncology Unit and* [2]*School of Biomedical Sciences, University of Bradford, Bradford, UK.*

The development of analogues of chloroethylnitrosoureas with reduced toxicity has been a major goal. Ehresmann *et al.* (*Arch. Pharm.*, **317**, 481 (1984)) have synthesized a series of compounds with the chloroethylnitrosocarbamoyl (CNC) residue linked to an amino acid or its derivative. CNC-alanylalanine (E94) and CNC-glycinemethylamide (E126) were examined, using the clinically active, taurine based nitrosourea (TCNU) as a positive control. Anti-tumour activity was assessed against three lines of differing growth characteristics and morphology from the mouse adenocarcinoma of the colon (MAC) series. At equivalent CNC concentrations ($0.1 \mathrm{mM\,kg^{-1}}$) TCNU and E126 produced over 90% tumour weight inhibition of MAC 13 and 80% tumour volume inhibition of the relatively insensitive solid tumour, MAC 26. E94 was inactive against MAC 26 despite a higher level of CNC ($0.17 \mathrm{mM\,kg^{-1}}$), but exhibited improved activity against the ascitic tumour, MAC 15A, with a four-fold increase in median survival time and two cures. Marrow toxicity was assessed by a spleen colony forming unit assay at anti-neoplastic dose levels. TCNU and E126 showed marked marrow toxicity with no detectable surface colonies while E94 showed no significant myelosuppression with a survival fraction of 0.98; marrow toxicity was only demonstrated at levels in excess of the maximum tolerated dose. These preliminary results suggest the importance of the carrier groups in determining the activity and toxicity of the chloroethylnitrosoureas. The demonstration of anti-tumour activity at non-myelosuppressive dose levels with CNC-alanylalanine is encouraging for the development of nitrosoureas with improved therapeutic indices.

### Differential sensitivity of malignant cells to saturated 18-carbon fatty acids *in vitro*

B. Fermor[1], N.A. Habib[1], J.R.W. Masters[2], J. Miller[1], C.B. Wood[3] & R.C.N. Williamson[3]

[1]*Department of Surgery, Bristol Royal Infirmary;* [2]*Institute of Urology, London; and* [3]*Department of Surgery, Royal Postgraduate Medical School, London, UK.*

The membranes of malignant cells showed increased oleic acid in relation to stearic acid content in comparison with their normal counterparts (Wood *et al.*, *Eur. J. Oncol.*, **11**, 347 (1985)). Saturated stearic and its derivative dihydroxy-stearic (DHSA), and monounsaturated oleic acids were tested for their ability to inhibit colony formation of 5 malignant cell lines, 1 normal urothelium and 1 normal fibroblasts *in vitro*. Stearic acid was up to 5 times more cytotoxic than oleic acid. Four out of five tumour cell lines were more sensitive to stearic acid than the normal fibroblasts ($<0.001<P<0.05$). The three tumour cell lines tested showed statistically significant ($<0.001<P<0.006$) differential sensitivity to DHSA than to the normal urothelium or normal fibroblasts.

| *Cell line* | | *DHSA - I.C. 50 μg ml⁻¹ (mean ± s.e.m.)* |
|---|---|---|
| Colon tumour | HT29 | 22.44 ± 1.2 |
| Bladder tumour | RT112 | 11.20 ± 1.2 |
| Testicular tumour | 833K | 22.24 ± 0.4 |
| Normal urothelium | HU609 | 34.10 ± 0.5 |
| Normal fibroblasts | HFL | 36.31 ± 1.1 |

Our data suggest saturated 18 carbon fatty acids to be more cytotoxic to tumour cells than monounsaturated 18 carbon fatty acids and that this may be related to the higher proportion of oleic acid in the tumour cell membranes.

### Reactions of carboplatin with chloride ions – pharmaceutical and clinical implications

M. Allsopp, G.J. Sewell, M. Northcott & C.G. Rowland

*Postgraduate Medical School, University of Exeter, Exeter, UK.*

Phase I/II studies on the continuous infusion of carboplatin with synchronous radiotherapy against solid tumours are in progress at Exeter. For biological activity, carboplatin reacts non-enzymically with water. We have shown carboplatin to also undergo nucleophilic substitution with $Cl^-$ ions. These reactions are important in continuous (5 day) infusions since long-term drug stability is essential, particularly in infusions containing carboplatin and $Cl^-$ ions, the latter arising as a formulation excipient or drug counter-ion in multiple agent regimes. The plasma $Cl^-$ concentration is 0.1 M so the reaction is also significant *in vivo*. Kinetic studies on carboplatin degradation were carried out at elevated temperatures in the presence of 0, 0.1, 0.25 and 0.4 M M $Cl^-$.

Degradation was 1st order and the observed rate constant (Kobs) comprised two components ($Kobs = K_1 + K_2 [Cl^-]$), where $K_1$ and $K_2$ are hydrolytic and $[Cl^-]$ dependent rate constants respectively. Values of $K_1$ and $K_2$ were determined at pharmaceutically and clinically relevant temperatures and from these the times for 5% ($T_{95}$) and 50% ($T\frac{1}{2}$) degradation were calculated. At 25°C values of $9.74 \times 10^{-4}$ and $3.14 \times 10^{-3} [Cl^-].h^{-1}$ were obtained for $K_1$ and $K_2$ respectively, giving $T_{95}$ values for carboplatin of 52.7 h in water and 29.2 h in 0.9% saline. Carboplatin should not be reconstituted with saline for continuous infusion. At 37°C with 0.1 M $Cl^-$ the carboplatin $T\frac{1}{2}$ was 334 h and 5 putative plasma intermediates with possible biological/ radiosensitisation activity are proposed. The *in vitro* plasma $T\frac{1}{2}$ was approx. 30 h suggesting that carboplatin reacts with other plasma components in addition to $Cl^-$ ions.

### Technetium-99m HMPAO SPECT and blood flow patterns in human lung tumours

N.P. Rowell, V.R. McCready, D. Tait, M.A. Flower, B. Cronin & A. Horwich

*MRC Radiobiology Unit, Harwell, and Radiotherapy Unit, The Royal Marsden Hospital, Sutton, Surrey, UK.*

In order to assess the blood flow patterns through human lung tumours, 20 patients received 400–750 MBq $^{99m}Tc$-HMPAO intravenously 10 min prior to single photon emission computed tomography (SPECT). Ratios of uptake in the whole tumour relative to normal lung ranged from 0.35 to 1.53 (mean 1.01) with 8 tumours showing less uptake than normal lung and 10 showing greater uptake. In one patient the tumour was not distinguishable from surrounding lung and in another a large pleural effusion prevented evaluation. Tumour: lung ratios for central tumour regions ranged from zero to 1.83 (mean 0.80) with 13 showing lower uptake than normal lung and 5 showing greater uptake. Duplicate scans were performed in 8 patients demonstrating satisfactory reproducibility. This technique provides a simple and reproducible method for the assessment of tumour blood flow

### Development of a technique for optimising cancer therapy using a human epithelial model system

C. Mothersill[1,2], C.B. Seymour[1], A. Cusack[1], T.P. Hennessy[3] & M. Moriarty[1]

[1]*Saint Luke's Hospital,* [2]*Dublin Institute of Technology and* [3]*St. James's Hospital, Dublin, Eire.*

The optimisation of cancer therapy regimes is mainly done by retrospective analyses of clinical trial results. The idea of experimentally optimising treatments prior to use on particular tumours or patients is not new but experimental models are largely irrelevant or too difficult to be used widely.

Our group has developed a quick, easy and widely applicable tissue culture model which is giving promising results in this area. Explants from a wide variety of normal human tissues (genitourinary, gastrointestinal tract, glandular) and their respective tumours give rise over four weeks to largely epithelial outgrowths when cultured in a basal medium with serum, hydrocortisone, insulin and specific hormone supplements. The outgrowths can be treated with appropriate combinations of radio- and/or chemotherapy at any time after explanation, using any sequence or dose required. Results are analysed as reduction in growth area relative to the untreated control. Growth is confirmed using autoradiography or K1 67 antigen (Dako) and the epithelial nature of the culture is confirmed using cytokeratin immunocytochemistry.

Results using bleomycin + radiation on oesophageal normal and tumour tissue or cisplatin + radiation on bladder normal and tumour tissue suggest tumour cytocidal results may be optimised by the concurrent application of a clinically equivalent chemotherapy dose with a small single radiation dose. This reduced tumour cell growth to zero over a four week period after treatment, while sparing the surrounding normal tissue. Higher drug or radiation doses produced equal cytotoxicity in normal and tumour explants.

The technique has potential for predictive tumour therapy and clinical trials are in the process of being set up.

### New Approaches to Diagnosis and Treatment

### $^{131}$I-MIBG targetted radiotherapy in Yorkshire

A.S. Bulman, R.E. Taylor, M. Sheppard & I. Lewis

*Departments of Radiotherapy, Medical Physics and Paediatric Oncology, Cookridge Hospital, Leeds, UK.*

Meta-iodobenzylguanidine (MIBG) is a synthetic molecule taken up by adrenergic neuro transmitter vesicles. Labelled with $^{131}$Iodine in 18.5 MBq doses it is used for scintigraphic imaging of neurectodermal tissues and tumours. We report 4 cases in whom therapeutic doses were used as 'targeted radiotherapy'.

Patient 1, aged 11, had metastatic nodal neuroblastoma 4 years after primary treatment. Two patients had neuroblastoma persisting despite conventional treatment (patient 2, aged 4, in marrow, and patient 3, aged 5, in primary abdominal site and bone). Patient 4, aged 15, had inoperable right adrenal paraganglioma with hypertension.

After thyroid blockade 74 MBq tracer doses were used to estimate therapeutic doses, which were given as 45 min i.v. infusions of 3.7, 3.3, 2.8 and 5.6 GBq respectively, requiring admission to a designated side ward for radiation protection purposes for one week. Whole body scan and static scintigrams were obtained from days 3–7 and weekly for four weeks enabling whole body and region of interest clearance curves to be plotted. Nausea was seen in patients 3 and 4 whose primary sites were adjacent to liver, but no major treatment related side effects were seen. Patient 1 showed tumour size reduction of more than 50% for five weeks, 2 and 4 showed no response, 3 advancing disease.

Work is continuing on the biological behaviour of $^{131}$I-MIBG in children to allow increased tumour doses to be given without excessive whole body dose. A UKCCSG Study is now open for children with advanced neuroblastoma.

### Interstitial laser hyperthermia: A potential new treatment for tumours of the liver and pancreas

A.C. Steger[1], S.G. Brown[1], W.R. Lees[2] & the late C.G. Clark

*Departments of [1]Surgery and [2]Radiology, University College and Middlesex Hospital, London, UK.*

Few cancers of the liver and pancreas are suitable for either curative resection or radiotherapy or chemotherapy, the majority needing palliation. Low power interstitial laser hyperthermia offers a means of producing localised thermal necrosis of predictable nature and extent in these tumours without the need for resection, and which heals safely.

Light from a low power Nd-YAG laser is transmitted via a 200 m fibre which is inserted in the centre of the target organ. With a single fibre, lesions up to 1.6 cm (in rat liver) and 1.6 cm (in canine pancreas) in diameter can be produced with 1 W for 670–1,000 sec which heal safely, although higher powers (2 W) can cause lethal pancreatitis. Using multiple (4) fibres activated simultaneously (1.5 W for 670 sec per fibre) in liver, larger lesions of overlapping areas of thermal necrosis can be made, which measure 3.5 × 3 × 2.7 cm and heal by fibrosis and regeneration, but take 5–6 months to do so. In the pancreas with multiple fibres (1 W per fibre for 1,000 sec) inflammatory areas 6 × 2 cm can be produced that heal by inflammation and fibrosis by 3 months. Hyperamylasaemia is seen, but little clinical pancreatitis. Ultrasound has been used to insert and place fibres and to monitor accurately the evolution of the thermal damage. Although not yet proven, it is likely that necrotic tumour will heal in a similar manner to necrotic normal tissue in these organs, so making this technique potentially valuable for *in situ* necrosis of these difficult tumours, particularly as it may be possible to match the extent of laser necrosis to the extent of tumour spread with ultrasound control.

### Breast Cancer

### 4-hydroxyandrostenedione (4-OHA) as second line hormonal therapy in advanced breast cancer

P.A. Murray, L. Perry, J. Gilmore & P.N. Plowman

*St. Bartholomew's Hospital, London, UK.*

The major source of oestrogen production in postmenopausal women is by the aromatisation of circulating androgens in extra-adrenal sites. The aromatase inhibitor aminoglutethimide has a 25% response rate as second line hormonal therapy in advanced breast cancer but its usefulness is limited by toxicity, particularly in the elderly (*Hum. Toxicol.*, **6**, 227 (1987)).

4-OHA is a more potent specific aromatase inhibitor which has been shown to be active in breast cancer although the optimum dose is unknown (*Cancer Res.*, **47**, 1957 (1978)). Thirteen postmenopausal patients failing other hormonal therapy for advanced breast cancer have been treated with 4-OHA 250 mg i.m. fortnightly.

The mean serum oestradiol ($\pm$s.e.) (pmol l$^{-1}$) measured by radioimmunoassay prior to treatment and at weeks 1, 2 and 8 was $30.0 \pm 5.7$, $12.8 + 2.4$, $12.9 \pm 2.2$ and $15.9 \pm 2.6$, respectively.

Clinical response has been observed in 3 patients (PR) with disease stabilisation in 1. Progression has occurred in 6 patients and 3 are not yet assessable. Therapy has been well tolerated and apart from transient pain at the injection site there have been no significant side effects. 4-OHA 250 mg fortnightly produces a significant reduction in serum oestradiol levels in postmenopausal patients and the clinical response to date is encouraging. Its lack of systemic toxicity is an advantage over other second line endocrine therapy.

**Long term cosmesis and morbidity following lumpectomy and radical radiotherapy for breast cancer**

A.M. Brunt, A. Drury, H. Ellis, A.G. Goodman & R.H. Phillips

*Westminster Hospital, London, UK.*

Eighty-one consecutive patients referred by one surgeon (HE) following lumpectomy for breast cancer, and whom received post-operative radiotherapy under the care of one radiotherapist (RHP), were considered for cosmetic result and morbidity assessment. All patients received 4,840 cGy in 22 fractions to the breast and axilla, 5,940 cGy in 27 fractions to the supraclavicular nodes and a 2,000 cGy boost to the primary site. Cosmetic results were quantified on a four-point scale for telangiectasia matchline effect, fibrosis and the doctor and patient's overall assessment. The surgical scar was also examined and the morbidity was assessed by shoulder abduction, upper and lower arm mid-point circumference, and by pain in the breast, axilla and arm.

Forty-eight patients were assessed at a mean of 67.4 months from completion of radiotherapy (range 54–98 months), whilst 33 were non-assessable due to death, local recurrence or were lost to follow-up. Telangiectasia and fibrosis were slight or absent in 45 and 36 respectively and moderate in the remainder. A matchline effect was not observed in any patient. The surgical scar was not apparent in 11, apparent in 35 and there was major tissue loss in 2. In no case was there considered to be a serious distortion of the breast. There was no significant difference in the range of shoulder abduction or arm circumference between the treated and untreated sides. One patient reported severe pain in the arm, none did for the breast and axilla. We conclude that the long-term cosmetic appearance and morbidity suffered following lumpectomy and radical radiotherapy is highly satisfactory.

**Does flow cytometry accurately determine ploidy in ductal carcinoma *in situ* of the female breast?**

R. Carpenter, T. Cooke, J. Matthews, C. Dowle, K. Burn, A. Locker & R. Blamey

*Charing Cross and Westminster Medical School, Royal Liverpool Hospital and Nottingham City Hospital, UK.*

Flow cytometry is a convenient way of assessing tumour ploidy, but recent studies using this technique have cast doubt on earlier reports that ploidy determined by static cytometry is related to prognosis in breast cancer. To assess possible insensitivity of the automated method, we checked results of flow cytometry by simultaneous static cytometry in patients with pre-invasive breast cancer. Cell suspensions obtained by enzymatic disaggregation of 65 *in situ* carcinomas of the breast from the Nottingham breast clinic were stained by the Schiff reagent or diaminodio-2-phenylindol-dichloride (DAPI) and DNA content measured by microdensitometry (static cytometry) and flow cytometry respectively.

| | | *Static cytometry* | | |
|---|---|---|---|---|
| | | *Aneuploid* | *Diploid* | *Total* |
| Flow cytometry | Aneuploid | 31 | — | 31 |
| | Diploid | 16 | 18 | 34 |
| | Total | 47 | 18 | 65 |

Thirty-one lesions reported aneuploid on flow cytometry were confirmed by static cytometry, however, 16 of 34 identified as diploid were aneuploid on microdensitometry. Flow cytometry over diagnosed diploidy in 47% of lesions.

Flow cytometry is less sensitive than static cytometry in ploidy determination, which could explain discrepancies between the published studies. To correct this problem when diploidy is identified by flow cytometry, microdensitometry should subsequently be performed.

**An approach to the early identification of response to tamoxifen in advanced breast cancer**

R. Carpenter, A. Samuels, J. Matthews, S. Shousha, J.I. Burn, D. Wright & J. Powell

*Department of Surgery, Charing Cross and Westminster Medical School, London, UK.*

Breast cancer response to tamoxifen cannot be predicted reliably from oestrogen receptor status. Activity of tumour α-glycerol phosphate-dehydrogenase has been reported to increase following tamoxifen. We investigated the effect of tamoxifen on activity of this and other enzymes involved in lipid biosynthesis: α-glyceraldehydephosphatase-dehydrogenase and malic enzyme. Changes in cellular DNA content and thymidine kinase, an enzyme of DNA synthesis under oestrogen control, were also monitored.

Needle biopsies of primary breast cancer were assessed for histology, ploidy and enzyme activity before and 6 weeks after commencing tamoxifen. Tumour response was assessed by UICC criteria.

There was a significant reduction in thymidine kinase activity from $5.87 \pm 0.95$ to $1.98 \pm 0.41\,U\,mg^{-1}$ protein ($P<0.001$, paired $t$) in 10 patients whose disease did not progress over a mean of 4.6 months. In contrast, activity increased from $3.43 \pm 1.43$ to $3.95 \pm 1.52\,U\,mg^{-1}$ protein in 5 patients whose disease progressed ($\chi^2$, $P<0.02$). Neither treatment or tumour progression were related to changes in the other enzymes studied nor was response related to aneuploid ($n=14$) or diploid ($n=1$) DNA content.

Tamoxifen response was not related to activity of the enzymes of lipid metabolism tested in this study, however, oestrogen sensitive aspects of DNA synthesis, of which thymidine kinase is an example, appear more rewarding.

**Prospective evaluation of a low maintenance care policy for vascular access ('Hickman') catheters (VACs)**

S.R. Ebbs, J.A. Saunders & M. Baum

*Department of Surgery, King's College Hospital School of Medicine, London, UK.*

VACs are particularly suited to the administration of cytotoxics. As new low toxicity regimens allow home infusion of chemotherapy, we have prospectively evaluated low intervention catheter care ideally suited to this environment with great financial and patient comfort benefits.

Fifty-two women with advanced breast cancer were randomised to receive weekly epirubicin as either a standardised bolus injection through a peripheral vein or as a 24 hr infusion using a lightweight disposable pump (travenol infusor) through a single lumen VAC. Twenty-six women had VACs *in situ* for a total of 2,294 patient days (range 6 to 217 days. median 55 days). Two hundred and seventy-seven infusors were administered over this period (range 0 to 25, median 6).

No antibiotics, antiseptics, anticoagulants or occlusive dressing were employed. The VAC was used for blood sampling and the infusor connected. The patients returned to their own home where the following day they disconnected the infusor and 'recapped' the line, without flushing it.

*Complications:*

| | | |
|---|---|---|
| Occluded, haemorrhage or haematoma, damaged or leaked | | 0 enents |
| *Infections* | – exit site | 3 events |
| | – subcutaneous tunnel | 1 event |
| | – systemic | 0 events |
| *Catheter access* | – total occlusion | 0 events |
| | – infuse but not sample blood | 19 events |
| | – infuse and sample blood | 267 events |

Three exit site infections occurred in 2 women (11.5%); 1 patient developed inflammation around the subcutaneous tunnel (4%); no patients suffered systemic infection. No catheters became temporarily or permanently occluded during 286 accesses.

There is no need for heparinisation and bacteriocidal wound dressing for VACs.

### Correlation between medroxyprogesterone acetate blood levels and response in patients with breast cancer

A.D. Stockdale & A.Y. Rostom

*Department of Radiotherapy and Oncology, St. Luke's Hospital, Guildford, UK.*

Thirty females with advanced or locally recurrent carcinoma of the breast were randomly assigned to receive 1 g daily by mouth, of medroxyprogesterone acetate (MPA) as either provera, 100 mg x 10, 200 mg x 5, (Upjohn) or farlutal 500 mg x 2, (Farmitalia) tablets. MPA blood levels were measured during day 1 and on days 7, 14, 21 and 28. Serum cortisol levels are also available for the majority of patients. One patient had a complete and 4 patients partial responses to therapy (17%). Ten patients achieved stasis of previously advancing disease (33%). Despite significant differences in MPA concentrations between the 3 groups, objective responses and stasis were equally spread between preparations, and showed no correlation with either MPA concentration or suppression of serum cortisol. We find no evidence to suggest that high doses of MPA produce greater responses than achieved with low doses.

### Pharmaceutical aspects of domiciliary continuous infusion chemotherapy

G.J. Sewell, M. Allsopp, M. Northcott & C.G. Rowland

*Postgraduate Medical School, University of Exeter, Exeter, UK.*

In the Home Oncology Programme, Exeter (HOPE), antitumour agents are delivered as prolonged continuous infusions over 5–14 days by ambulatory infusion pumps. Pre-filled medication reservoirs are supplied to patients during out-patient visits. The drug must remain stable during refrigerated storage prior to use (up to 14 days) and also during infusion where the infusion temperature in a holster-worn ambulatory pump is 35–37°C.

We have determined the physical and chemical stability of cytarabine, carboplatin, epirubicin, 5-fluorouracil and mitozantrone infusions during storage in pump reservoirs and during delivery from ambulatory pumps. Stability-indicating HPLC methods were developed for this study. All five infusions were physically and chemically stable under storage conditions. There was no drug loss during infusion under in-use conditions with the exception of carboplatin where 3% of the drug degraded to cisplatinum. In further studies with α-interferon-2b infusion (3 mU), changes in the relative ratio of interferon monomers occurred with the appearance of degradation products under storage and in-use conditions. This was attributed to monomer interconversion and oligomer formation.

With the exception of α-interferon, all of the infusions were sufficiently stable for inclusion in the Home Infusion Programme and have been in clinical use for at least 6 months.

### Pharmacokinetic studies on mitozantrone in prolonged continuous infusion regimes

M. Northcott, G. Sewell, M. Allsopp & C.G. Rowland

*Postgraduate Medical School, University of Exeter, Exeter, UK.*

In a phase II study with 22 patients, advanced metastatic breast cancer was treated with prolonged continuous infusion of mitozantrone (2 mg day$^{-1}$ x 14 days). Although the dosage regime of the continuous infusion was empirically derived, response rates were encouraging (CR = 26%, PR = 26% at 6 months).

We have carried out pharmacokinetic studies with some of these patients and have compared pharmacokinetic parameters obtained in continuous infusion regimes with those reported for traditional bolus or rapid infusion regimes. Pharmacokinetic parameters will also be related to clinical response/toxicity with the long-term objective of optimising drug dosage in continuous infusion schedules. A solid-phase extraction procedure and a sensitive HPLC assay were developed and validated to determine mitozantrone in plasma. The limit of detection was 500 pg ml$^{-1}$.

The mean steady-state mitozantrone plasma level was 1.81 ng ml$^{-1}$ (range 1.13–4.39, = 6) and the mean area under the curve (AUC) was 0.517 μg.h ml$^{-1}$ (range 0.35–0.74 μg.h ml$^{-1}$). This compares with peak plasma levels of 683 ng ml$^{-1}$ (Van Belle *et al.*, *Cancer Chemother. Pharmacol.*, **18**, 27 (1986)) and AUC values of 1.45 μg.h ml$^{-1}$ (Ehninger *et al.*, *Investigational New Drugs*, **3**, 109 (1985)) reported for bolus regimes (14 mg m$^{-2}$ over 30 min). Our results suggest that peak-plasma levels are not essential for clinical response and that availability of drug to the tissues (determined by AUC values) are of a similar order in both regimes.

### Enhancement by medroxyprogesterone acetate (MPA) of cytotoxic effects of adriamycin (ADR) on breast cancer cells

N.A. Shaikh, A.M. Owen, M.W. Ghilchik & H. Brausberg

*The Breast Clinic and Department of Chemical Pathology, St. Mary's Hospital, London, UK.*

Cyclical sequential hormonochemotherapy involving oestrogen, MPA and alternate use of two combinations of cytotoxic drugs has produced very promising responses in patients with advanced breast cancer (Ghilchik *et al.*, *Br. Med. J.*, **295**, 1172 (1987)). In order to assess the positive contributions of hormonal agents in this therapeutic schedule we have examined the effects of MPA on the efficacy of individual drugs using human breast cancer cells (MCF-70)

in culture. Monolayers of cells were exposed to MPA or control medium for 48 h followed by drug for 24 h. The washed cells were then allowed to proliferate with growth medium for 3 days before being harvested and counted. MPA significantly ($P<0.025$ or lower) enhanced the effect of ADR (20 nM) in 4 of 5 experiments. The effect was observed at 10 nM MPA and maximal at 40 nM. Exposure to oestradiol (1 nM) for 24 h prior to MPA treatment further increased the action of MPA on the efficacy of ADR, while oestradiol followed by control medium (48 h) did not alter the drug effect. A subline of MCF-7 cells unresponsive to the growth inhibitory action of MPA responded to the hormone with respect to enhancement of drug action. Similar effects of MPA have been observed with respect to the actions of methotrexate and vincristine (Shaikh *et al.*, *Steroid Biochem*, **28** (Suppl.) 158S (1987)) and other drugs are under investigation. The results support our clinical findings and suggest that sequential hormonotherapy using MPA offers an advance over other treatments and may benefit patients even if their tumours have escaped or failed to respond to growth inhibition by the hormone.

### Development of a new transplantable hormone-responsive rat mammary tumour model

S.A. Eccles, H.P. Purvies & M. Jarman

*Institute of Cancer Research, Belmont, Surrey, UK.*

The pre-clinical evaluation of new anti-oestrogen drugs requires that their efficacy should be demonstrable in animal tumour systems. Currently employed models include primary chemically-induced (DMBA and MNU) rat tumours, and human mammary tumour cell lines grown as xenografts in nude mice. We set out to develop a transplantable hormone-responsive tumour with reproducible growth and oestrogen sensitivity in syngeneic rats.

From a tumour (induced by the s.c. implantation of an oestrogen pellet) which initially was only transplantable in oestrogen-supplemented female rats, we developed two cell lines - OES HR 1 and 2 - which would grow in the absence of additional oestrogen. Both tumours are hormone responsive in that their growth is enhanced by oestrogen supplementation and totally inhibited in males or ovariectomised females. In addition, both tumour cell lines regress or show stasis of growth in response to treatment *in vivo* with the following 'anti-oestrogen' drugs: 4 hydroxy-androstene dione, aminoglutethimide and tamoxifen. Under certain dosage schedules tamoxifen was shown to be oestrogenic, allowing an examination of this phenomenon in more detail. These tumour lines will serve as a useful adjunct in the screening of anti-endocrine agents. Also, since in the presence of oestrogen they are capable of lymphatic and haematogenous dissemination, we intend to investigate autocrine and paracrine influences in progression towards growth factor independence and metastasis.

### Iodostearic acid inhibits mammary carcinogenesis induced by nitrosomethylurea in rats

S.B. Kelly[1], N.A. Habib[1], C.B. Wood[2], K. Apostolov[2], B. Baker[2], M.J. Hershman[2] & R.C.N. Williamson[2]

[1]*Department of Surgery, Bristol Royal Infirmary, Bristol and* [2]*Department of Surgery, Royal Postgraduate Medical School, London, UK.*

Malignant transformation of the cell is accompanied by a decrease in membrane rigidity, resulting in part from the desaturation of stearic acid to oleic acid. The aim of this study was to investigate the influence of stearic acid on tumour development *in vivo*.

Nitrosomethylurea (NMU) rapidly induces mammary carcinomas in rats. Administration was by i.v. injection via the tail vein at a dose of $5\,\mathrm{mg}\,100\,\mathrm{g}^{-1}$ body weight. Two groups of female Sprague–Dawley rats received either NMU ($n=20$) or NMU plus iodostearic acid ($n=23$). Gas liquid chromatography was used to study changes in the fatty acid composition of rat erythrocyte membranes during mammary carcinogenesis.

By week 16, 19 of 20 rats in the NMU group and 8 of 19 rats in the NMU plus iodostearic acid group developed tumours. The mean number of tumours/rat was 3.8 in the NMU group and 2.7 in the NMU plus iodostearic acid group.

The mean saturation index (ratio of stearic acid to oleic acid) of rat erythrocytes was $2.0\pm0.3$ in rats receiving normal saline and $1.09\pm0.28$ in the NMU group ($P<0.001$).

These data indicate that iodostearic acid inhibits tumour development in rats. In addition, the onset of tumours is associated with a fall in the saturation index of rat erythrocytes.

### Gastro-intestinal Tract Malignancy

### Comparison of prognosis of preoperative and operative perforation of the colon in colorectal cancer

M.P. Tilston & N.S. Ambrose & M.R.B. Keighley

*General Hospital, Birmingham, UK.*

During the 3 year period in which we have been running a specific clinic for follow-up of colorectal cancers, 12 patients have presented to our unit with intestinal obstruction and signs of perforation (group 1). In this series we have compared the clinical course of these patients with that of patients who sustained inadvertent colonic perforation at operation (group 2). Mean age and sex distribution was similar in the 2 groups, as was Dukes' staging and degree of differentiation. However, a single patient with carcinoid of the appendix presented with peritonitis. Left sided tumours predominated in both groups but rectal tumours, which accounted for the vast majority of those undergoing operative injury (65%), were notably absent from those presenting with peritonitis, presumably due to the capacious nature of that portion of the bowel. Mean follow-up of group 1 was 27 months (1–124 months) and of group 2 was 56.5 months (11–135 months). At the time of writing 11 from group 1 are alive (92%), 7 of whom (58%) have no evidence of recurrence. In group 2, 14 (70%) are alive but only 8 are free of disease. Only 1 patient in group 1 has died of metastatic disease, whereas 6 patients (30%) in the latter group succumbed to carcinomatosis. The mean time to appearance or detection of recurrent disease was 17.4 months in group 1 and 27.1 months in group 2.

### Does postoperative sepsis influence the prognosis of colorectal cancer?

M.P. Tilston, C. Hall, N.S. Ambrose & M.R.B. Keighley

*General Hospital, Birmingham, UK.*

We have followed-up all colorectal cancers in a specific clinic over the last three years, and during this period 24 patients had postoperative intra-abdominal or intrapelvic sepsis (19 with clinically evident sepsis and 5 with radiological evidence of anastomotic leakage). The subsequent clinical course of these patients is compared with that of an age/sex and stage matched cohort ($N=25$), who did not have evidence of postoperative sepsis. Mean age of group 1 was 65.2 years and of group 2, 66.7 years. Left sided tumours predominate in both groups, with a slightly higher proportion of right sided tumours in group 2 than in group 1. Mean follow-up of both groups exceeded 2 years (group 1: 149.5 months, group 2: 38.4 months). Twenty-one patients in group 1 and 23 in group 2 are alive and available for follow-up, 2 cases from each group having died from carcinomatosis and a further 1 from group 1 having died from cardiovascular disease. However, overall recurrence rates differ markedly. In group 1, 5 patients have had evidence of local or distant disease (21%), whereas 10 patients from group 2 (40%) are affected. Although this does not achieve statistical significance, there would appear to be an important trend towards improved recurrence rate with postoperative sepsis. This might conceivably arise from either a general non-specific stimulus to the immune system, brought about by infection or by the occurrence of an unfavourable environment for implantation to take place.

### Cancer in Crohn's disease after diversionary surgery: A study of the crypt cell production rate

M.C. Winslet, D.J. Youngs, A. Allan & M.R.B. Keighley

*General Hospital, Birmingham, UK.*

A high incidence of tumours has been reported (Greenstein *et al., Am. J. Surg.*, **135,** 86 (1978)) after diversionary surgery for Crohn's disease which is related to the duration of Crohn's disease rather than that of the bypass. To assess whether this high incidence is due to changes in colonic cytokinetics after bypass, the rectal crypt cell production rate (CCPR) has been assessed in patients with defunctioned Crohn's colitis (DCC) ($n=9$) and a control group (DC) for a non-inflammatory condition ($n=6$). The mean duration of Crohn's disease was 58 (12–134) months. The mean diversionary period was 9 (2–16) months for Crohn's disease and 3 (1–8) months for controls. Results were compared with controls (C: $n=21$) and patients with ileal Crohn's disease (CD: $n=9$) and Crohn's proctitis (CP: $n=15$) who had not been defunctioned.

| | *DCC* | *DC* | *C* | *CD* | *CP* |
|---|---|---|---|---|---|
| CCPR ± s.e.m. (cells crypt$^{-1}$ h$^{-1}$) | 2.5±0.4 | 2.9±0.6 | 3.2±0.5 | 4.0±0.7 | 2.8±0.7 |

This study has failed to demonstrate any change in the cellular proliferation rate in the excluded colon after a disease duration of 5 years and a diversionary period of 9 months. It suggests that the risk of carcinogenesis in bypassed bowel is not due to changes in cell kinetics.

### Combined external beam and intracavitary radiotherapy for carcinoma of the oesophagus

P.J.D.K. Dawes, M.B. Clague & E.M. Dean

*Regional Radiotherapy Centre, Newcastle General Hospital, Newcastle upon Tyne, UK.*

Thirty patients have been treated with megavoltage external beam radiotherapy and intraluminal radiotherapy using the Selectron oesophageal applicator and external beam or 8 MV photon therapy.

Twenty-six patients have received 30 Gy in 10 daily fractions through either 2 or 3 field plans. Either on the last day of treatment or shortly afterwards a further 10 Gy has been given at 1 cm from the sources using the Selectron oesophageal applicator. Twelve Cs 137 sources of initially 40 mCi strength are used and the treatment is delivered in approximately one and a half hours. Four patients have received external beam doses of 50 Gy in 4 weeks (daily fractionation). This is the department's standard radical treatment and after this a further 10 Gy is given at 1 cm using the Selectron.

At 6 months the survival is 65%. A group of 16 patients treated radically in the 3 years prior to introduction of the Selectron treatments for carcinoma of the oesophagus had a 6 month survival of 50%

In view of the fact that the Selectron group included 3 patients with recurrent disease following either previous surgery or radiotherapy and only 2 patients have not been treated with the Selectron, due to poor general condition, these preliminary results are very interesting.

### The influence of dietary fat on the fatty acid profile of red blood cells (RBC) and adipose tissue in patients with colorectal cancer (CRC)

J.P. Neoptolemos, H. Clayton, M. Nicholson, J. Ollerenshaw, B. Johnson, J. Mason, K. Manson, R. James & P. Bell

*Departments of Surgery, Medicine and Nutrition, University of Leicester, Leicester, UK.*

A reduced ratio of RBC stearic to oleic acid ratio has been proposed as a highly sensitive marker of CRC. However, objections have been raised about matching of patients and the role of dietary fat has not been previously assessed.

Using gas liquid chromatography, the fatty acid profile in RBCs and adipose tissue was determined in 49 patients with CRC and 49 age and sex matched controls. In the CRC group there were marginally increased levels (mean s.e.m.) of stearic acid (18.1±0.4 *vs.* 17.2±0.3, $P=0.06$) and oleic acid (20.3±0.4 *vs.* 19.0±0.4; $P=0.06$) and decreased arachidonic acid (22.0±0.4 *vs.* 23.4±0.6, $P=0.04$). There was no difference in the RBC stearic to oleic acid ratio between the two groups (0.9±0.02 *vs.* 0.91±0.01); there was no correlation of this ratio with the Dukes' staging. The polyunsaturated/saturated (P/S) ratio of dietary fats and adipose tissue was correlated in both groups ($P<0.001$). The RBC P/S ratio was also correlated with the dietary P/S ratio in the cancer group ($P<0.01$).

In conclusion, small differences of RBC fatty acids in CRC are linked to dietary fat. The RBC stearic to oleic acid was not diagnostic of CRC in this study.

### Resection of the rectum for cancer in a district general hospital

R.A. Cobb[1], H. Reece-Smith[1] & D.C. Britton[2]

[1]*Battle Hospital, Reading and* [2]*Royal United Hospital, Bath, UK.*

A retrospective study of all patients who had either an anterior resection (AR), or an abdomino-perineal resection (AP) for rectal cancer in Bath Health District in the 12 years 1967–1978 was undertaken with a minimum follow-up of 5 years.

There were 329 operations performed (AR: 91, AP: 238). Serious complications were recorded in 22.2% of patients (AR: 28.6%, AP: 19.7%). The operative mortality was 14.0% (AR: 17.6%, AP: 12.6%). Both morbidity and mortality were related to the age of the patient. Following anterior resection the operative morbidity and mortality was related to the distal clearance of the tumour. There was no correlation between the grade of the surgeon and morbidity or mortality.

Crude 5 year survival was 27.6% overall, ranging from 68.1% for Dukes' A tumours to 5.5% in patients with distant metastases (AR: 81.2% to 0%, AP: 61.3% to 9.1%). Survival was not related to the distal clearance (AR) or distance of the tumour from the anus (AP). Local recurrence occurred in 27.4% (AR: 30.8%, AP: 26.1%). The mean interval from operation to development of local recurrence was 18 months (AR: 14, AP: 19). The mean interval from diagnosis of local recurrence to death was 11 months (AR: 12, AP: 10).

Most reports have been from teaching centres or of a single surgeon's experience, and have shown lower operative mortality and better survival rates. Our results therefore give no cause for complacency.

### Radiotherapy for epidermoid anal carcinoma

D. Otim-Oyet, A. Horwich, J. Crow & H.T. Ford

*Royal Marsden Hospital, Surrey, UK.*

A retrospective analysis on 75 patients treated at the Royal Marsden Hospital between 1958 and 1986 showed that 29 presented with Lyons T2 tumours and 27 Lyons T3. Twenty patients (26.6%) had inguinal nodal metastases and 3 systemic dissemination. Definitive treatment was radiotherapy in 55 patients, 46 of whom received a radical dose of 60 Gy/30 #/6 weeks, or NSD equivalent. Four of these had combined modality treatment with 5FU and mitomycin-C. Of 20 patients treated by surgery 9 received pre- or post-operative radiotherapy. The median follow-up of survivors was 49.8 months. The actuarial case specific survival at three years was 55% and this was adversely influenced by increasing stage and histological grade. In the radiotherapy group 72.2% (13/18) of T1, T2 no patients were controlled, and 3 of 5 relapsing patients were salvaged by surgery. Five of eight patients who developed distant metastases also had uncontrolled primary tumours. Six of 20 (30%) patients treated by surgery recurred loco-regionally and none were salvaged by radiotherapy.

It is concluded that radiotherapy alone is effective treatment for small epidermoid anal carcinomas, and has the advantage of preserving anal continence. More advanced tumours probably require combined modality treatment.

### Genito-urinary Cancer

### Toxicity of a carboplatin, etoposide, bleomycin combination as primary chemotherapy of metastatic malignant teratoma

R.A. Dealey & T. Sheehan

*Velindre Hospital, Cardiff, UK.*

In May, 1986 we commenced using the combination of carboplatin 350 mg m$^{-2}$ day 1, etoposide 120 mg m$^{-2}$ days 1, 2 and 3 i.v. with cycles repeated after 21 days, with bleomycin 30 mg i.m. weekly to a total dose of 360 mg. Subsequently the dose of carboplatin has been increased to 750 mg in patients with glomerular filtration rate (GFR) greater than 90 ml min$^{-1}$, and later to 900 mg where GFR exceeded 120 ml min$^{-1}$ and initial platelet count exceeded $200 \times 10^6$ l$^{-1}$.

No major toxicity has been encountered. Nausea and vomiting have occurred but have not affected delivery of chemotherapy. Temporary alopecia was invariable. In some patients skin reactions resulted in cessation of bleomycin. No patients showed renal toxicity, or suffered symptomatic neurotoxicity or pulmonary toxicity. Seventy-eight cycles in 22 patients have been studied for haematological toxicity. No cases of WHO grade IV toxicity, septicaemia or haemorrhage have occurred. In only 3 cycles did the nadir platelet count fall below $50 \times 10^6$ l$^{-1}$.

We conclude that the toxicity of this combination is minimal and that there can be further escalation of dosage in patients with high GFR.

### Prediction of late renal damage in stage C metastatic teratoma

G. Duchesne, C. Barton, M. Williams & A. Horwich

*Institute of Cancer Research and Royal Marsden Hospital, Sutton, UK.*

Patients presenting with large (>5 cm) abdominal metastases from malignant non-seminomatous germ-cell tumours may be at particular risk of long-term renal damage from the combined effects of ureteric obstruction and cisplatin chemotherapy. Renal function ($^{51}$EDTA clearance) and the presence of hydronephrosis (HN) and/or renal cortical atrophy were studied prior to and following chemotherapy in 114 patients to identify potential risk factors for late renal damage. Low EDTA clearance at presentation was significantly more common in the presence of HN (21/40 *vs.* 8/74 without HN, $P<0.01$). In the absence of HN patients treated with cisplatin retained acceptable renal function at the completion of chemotherapy (clearance 102 ml min$^{-1}$). Of 32 patients with HN receiving cisplatin, 6 had renal atrophy at presentation, and 6 developed late atrophy. The development of atrophy and poor renal function at the end of chemotherapy were both associated with failure to improve EDTA clearance by at least 10% after one month of treatment, and with presenting clearances below normal. By contrast 7 patients with HN receiving carboplatin showed an overall improvement in renal function (95→124 ml min$^{-1}$) and none developed renal atrophy. Thus hydronephrosis and low EDTA clearance at presentation, together with failure to show an early improvement in renal function (through relief of obstruction) predict for late renal damage, which may be avoided by the use of carboplatin or possibly by the use of ureteric stenting in bad risk patients.

### Cisplatin-induced renal tubular defects

L.M. Matheson[1], J.F. Smyth[2] & C.P. Swainson[2]

[1]*University Department of Clinical Oncology, Western General Hospital and* [2]*Royal Infirmary, Edinburgh, UK.*

Cisplatin administration is known to cause renal tubular defects. A retrospective survey of 165 patients (aged 11–70 years) treated in Edinburgh (1978–1987) with various cisplatin regimens has now been completed; all patients received mannitol and intensive hydration but no routine magnesium supplementation. Fifteen of 165 (9%) developed asymptomatic systemic acidosis (plasma bicarbonate <21 mmol l$^{-1}$) and in 10 patients (6%) mild acidosis persisted. In 2 patients, recovery occurred after 9 months. Hypomagnesaemia developed in 103/148 (69%), pre-therapy [Mg] $0.79 \pm 0.08$ mmol l$^{-1}$, post-therapy $0.56 \pm 0.01$ mmol l$^{-1}$, $P<0.0001$. In all other patients, plasma [Mg] remained within the normal range but fell significantly from pre-treatment levels (pre $0.82 \pm 0.07$ mmol l$^{-1}$, post

$0.76 \pm 0.05\,mmol\,l^{-1}$, $P<0.0001$). Relationship between post-therapy plasma bicarbonate and magnesium levels as below:

| | *Plasma [Mg] mmol$^{-1}$]* | |
|---|---|---|
| *Plasma [bic] mmol $l^{-1}$* | *<0.71* | *≥0.71* |
| *<21* | 8 (5%) | 4 (3%) |
| *≥21* | 95 (64%) | 38 (26%) |

There was no correlation between bicarbonate and Mg levels with cumulative cisplatin dosage (range 100–900 mg $m^{-2}$). Plasma potassium levels remained unchanged.

The majority of patients develop hypomagnesaemia while a minority develop tubular acidosis. The incidence and severity of incomplete renal tubular acidosis is under investigation.

### A report of surveillance of stage 1 seminoma post orchidectomy

C.J. Tyrrell & F. Daniel

*Department of Radiotherapy and Oncology, Plymouth General Hospital, Plymouth, Devon, UK.*

Between April 1984 and December 1987, 35 patients with seminoma were referred.

All were investigated with serum markers (AFP and BHCG) lymphogram, CT scan of abdomen and ultrasound of testes.

Two were excluded because of elevated alpha foetoprotein.

Of the 28 stage 1 patients, 25 were considered to be of good prognosis and received no adjuvant radiotherapy.

Of these, 3 patients have relapsed (12%) after 5, 6 and 6 months respectively. Mean follow up is now 26 months (6–50 months).

All patients remain alive and disease-free.

The relapse rate with stage 1 seminoma treated by orchidectomy alone and careful observation may be lower than stage 1 teratoma and may be a safe alternative to adjuvant radiation.

### Dose of intravesical chemotherapy

J.R.W. Masters

*Institute of Urology, University College, London, UK.*

The response of tumours to systemic chemotherapy is proportional to the dose rate achieved. Dose rate usually is calculated as the area under the plasma drug concentration – time curve (AUC or C x T). In contrast, this concept is not used for intravesical chemotherapy. Instead, it is the weight of drug instilled into the bladder that is considered to be the dose. Thus, a 60 mg instillate of ThioTEPA gives a twofold higher dose than a 30 mg instillate, regardless of the volume of fluid or the period of exposure. The drugs usually are dissolved in a corresponding volume of solvent, the 60 mg in 60 ml and the 30 mg in 30 ml, giving a twofold difference in 'dose', but at identical concentrations of $1\,mg\,ml^{-1}$. It is probable that the response of superficial bladder cancer to intravesical chemotherapy also is proportional to the drug concentration and period of exposure (C x T). Many published studies of intravesical chemotherapy record neither the drug concentration nor the period of exposure, only the weight of drug, and consequently optimum doses have not been calculated. If C x T were used to calculate the dose, intravesical chemotherapy could be made safer, cheaper and more efficient, as less drug would be needed to achieve the same dose. By reducing the volume of instillate to the minimum quantity required to cover the tumour and urothelium, the patient may be able to tolerate the therapy for a longer period, thus further increasing the dose rate. These measures could increase the success rate of intravesical chemotherapy.

### Megestrol acetate as second line hormone therapy in advanced carcinoma of the prostate

F. Daniel, C.J. Tyrrell & P.M. MacLeod

*Department of Radiotherapy and Oncology, Plymouth General Hospital, Freedom Fields, Plymouth, UK.*

Twenty-two patients with advanced metastatic carcinoma of the prostate previously managed with first line hormone therapy (orchidectomy, LHRH analogue or diethylstilboestrol) have received megestrol acetate 160 mg daily, at disease progression. Patients have been followed up for a period of up to 36 months with a minimum follow-up of 6 months.

Twenty-one patients are evaluable for toxicity and response. The treatment was well tolerated with minimal morbidity. Two patients reported excess weight gain and 2 developed exacerbations of congestive cardiac failure which stabilized with reduction in dosage of megestrol to 80 mg daily. Eight patients (38%) reported subjective improvement in their symptoms. An objective response as assessed by serial rectal examination, bone scanning, X-rays and prostatic acid phosphatase assay according to the criteria of the NPCP was seen in 6 patients (28%). There were no complete or partial responses, all responses being stabilization of disease of 6–12 months duration (mean $40.3 \pm 12.8$ weeks).

We have reviewed the controversial role of second line hormonal measures in advanced carcinoma of the prostate and conclude that megestrol acetate has a useful role and is as effective as other measures with the advantage of minimal morbidity.

### Localised prostatic carcinoma: A retrospective analysis comparing radical radiotherapy with hormonal manipulation

T. Joannides, D. Tong, M.R. Jani & H. Kinder

*Department of Radiotherapy and Oncology, Guy's Hospital, Department of Urology, Guy's and St. Peter's Hospitals, London, UK.*

To assess the efficacy of local treatment of carcinoma of the prostate at our institution we carried out a retrospective analysis of patients treated for localised carcinoma of the prostate (T0–4, NX, M0) between January 1976 and December 1985. Following initial treatment of obstructive symptoms with either transurethral resection of the prostate or retropubic prostatectomy the patients were then allocated to either radical radiotherapy, orchidectomy, or diethylstilboestrol.

This is an on going analysis which will include over 300 patients but we present an interim report of 43 patients (mean age: 66 years, age range: 47–79 years) treated by radical radiotherapy (6,000 cGy in 30 daily fractions over 6 weeks), and 30 patients (mean age: 72 years, age range: 53–84 years) treated by bilateral subcapsular orchidectomy or diethylstilboestrol.

Local tumour control was significantly better in the radiotherapy group (76%) compared to the hormone group (45%) although there was no significant difference in the time to first systemic disease progression overall. Probability of local control decreased with poorly differentiated tumours and T3–T4 tumours fared worst in both groups.

Radiation therapy was tolerated well with a 9% late complication rate including proctitis, tenesmus, and bladder neck contracture.

### The diagnosis of carcinoma of the prostate, bladder and kidney by the CA-50 radioimmunoassay test

S.B. Kelly[1], N.A. Habib[1], M.J. Hershman[2], R.C.N. Williamson[2] & C.B. Wood[2]

*Departments of Surgery, [1]Bristol Royal Infirmary and [2]Royal Postgraduate Medical School, London, UK.*

Carcinoma associated antigen (CA-50) is a tumour associated carbohydrate antigen, defined by the monoclonal antibody C50, which has been raised against a colorectal adenocarcinoma cell line. The aim of this study was to assess whether CA-50 can differentiate patients with urological carcinomas from normal subjects and those with benign disease. A radioimmunoassay was used for the detection of CA-50 in the serum of 50 normal subjects, 86 patients with benign disease and 104 patients with urological carcinomas. Serum levels in all 50 normal subjects and 83 of 86 patients (97%) with benign disease were $<17\,U\,ml^{-1}$, while 49 of 104 patients (47%) with carcinoma had levels $>17\,U\,ml^{-1}$. The sensitivities for prostatic, bladder and renal carcinomas were 43%, 62% and 47% respectively. In patients with prostatic carcinoma, the sensitivities were 0% for well differentiated carcinomas, 33% for moderately differentiated carcinomas, 67% for poorly differentiated carcinomas and 71% for metastatic disease. The sensitivities for non-invasive and invasive bladder carcinomas were 42% and 89% respectively. Therefore this test may prove useful in the diagnosis of patients with urological malignancies.

### Lonidamine and radiotherapy in head and neck cancer

L. Magno[1], F. Terraneo[1], F. Bertoni[1], M. Tordiglione[2], D. Bardelli[2], M. De Gregorio[3] & G.B. Ciottoli[3]

*[1]O. Alberti Inst., Civil Hospital, Brescia, I; [2]Civil Hospital, Varese, I and [3]F. Angelini Research Inst., Rome I, Italy.*

Lonidamine (L) an inadazole carboxylic acid derivative was shown to have an antitumour activity in phase I–II studies in lung, breast and prostate cancer. L was also shown to potentiate the effects of X-rays both *in vitro* and *in vivo* by inhibiting the recovery of cancer cells from potentially lethal damage. Its tolerance was satisfactory when used either alone or in combination with radiation. Since January 1983, 95 patients with cancer of the oral cavity have been admitted to this double-blind randomized study. Patients, stratified by the presence (or absence) of clinically detectable lymph nodes (N), were allocated to the L+radiotheapy (LRT) or placebo+radiotherapy (PRT) group. Radiotherapy (1.5 Gy twice daily for 5 days a week by 60 Co or 7–12 MeV photon beam) was given up to 60–66 Gy on tumour and clinically affected N. Clinically uninvolved N were irradiated up to 45 Gy. L (150 mg 3 times daily) was administered for 3 months starting from 3 days before irradiation. Eight-six patients (42 in the LRT and 44 in the PRT group) were adequately treated and evaluable for response. Twenty-nine (69%) and 26 (59%) complete responses (CR) were observed in the LRT and PRT groups, respectively. The estimated median duration of CR was >1,200 days for LRT and 590 days for PRT ($P=0.058$). Life table % estimate of local control was significantly superior to the LRT group ($P=0.043$). The estimated disease-free patients at 2 years were 52% and 29% in the LRT and PRT groups, respectively. Neither an increase in the acute reaction to radiation nor haematological toxicity was observed.

### Head and Neck Cancer

### Radiotherapy of paranasal sinus malignancy

B.J. Haylock, G. John & I.C.M. Paterson

*Velindre Hospital, Cardiff, UK.*

Between 1974–83, 72 cases were referred to this centre. The mean age was 61 years (range 41–91). The commonest presenting symptoms were nasal obstruction, facial swelling and pain. Sixty-five percent had squamous cell carcinoma, of these 81% arose in the maxillary sinus and 19% in the ethmoids. Most had advanced disease. Eighty-nine percent of maxillary sinus cases were UICC stage 3 or 4 at presentation. The majority had radiotherapy alone or pre-operative irradiation followed by elective surgery. Patients given radical radiotherapy received 10 fractions, 3 fractions/week or, 20–30 daily fractions over 4–6 weeks, with total doses between 40 and 60 Gy (mean TDF 96).

Actuarial analysis of all squamous carcinoma revealed a 5 year survival of 37.4% and relapse free survival of 31.6%. For those receiving radical doses the 5 year survival was 46%. Survival was the same for patients receiving radical radiotherapy alone or pre-operative irradiation and surgery. Despite fractional doses in excess of 4 Gy in some cases, late radiation damage was confined to 5 cases of ocular damage (total blindness in one) and one of osteoradionecrosis of the maxilla.

The optimum treatment of these malignancies continues to be controversial.

### Role of radiotherapy in the suppression of parotid secretions

A.C.R. Robinson[1], G.G. Khoury[1] & P.M. Robinson[2]

*[1]University of Leeds, Department of Radiotherapy, Cookridge Hospital, Leeds; and [2]University of Leeds, Department of Surgery, Leeds, UK.*

Suppression of salivary flow is of value in the management of salivary fistulae and sialectasia. It may also be beneficial in mentally defective patients and those with neurological palsies lacking control of their salivation. Available treatments are anticholinergics, parotidectomy, neuroblastive procedures and radiotherapy.

Nine cases were treated by irradiation between January 1978 and December 1987, the underlying pathology being sialactasis (5), traumatic fistulae (2), post-operative parotitis (1), excessive dribbling in a mentally subnormal patient (1). Four had previous surgery. The median age was 50 years (range 21–83). A direct field was applied over the parotid gland and treatment was given with 250 kev or $Co^{60}$. The mentally subnormal patient had both parotids treated. A number of different regimes were used from 3 Gy in 6 fractions over 18 days to 30 Gy in 6 fractions over 8 days. Eight patients had complete resolution of symptoms, one had partial relief and no relapses were seen after a median follow-up of 26 months (range 1–107 months). Low doses were as effective as high doses in suppressing salivation. There were no acute or long term side effects.

Radiotherapy is an effective treatment for these benign conditions. It avoids long term medication and the need for surgery, important considerations in the elderly or mentally subnormal. It can also be of benefit in those who have failed surgery.

### Gynaecological Cancer

### Carcinoma of vagina: A 15 year experience at a regional radiotherapy centre

A.C.R. Robinson[1], G.G. Khoury[1] & N. Fazlani[2]

[1]*University Department of Radiotherapy and* [2]*Regional Radiotherapy Centre, Cookridge Hospital, Leeds, UK.*

Vaginal cancer is rare, prospective studies are difficult to mount and require the cooperation of many centres. We reviewed all case notes of patients treated at Cookridge Hospital over a 15 year period, to identify prognostic factors, optimal management policy and evaluate treatment morbidity.

Sixty-three patients, with a median age of 69 years were evaluated. Follow-up ranged from one to 120 months (median for survivors 40 months). Sixty had squamous cell carcinomas and three adenocarcinomas. The actuarial 5 year survival for cell patients was 32.4%.

In early stages (FIGO I+II) the local control and survival seen with different treatment policies will be presented. The influence of stage, histological grade, age and presenting haemoglobin levels on survival were analysed. Treatment complications were documented.

### The role of lymphography in the post-operative management of carcinoma of the endometrium

J.D. Graham, P.R. Blake and C. Parsons

*Departments of Radiotherapy and Diagnostic Radiology, Royal Marsden Hospital, London, UK.*

In order to assess the role of lymphography in the post-operative management of carcinoma of the endometrium 57 consecutive cases were analysed retrospectively. Four patients with recurrent disease were excluded. Of 53 patients, 48 had undergone TAH&BSO and 5 D&C only. Distribution by clinical stage was: 40 stage I (75%), 8 stage II (15%), 3 stage III (6%) and 2 stage IV (4%). Bipedal lymphography was performed in 41/53 cases (77%).

There was unilateral pelvic lymphadenopathy detected in 3 cases (2 stage I, 1 stage II). No case of para-aortic node involvement was seen. The use of post-operative radiotherapy either by intracavitary therapy alone or in combination with external beam is made on the basis of stage, histological grade and depth of myometrial invasion. In no case did lymphography alter the management decision made on the basis of these criteria, although the 3 cases with positive lymphograms received an additional 5 Gy to the involved side of the pelvis. In a short median follow-up of 9 months, a total of 4 cases have relapsed (2 stage I, 2 stage II). All had normal lymphography at presentation and received both external beam radiotherapy to the pelvis and a single intracavitary vault insertion. The pattern of relapse was: Pelvis alone 1, pelvis and lung 2, lung alone 1 and was related to tumour grade.

Bipedal lymphography has no role in the post-operative management of carcinoma of the endometrium.

### Intraperitoneal Yttrium-90-labelled antibodies in ovarian cancer

J.S.W. Stewart, V. Hird, H.E. Lambert & A.A. Epenetos

*Imperial Cancer Research Fund and Department of Clinical Oncology, Hammersmith Hospital, London, UK.*

Between March 1987 and March 1988, 20 patients with ovarian cancer received intraperitoneal Yttrium-90-labelled monoclonal antibodies to tumour associated antigens. Blood and urine Yttrium-90 activities were monitored for 5 days after treatment. The non-specific radiation dose to the peritoneal cavity was measured by lithium fluoride thermo-luminescent dosimetry. Yttrium-90-labelled antibodies were absorbed from the peritoneal cavity into the systemic circulation with 20% of the injected dose at 40 h. Bone marrow suppression is the dose limiting factor with moderate toxicity after 15 cCi Y-90-labelled monoclonal antibody. The estimated radiation dose to the marrow from the circulating Yttrium-90-labelled antibody was $<50$ cGy. Bone marrow irradiation from unchelated Yttrium-90 absorbed onto bone, may account for the discrepancy between the estimated radiation dose to marrow and observed toxicity. Although this was primarily a phase I study we observed a partial response in 1 out 5 patients with tumour nodules $<2$ cm in diameter.

### Chemotherapy in cervical carcinoma

S. Powell

*For the London Gynaecological Oncology Group, UK.*

The poor survival of FIGO stage IIb, III, IV and early stage node positive cervix cancer has led to interest in chemotherapy in addition to standard methods of local treatment. We studied four chemotherapy regimes in patients with recurrent cervix cancer.

One hundred and twenty-five patients received treatment: (1) 41 received single agent ifosfamide (1.5 g m$^{-2}$, days 1–5); (2) 39 received ifosfamide (as (1)) and cisplatinum (50 mg m$^{-2}$, day 1); (3) 28 received cisplatinum (20 mg m$^{-2}$, days 1–3), etoposide (120 mg m$^{-2}$, days 1–3) and bleomycin (15 mg, day 1); (4) 11 received cisplatinum (as (3)), methotrexate (100 mg, day 1) and bleomycin (15 mg, day 1); (5) 6 others received cisplatinum alone or in other combination. Treatment schedules were repeated 3 weekly.

Three patients in (1) and six patients (3) had adenocarcinoma. None of these responded and were excluded. Response rates were: (1) 33% (12/36); (2) 38% (12/31); (3) 40% (8/20); (4) 30% (3/10) and (5) 60% (3/5). Comparative haematological, renal and neural toxicity will be presented.

When chemotherapy was given neo-adjuvantly, response rates were higher: treatment (3) 67% (6/9) and treatment (4) 57% (4/7). Active agents have been found for squamous carcinoma of the uterine cervix, but combination chemotherapy has not led to significantly enhanced efficacy.

### Pelvic field irradiation: An investigation of accuracy of treatment delivery

S.E. Griffiths & G.G. Khoury

*Regional Radiotherapy Centre and University Department of Radiotherapy, Cookridge Hospital, Leeds, UK.*

In previous studies 16% of supine and 26% of prone pelvic treatments using skin marks showed a lateral shift of 10 mm or more.

Fifteen patients receiving pelvic irradiation were studied to see if reproducibility could be improved by using lateral alignment lasers. Films were taken at the initial treatment simulation and on 4 subsequent occasions. Patients were set up initially without and then with the use of lateral lasers and films taken for comparisons.

Also, using a body phantom, we have measured shifts occurring with differing degrees of lateral rotation.

In comparison with our initial study we find that the magnitude and frequency of lateral shift errors is less, and that this is further reduced by the use of lateral lasers.

Clinically significant caudo-cephalic shifts of up to 2 cm are seen and we are currently studying means of reducing these errors.

Our experience will be presented and discussed together with recommendations for optimising treatment delivery.

**The use of transvaginal and transrectal endosonography to assess vaginal tumour**

J.E. Browning & E.C. Whipp

*Radiotherapy Centre, Bristol, UK.*

Current methods of assessment of vaginal tumour include examination under anaesthetic and computerised tomography. Magnetic resonance imaging is being evaluated.

In this study, 8 patients with vaginal tumour were endosonographically assessed. Three had primary vaginal carcinoma and secondary tumour arose from the cervix (2), colon (2) and bladder (1).

The patients were unanaesthetised and had not micturated for 1 h. A Bruel and Kjaer type 1846 scanner was used. Transvaginal sector scanning was performed in the supine position with the 7 MHz 8537 probe. The site, depth and extent of tumour could be demonstrated and measured. For gynaecological tumours a FIGO or T(NM) staging was derived. A transrectal radial probe (type 1850 with a 5.5 MHz 90 degree transducer) was introduced with the patient in the left lateral position. It demonstrated the integrity or otherwise of the rectal wall.

All patients were treated by radiotherapy.

Endosonography is a simple, convenient and well tolerated procedure. It gives accurate information on local tumour extent upon which treatment modality and dosimetry may be based.

Tumour regression after treatment may be monitored by this method.

### Paediatric and Lung Cancer and Palliation

**Clinico-pathological review of cases registered as primary malignant tumours of the liver in children in the West Midlands between 1957–1986: A West Midlands Regional Children's Tumour Registry (WMRCTR) study**

S.E. Parkes, A.H.C. Cameron, K.R. Muir, J.R. Mann, F. Raafat, J.R. Pincott, N. Kasthuri & L.C. Ingram

*Birmingham Children's Hospital, Birmingham, UK.*

Fifty-six children identified by the Birmingham and West Midlands Regional Cancer Registry as having primary malignant tumours in the 30 years 1957–1986 in the West Midlands RHA area (pop. 1.2 M children), were reviewed during data collection for the WMRCTR. Eight cases were excluded owing to age (3) and residence (5).

After pathological review, 4 groups of tumours arising in the liver were identified: Hepatoblastoma (25), hepatocellular carcinoma (2) [='classical liver tumours']; rhabdomyosarcoma (10); yolk sac tumour (3). Cases of metastatic neuroblastoma (2), non-Hodgkin's lymphoma (1), mesenchymal hamartoma (1), cirrhosis (1) and neonatal hepatitis (1) were excluded. Two cases are still under review.

Commonest symptoms in the 27 classical liver tumours were large liver, swollen abdomen, fever, anorexia and vomiting. Fifteen presented in the right lobe, 1 midline, 1 in the left, 8 bilateral and 2 unspecified. Distant metastases were present in 7 cases. Surgical procedures consisted of resection (14), biopsy (10) and none (3). Fifteen patients had chemotherapy, 6 radiotherapy and 6 both. One of the hepatoblastoma patients died at birth. Twenty-six percent of these children had a congenital abnormality or other recognised predisposing factor: Beckwith's syndrome (1), hemihypertrophy (2), polyposis coli (1), tyrosinosis (1), renal dysplasia (1) and undescended testis (1).

Main symptoms in the remaining 13 children were large liver, anorexia and abdominal pain. Three tumours arose in the right lobe, 2 in the left, 4 were bilateral and 4 unspecified. Distant metastases were present in 9 cases. Four of this group underwent resection, 9 having biopsy alone. Five had chemotherapy, 6 radiotherapy and 2 both. There were no associated malformations or syndromes in this group although the maternal grandmother of one child with rhabdomyosarcoma had breast cancer, which could be indicative of Li-Frau meni syndrome. Long term survival was seen only in hepatoblastoma, with 6 (25%) of the patients still alive >5 years after diagnosis (range 5 to 21 years).

**Workload patterns at the Children's Hospital, Birmingham (BCH) – Oncology a regional speciality**

R.J. Wilde[1], K.R. Muir[2] & J.R. Mann[3]

[1]*Department of Community Medicine, Central Birmingham Health Authority;* [2]*West Midlands Regional Children's Tumour Registry, Birmingham Children's Hospital; and* [3]*Department of Oncology, Birmingham Children's Hospital, Birmingham, UK.*

BCH has been stretched beyond its limits, in terms of accommodation, manpower and funding. The growth in designated regional specialties (DRS), of which there are 17, is probably largely responsible for this. These use 50% of the hospital beds and provide specialist care for children in the West Midlands Health Authority Region. As a case in point, we have examined oncology to investigate a workload increase of 20% in 5 years observed through hospital activity analysis (HAA). Oncology is the largest DRS (26% of DRS bed usage in 1986) thus warranting further study.

Information collected by HAA was supplemented by data collected by the West Midlands Regional Children's Tumour Registry. Variables studied to assess changes in workload with time included childhood population, cancer incidence, referral patterns, survival rates and the effect of shared care.

The results showed that both childhood population (1.01 million in 1986) and childhood cancer incidence (146 cases per million per year during 1975–1985) have remained relatively constant in recent years. However, increases were found in the referral rate to BCH (from 49% of cases in 1975, to 71% of cases in 1985) and in survival rates (e.g. 3-year survival for leukaemia increased from 53% in 1973, to 65% in 1983). Shared care was also found to have increased (from 4% in 1980, to 27% in 1986).

In conclusion, the increased workload of paediatric oncology at BCH may most readily be explained by increases seen in referral and survival rates partially counteracted by a simultaneous increase in shared care. Such explanations suggest a successful service.

**A phase II study of actinomycin-D in small cell lung cancer**

E. Cornford & D.A.L. Morgan

*Hogarth Centre of Radiotherapy and Oncology, General Hospital, Nottingham, UK.*

Actinomycin-D has a wide spectrum of activity against solid tumours, but there is almost no published data on its activity against small cell lung cancer (SCLC), one of the most common of chemosensitive malignancies. The drug is relatively inexpensive, a consideration of increasing importance at the present time. A study was therefore conducted to assess its activity in patients with SCLC.

Fourteen patients with previously-untreated, measurable, extensive-stage SCLC received actinomycin-D, $50\,mcg\,kg^{-1}$ i.v., repeated at 21 day intervals. Treatment was discontinued, and more conventional therapy instituted at any signs of tumour progression, or if there were no objective signs of response, using UICC guidelines, at the time that the third cycle was due.

No objective responses were seen; gastrointestinal toxicity was moderate (grades II–III) in most cases; no significant haematological toxicity was seen.

Actinomycin-D, as administered here, is lacking in activity in SCLC.

### A numerical discriminant applied to prediction of survival in inoperable carcinoma of the bronchus

J. Thorogood, T.M. Collins, D.V. Ash & H.J. Close

*Cookridge Hospital, Leeds, UK.*

An evaluation of the palliative role of radiotherapy in inoperable lung cancer revealed that 16 out of 96 patients studied (17%) were dead within 3 months of treatment and were unlikely to have benefited from therapy. In order to try and identify these patients prospectively a statistical discriminant approach was used to formulate a suitable index to predict survival to 3 months. This index comprised weight loss in the previous 6 months, performance status, extent of disease and blood lymphocyte count.

A simple tree diagram was developed for clinicians to follow-through for a given patient, to arrive at one of 16 discrete probabilities of that patient being alive 3 months later. No calculations are required for its use and it is based on the index, using 4 factors only. The survival probabilities ranged from over 0.95 for 3 or 4 factors in the good prognosis category, to <0.20 with 3 or 4 factors in the poor category.

When applied to a prospectively collected set of 80 patients, the index achieved an accuracy of 97% in predicting those who would survive for longer than 3 months. It achieved a low accuracy of 26% in those dying before 3 months because these cases presented with 2 good factors and 2 poor.

### Palliative radiotherapy for bone metastases: Why don't British radiotherapists give single fractions?

E.J. Maher, A. Marks & A. Crellin

*Regional Centre for Radiotherapy and Oncology, Mount Vernon Hospital, Northwood, Middlesex, UK.*

Forty-two radiotherapists were asked about treatment of a theoretical patient with thoracic spine metastases from breast cancer. Fifteen of 42 would give 1 fraction, 19, 2–5, and 8, 6–10. Fifty-three percent of those from the North and Midlands gave single fractions, compared with 26% of those from London and the South. Reasons for fractionation included: Training (63%), departmental policy (44%), acute side-effects (37%), fears about recurrence and retreatment (37% each). Reduction of late damage (19%), perceived improved healing (11%), and better tumour regression (26%) were less influential. Single fractions might be used if there was machine-pressure (82%), patient lived a long way away (70%), or was 70+ (74%), the primary was lung not breast (63%), or metastatic site was outside the vertical column (100%). Non-fractionators' reasons included training (67%), with departmental policy less important (13%). Most would fractionate in the presence of neurological symptoms (73%), but young age (13%), bone destruction (20%) and first metastatic site (33%) were less persuasive.

Despite published evidence that single doses of RT provide safe and effective palliation, this study confirms the preference for multiple fractions and highlights confusion about the scientific basis for this.

### A pain survey in an out-patient cancer clinic

E. Cornford & D.A.L. Morgan

*Hogarth Centre of Radiotherapy and Oncology, General Hospital, Nottingham, UK.*

That pain is a common symptom in cancer patients is well known. A survey was conducted to assess the extent of the problem in one radiotherapy and oncology practice.

Two hundred and seven consecutive out-patients with cancer (both new referrals and follow-ups) were asked if they were in pain at the time of clinic attendance, and if so, marked an assessment of its severity on a 10 cm visual analogue scale (VAS). Patient and tumour details and analgesic consumption were documented. The attending physician recorded whether the pain was likely to be due to cancer.

The data so obtained are summarised in the Table:

| *Tumour site* | *No. of patients* | *Mean age* | *No. in pain (%)* | *Mean VAS (cm)* | *Tumour pain* | *Taking analgesics* |
|---|---|---|---|---|---|---|
| Lung | 73 | 65 | 25 (34) | 4.2 | 22 | 19 |
| Breast | 58 | 60 | 23 (40) | 4.3 | 17 | 15 |
| Other | 76 | 61 | 25 (33) | 3.8 | 14 | 17 |
| Total | 207 | 62 | 73 (35) | 4.1 | 53 | 51 |

### Hemibody irradiation (HBI) for metastatic bone pain

P.J. Hoskin, C.L. Harmer & H.T. Ford

*Royal Marsden Hospital, Sutton, Surrey, UK.*

Thirty-eight patients treated between January 1979 and December 1987 with HBI for multiple sites of bone pain from metastatic disease have been reviewed. Forty-six individual treatments were given, 15 upper body (UHBI) and 31 lower body (LHBI). Administered doses were UHBI; 6 Gy mpd (14 patients), 7 Gy mpd (1 patient), and LHBI; 8 Gy mpd (29 patients), 6 Gy mpd (1 patient), 4 Gy mpd (1 patient), delivered in a single fraction at a dose rate of 0.15–$0.2\,Gy\,min^{-1}$. Twenty-three patients had primary prostatic carcinoma (PC), 13 had myeloma (MM), 1 had an osteosarcoma and 1 metastatic melanoma. Response has been assessed retrospectively and defined as an improvement in pain with stable or reducing analgesic requirements. Overall response rate was 83%; 25/29 (86%) of treatments for PC and 12/15 (75%) of treatments for MM. Three complete responses were seen (i.e. complete resolution of pain and no analgesic requirements); 2 with PC and 1 with MM. Fourteen of 15 (93%) of UHBI responded and 23/70 (77%) of LHBI responded. Mean overall survival from HBI was 8.9 months (range 1–36); in patients with MM mean survival was 10.2 months (range 1–36) and in PC was 5.3 months (range 1–19). Six patients with MM are currently alive (mean survival from HBI 12.2 months) and 1 with PC is alive 3 months from HBI. Significant toxicity occurred in 26 patients (68%) with one fatality from pneumonitis following UHBI. Six patients subsequently had further local radiotherapy within the previously treated hemibody area without complications.

### Haematological Neoplasia and Sarcomas

### Severe lung toxicity following weekly low-dose chemotherapy regimen in patients with non-Hodgkin's lymphoma

M. Quigley, M. Brada & A. Horwich

*The Royal Marsden Hospital and Institute of Cancer Research, Sutton, Surrey, UK.*

In 1985 and 1986 we carried out a phase II study of a new chemotherapy regimen alternating low-dose cyclosphamide and mitoxantrone with bleomycin and vincristine and continuous oral prednisolone. The regimen was designed for poor prognostic category patients with non-Hodgkin's lymphoma (NHL), who were either elderly or had recurrent intermediate or high grade NHL.

Nineteen patients were treated and 6 developed unexpected lung toxicity 8–10 weeks after start of chemotherapy. The pneumonitis was fatal in 5 patients. This occurred at bleomycin doses $<50\,\mathrm{U\,m^{-2}}$ and cyclophosphamide $<1{,}875\,\mathrm{mg\,m^{-2}}$.

Thirty-five percent incidence of apparent drug induced pneumonitis following low doses of bleomycin in evaluable patients suggests an increased susceptibility to drug induced pulmonary damage in patients with non-Hodgkin's lymphoma.

### A comparison of gallium SPECT and CT in mediastinal Hodgkin's disease

S. Karimjee, M. Brada, J. Husband & V.R. McCready

*Royal Marsden Hospital and Institute of Cancer Research, Sutton, Surrey, UK.*

The aim of this project was the study of the role of high dose gallium (Ga-67) scanning in the assessment of mediastinal Hodgkin's disease (HD).

Comparison of thoracic CT and Ga-67 single photon emission computed tomography (SPECT) images was undertaken using high dose Ga-67 in patients with biopsy proven HD at presentation and after treatment.

Sixteen patients with HD had pre-treatment CT scan and Ga-67 SPECT studies. In 15 patients all sites of disease demonstrable on CT were also shown on SPECT scan; in one patient only one of three sites of disease shown on CT scan was Ga-67 positive. In three patients four sites demonstrated on SPECT were not seen on CT scan. Overall of 24 Ga-67 positive mediastinal sites, 4 were not detected on CT. A number of patients underwent post-treatment assessment as part of a prospective study.

The apparent 16% false negative rate for CT compared to SPECT suggests an additional role for high-dose Ga-67 imaging in the initial staging of HD. Early post-treatment results also indicate that G-67 scanning may help in detecting residual mediastinal disease.

### Therapy of immune-mediated thrombocytopenia post autologous bone marrow transplantation – A pilot study

F.J. Giles[1], A.C. Newland[2] & A.H. Goldstone[2]

*Departments of Haematology, [1]University College/Middlesex Hospital and [2]The London Hospital, London, UK.*

Autologous bone marrow transplantation (ABMT) is an established procedure in the therapy of haematological malignant disease. A proportion (25%–40%) of patients display persistent thrombocytopenia after ABMT. An autoimmune basis for this delay in recovery of peripheral cell counts has been postulated and a high incidence of cell specific antibodies to platelets (52%) and neutrophils (65%) has been detected in the early post-graft platelet count recovery post allogenic bone marrow transplantation. Potential factors of relevance to the formation of autoantibodies include the patient's underlying malignancy, pregraft chemotherapy and/or radiotherapy, *in vitro* damage to stem cells during graft storage, the engraftment procedure, insufficient numbers of inadequately functioning T-lymphocyte suppressor cells in the early post-graft period and post-graft blood component transfusions. The use of recombinant GM-CSF to accelerate the rate of recovery of peripheral blood counts post ABMT may be associated with the development of platelet-associated immunoglobulins. Prolonged thrombocytopenia post ABMT increased the patient's risk from bleeding, either spontaneous or associated with sepsis, and increases the risk associated with prolonged blood component transfusion requirement. The administration of intravenous immunoglobulin is an established modality of therapy in idiopathic thrombocytopenic purpura. We discuss the format of a pilot study to ascertain whether intravenous immunoglobulin therapy can accelerate delayed platelet recovery post ABMT and some initial data from one such study.

### Lung toxicity of melphalan and steroid combination therapy in multiple myeloma

F.J. Giles[1], C.R.J. Singer[1], A.H. Goldstone[1], J.S. Tobias[2] & J.D.M. Richards[1]

*Departments of [1]Haematology and [2]Radiotherapy, University College Hospital, London, UK.*

As reported elsewhere at this meeting, we have found upper hemi-body irradiation (UHBI) to be an effective, well tolerated therapeutic modality in advanced multiple myeloma. This procedure involved the administration of a median dose of 7.5 Gy from a slow cobalt source to the upper body in 35 patients, 18 of whom also received lower HBI. In this series, the only procedure related death that occurred was attributed to radiation-induced pneumonitis. Three other patients were also diagnosed as having post therapy pneumonitis, 2 of whom required and responded to oral corticosteroid therapy. This gave a total incidence of overt symptomatic pneumonitis of 14% following UHBI in this series of heavily pre-treated multiple myeloma patients. In order to delineate the extent of therapy-induced pneumonitis, we have begun to do baseline lung function tests before the UHBI procedure followed by sequential tests in the post therapy period. In 7 of the initial 10 (non-smoking) patients who had lung function tests performed prior to their UHBI obstructive ventilatory defects were demonstrated, 1 patient having a combined obstructive and restrictive ventilatory defect showing 2.3 l loss of lung volume and 0.35 l air trapping. The other 6 patients had air trapping ranging from 0.4 to 1.45 l. The median number of courses of melphalan/prednisolone therapy received by these patients was eight. All had suffered occasional lower respiratory tract infections during this therapy. No other recognised risk factors for lung damage were elicited on detailed history taking in this patient group. These initial data have led us to initiate a systematic programme of lung function assessment in our myeloma patient population.

**Alpha interferon therapy of essential thrombocythaemia**

F.J. Giles & A.H. Goldstone

*Department of Haematology, University College and Middlesex Hospitals, London, UK.*

The efficacy of alpha interferon in the control of the elevated platelet count which is a feature of a sub-group of patients with CGL has been noted. We have extended our use of interferon from this indication and assessed its efficacy in essential thrombocythaemia (ET) both in a pilot study and in an ongoing collaborative clinical study. We have now achieved a platelet count of $600 \times 10^9\ l^{-1}$ in 11 of 12 sequentially referred patients with ET. The remaining patient for personal reasons will not permit the administration of an adequate amount of interferon therapy. A normal platelet count was achieved in the relevant patients in all cases within 8 weeks of commencement of therapy at a dosage of 3 Mu daily s.c. with a dosage increment to 5 Mu daily if required after 2 or 4 weeks therapy. On this pilot study dosage reduction, because of adverse side effects, has not been required during the phase of induction of a normal platelet count. We have begun a clinical trial to assess the long term efficacy of alpha interferon in ET.

**Prospective study of infections in multiple myeloma and related disorders**

R.M. Hargreaves & J.R. Lea

*John Radcliffe Hospital, Oxford, UK.*

A group of 76 patients with multiple myeloma, 13 patients with Waldenstrom's macroglobulinaemia and 15 patients with monoclonal gammopathy of uncertain significance (MGUS) were followed prospectively for a period of up to twelve months to assess their rate of infections and to identify possible infection predictors. Infections were classified according to severity as trivial, moderate or major, the latter two groups representing potentially life threatening events. During the twelve month study period, there were 17 deaths of which 10 were directly attributable to infection. There were 20 episodes of major infection in 17 patients (13 pneumonia, 5 septicaemia, 2 pyrexia of unknown origin); 31 episodes of moderate infection in 25 patients (14 originating in the respiratory tract) and 54 episodes of trivial infection. There was a clear seasonal variation in moderate and trivial infections (the majority occurring in the period November–April) while major infections were evenly spread over the year. Most patients with myeloma were immunosuppressed (non-paraprotein immunoglobulin levels below the lower limit of normal) while only a minority with Waldenstrom's macroglobulinaemia and MGUS were. In the group of patients with multiple myeloma, there was no clear relationship between presence or degree of immunosuppression and susceptibility to infection. Other features investigated included smoking habits, paraprotein class, disease stage at diagnosis and chemo/radiotherapy at the time of infection, but no significant correlations with infections were found.

**Pre-operative chemotherapy in malignant fibrous histiocytoma of bone (MFHB)**

H.M. Earl, L. Morittu, D. Miles, J. Pringle, H. Kemp & R.L. Souhami

*London Supra-Regional Bone Tumour Service, University College Hospital, London, WC1 6AU and Royal National Orthopaedic Hospital, Stanmore, Middlesex, UK.*

Malignant fibrous histiocytoma of bone (MFHB) is usually treated by surgery alone, and published series show a five year survival rate of only 30% (Capanna *et al.*, *Cancer*, **54,** 177 (1984)). Recent reports have indicated that this rare tumour may be responsive to chemotherapy. Since 1985 all cases of MFHB presenting to the London Supra-Regional Bone Tumour Service have been treated on a protocol of pre- and post-operative chemotherapy. Patients were aged 33–69 years, 2M and 4F, and tumour sites were distal femur (4), distal tibia (1), and superior pubic ramus (1). Patients were treated with methotrexate 4–8 $g m^{-2}$ on day 1, and ifosfamide 3 $g m^{-2}$, with doxorubicin 60 $mg m^{-2}$ on day 10 of a 28 day cycle. Two courses were given prior to surgery, and 2 courses post-operatively. All 4 patients with distal femoral lesions had successful endoprosthetic replacement, one had below knee amputation, and one had complete resection of tumour of the superior pubic ramus. Histopathological analysis revealed that 2 patients had complete tumour necrosis, 2 had >95% tumour necrosis, and only 2 patients had little evidence of response to chemotherapy. All patients are alive and disease-free 2–30 months after surgery. One patient developed CT scan evidence of pulmonary metastases 6 months after surgery, and achieved a complete response to ifosfamide and doxorubicin. This report demonstrates that MFHB is a chemosensitive bone tumour. Patients with this disease should be included in defined protocols to determine if long term survival is improved by adjuvant chemotherapy.

**Feasibility of retreatment of previously irradiated soft tissue sarcomas of the limb and limb girdle**

J.D. Graham, M.H. Robinson & C.L. Harmer
*Department of Radiotherapy, Royal Marsden Hospital, London, UK.*

Ten patients with soft tissue sarcomas of the limb or limb girdle have been re-irradiated on 12 occasions for local recurrence following previous limb conserving treatment. The histological types were: Liposarcoma (5), fibrosarcoma (2), malignant fibrous histiocytoma (1), rhabdomyosarcoma (1), synovial sarcoma (1) with 6 tumours being of high grade malignancy. Retreatment was given with palliative intent in 5/12, and with curative intent following marginal excision in 7/12, at a median time of 15 months after initial treatment (range 8–324 months). Initial radiotherapy was given at doses ranging from 1,362–1,941 ret (median 1,650 ret, dose range 33–60 Gy) whilst for retreatment the curative group received a median 1,768 ret (dose 33–60 Gy) and the palliative group a median 1,303 ret (dose range 12–50 Gy). Initial local control was achieved in 7/10 of patients and eventually following 8/12 courses (median duration of local control 9 months); one patient being controlled by further surgery and radiotherapy. Three patients died from metastatic disease with local control at the primary site. Early toxicity was not severe with most desquamation in 2/12. Late toxicity was severe in 1 case following the third treatment with late radionecrosis requiring amputation. Moderate oedema and diminished limb function were seen in 2 cases. Re-irradiation of the limb and limb girdle appears to be well tolerated and offers an alternative to amputation for local relapse following previous radical multimodality therapy.

**The role of radiotherapy in the management of intracranial meningiomas: The Royal Marsden Hospital experience**

J. Glaholm & H.J.G. Bloom

*Department of Radiotherapy and Oncology, Royal Marsden Hospital, Surrey, UK.*

One hundred and sixty patients with intracranial meningiomas were treated by one consultant at the Royal Marsden Hospital between 1958 and 1983 with megavoltage photon irradiation. Uniform dosages of 50 to 55 Gy were delivered to a target volume comprising the known volume of disease including a macroscopically clear surrounding margin. Photon energies from 2 Mv to 8 Mv were employed.

Survival parameters were measured from the time of diagnosis. Overall 10 year actuarial survival was 50% in all cases with an overall disease free survival of 60%. Considering only death from meningioma, the corresponding cause specific actuarial survival was 65%.

Prognosis was related to the extent of initial surgical resection. Of those who underwent subtotal tumour resection with subsequent minimal residual disease, the 10 year actuarial survival was 70%. Where only partial resection was possible the corresponding survival was 60% and in inoperable patients treated by radiotherapy alone 45%. Eighteen patients underwent complete resection of disease prior to irradiation with a resultant 10 year survival of only 20% owing to the high incidence of adverse histological subtypes in this group which included malignant meningioma, all of whom died within 5 years of diagnosis. These differences are statistically significant.

Performance status at the time of referral for post-operative radiotherapy was measured retrospectively using the Karnovsky scale. Patients with a performance rating of 80–100 had a 70% 10 year survival, whereas those with a score of less than 80 had a 40% survival. The difference is statistically significant.

Patients undergoing complete surgical resection and not requiring adjuvant irradiation have an inherently good prognosis. It is therefore not possible to select an appropriate control group; however, the long term survival of irradiated patients in this series supports the benefit of postoperative radiotherapy in patients with residual disease.

### Clinical and *in vitro* evaluation of JM8 (carboplatin) and JM9 (CHIP) in high grade glioma

C.J. Twelves[1], J.L. Darling[3], K.P. Healey[3], D. Miles[1], C. Ash[2], D.G.T. Thomas[3] & R.L. Souhami[2]

[1]*Guy's Hospital,* [2]*University College Hospital and* [3]*Institute of Neurology, London, UK.*

We have evaluated JM8 and JM9 in the treatment of high grade glioma, firstly in a phase II clinical study and secondly, by *in vitro* chemosensitivity assay.

Fifteen patients were treated (7 grade III and 8 grade IV) with JM8 or JM9. All had relapsed after surgery and either radiotherapy alone (3 pts) or radiotherapy and chemotherapy with procarbazine, CCNU and vincristine (12 pts). Median age was 45 yrs and ECOG score 2. Chemotherapy was JM8 400 mg m$^{-2}$ (10 pts) or JM9 300 mg m$^{-2}$ (5 pts) given every 4 weeks; response was assessed by CT scan. The response rate was 2/15 (1 response to JM8 and 1 to JM9). Myelosuppression was considerable and 3 patients developed grade IV neurotoxicity.

Chemosensitivity was assessed in 8 short-term cultures derived from biopsies in a separate group of patients with malignant glioma, by a 35 S-methionine uptake assay. The $ID_{50}$s ranged from 0.02 to 3.7 $\mu$g ml$^{-1}$ for JM8 and 0.008 to 2.8 $\mu$g ml$^{-1}$ for JM9. There was evidence of cross resistance between the two agents, but no evidence of cross resistance with CCNU. The range of sensitivities observed for JM8 and JM9 were comparable to those observed for cis-platinum in earlier studies.

JM8 and JM9 have activity against high grade glioma both clinically and *in vitro*, but toxicity was significant in this group of poor prognosis patients. Further evaluation of JM8 should be in good performance status patients.

### Conference Lecture

### The biological basis of radiation fractionation

J. Denekamp

*Gray Laboratory of the Cancer Research Campaign, Mount Vernon Hospital, Northwood, Middlesex, UK.*

The rationale for giving radiotherapy as a series of small doses instead of a single treatment has been developed long after it became common clinical practice. It is not at all clear whether the current 2.0–2.5 Gy per day for 4–7 weeks is optimized.

Recent experimental data have emphasized that differences in the response to repeated treatments exist in late reacting tissues compared with acutely responding tissues and tumours. It is therefore inappropriate to select new therapies based on therapeutic differences between skin and tumours. The relevant comparisons must be made with slowly proliferating tissues, e.g., lung or kidney.

The experimental data highlighting the differences in repair capacity and in regenerative capacity will be reviewed. The influence of reoxygenation and redistribution around the cell cycle between fractions will also be discussed.

These concepts have led to a new clinical regime – CHART – at Mount Vernon Hospital, involving 36 fractions given three times a day, with minimum interfraction intervals of 6 h and no break for the weekend. The biological basis of this trial and the criticality of the different elements will be presented.

### Bob Champion Cancer Trust Lecture

### Biological basis of drug sensitivity and resistance

A.L. Harris

*Department of Clinical Oncology, University of Oxford, UK.*

Although many tumours are curable with radiotherapy and/or chemotherapy, the majority of cancer patients die from their tumour with failure of local control or systemic recurrence. Tumours may be resistant *de novo* (primary resistance) or resistance may occur in recurrent tumour after initial response (secondary resistance). It is not known if the mechanisms are the same. The activity of the various treatments without excessive normal tissue toxicity implies that normal tissues are often more resistant than the tumours. It is not known which regulatory mechanisms control normal resistance and whether these mechanisms are important in tumour resistance or differential sensitivity of tumours. We have used two approaches to this problem:

1. *Isolation of mammalian cell mutants hypersensitive to anticancer drugs*

We have isolated a series of 19 different Chinese hamster ovary cell lines that are many fold more sensitive than normal for a range of cytotoxic agents. These include mitomycin-C, monofunctional and bifunctional alkylating agents, cisplatinum, radiation, bleomycin, m-AMSA, adriamycin, VP16, CCNU, vincristine, vinblastine, actinomycin-D. The CHO mutants were isolated by a replica plating method following EMS mutagenesis. They are being transfected with human genomic DNA to isolate human genes regulating the basal drug resistance mechanisms. Three mutants have been transfected back to wild-type and secondary transfections are in progress. A range of defects has been shown using DNA repair and mutational assays. Thus defects in single strand DNA break and double strand break repair, cross-link repair and spontaneous hyper and hypomutability has been shown. The mutation rate findings suggest both error prone and error correcting mechanisms are present in mammalian cells.

*2. Isolation of mammalian cell mutants resistant to anticancer drugs*

CHO cells were made resistant to chlorambucil by repeated incremental exposure. They are cross-resistant only to melphalan and nitrogen mustard and to a small extent ifosfamide. They were found to have a marked increase in a cytoplasmic protein which protects it against the toxicity to the alkylating agents. This was a particular glutathione transferase, known as Yc and is representative of a family of over 10 different types of glutathione transferases, so this is the first demonstration of a protective function for a specific transferase against a cross-linking alkylating agent. Approaches aimed at either inhibiting transferases or using transferase-activated drugs may greatly increase the therapeutic index of alkylating agents. Isolation of the genes regulating some of these pathways will enable investigation of the mechanism responsible for sensitivity and resistance and may provide new targets for therapy.

### Symposium: Biology of Tumour Vasculature and Metabolism

### The effect of vasoactive drugs on the fraction of hypoxic cells in experimental tumours and consequent alteration in the effectiveness of bioreductive radiosensitizers and chemotherapeutic agents

I. Stratford, J. Bremner, S. Cole, J. Godden & G.E. Adams

*MRC Radiobiology Unit, Chilton, Didcot, Oxfordshire, UK.*

It has been shown that administration of hydralazine to mice induces severe hypoxia in experimental tumours of various types. In principle, the induction of severe hypoxia in tumours by manipulation of blood flow can be exploited for enhancing the efficacies of some chemotherapeutic agents, including bioreductive radiosensitizers and alkylating drugs such as melphalan.

We have compared the efficacy of hydralazine and other established vasoactive agents including 5-hydroxytryptamine (5-HT), nifedipine and verapamil for their ability to increase the anti-tumour effectiveness of melphalan. Treating mice with hydralazine 15 min after melphalan results in an enhancement of $\sim$3.0 for melphalan induced delay in growth of either the RIF-1 or KHT tumours. Similar enhancements are achieved when hydralizine is given *before* melphalan. Administration of 5-HT also enhances tumour response but only when given *after* melphalan. Both hydralazine and 5-HT can induce close to 100% radiobiological hypoxia in the RIF-1 and KHT tumours. In contrast, nifedipine has no effect on tumour hypoxia fraction at a dose ($10\,\mathrm{mg\,kg^{-1}}$) which nonetheless substantially increases the anti-tumour effectiveness of melphalan. Hydralazine can also produce up to a 10-fold increase in the sensitizing efficiency of misonidazole and RSU 1069 in the KHT tumour in mice. This is likely to be due to the vasoactive agent inducing close to 100% tumour hypoxia after irradiation and so allowing greater expression of the differential toxicity of the sensitizers towards those hypoxic cells surviving the radiation treatment. Other radiosensitizers have been combined with hydralazine and it was found that the sensitizing efficiency of etanidazole and Ro 07–9963 was not significantly improved by subsequent administration of hydralazine. There was a small (2–4 fold) improvement in the sensitizing efficiency of pimonidazole. Only with RSU 1069 and misonidazole were substantial increases observed.

These results will be discussed in terms of the bioreductive cytotoxic effects of each of these compounds following treatment with hydralazine. Further, comparison will be made with the action of left-shifters of the oxyhaemoglobin association curve for their ability to induce severe tumour hypoxia.

### Molecular enzymology of bioreductive metabolism

P. Workman, M.I. Walton & K.L. Kooistra

*MRC Clinical Oncology and Radiotherapeutics Unit, MRC Centre, Cambridge, UK.*

Bioreductive activation of hypoxic cell-targetted cytotoxins is accelerated by the catalytic functions of a variety of enzymes. These are fairly well characterized for rodent liver and include microsomal enzymes cytochrome P-450 reductase and cytosolic xanthine oxidase and aldehyde oxidase. Much less is known about the enzymology of bioreductive activation in tumours.

We have investigated the bioreduction of the hypoxic sensitizers/cytotoxins benznidazole (2-nitroimidazole), CB 1954 (dinitrophenyl aziridine), SR 4233 (benzotriazene di-N-oxide) and E09 (aziridinyl indoloquinone). Tools used include specific inhibitors and substrates, inhibitory antibodies and purified enzymes. All reactions are inhibited or reversed by molecular oxygen, except reduction of CB 1954 by DT-diaphorase which predominates in the rat Walker tumour. Substrate and cofactor requirements differ. Involvement of each enzyme depends upon the drug and the particular stage of the multistep reduction sequence. For example, in mouse liver microsomes, P-450 reductase is predominantly involved in early events of benznidazole reduction while for SR 4233 the former enzyme has only a limited capability for direct reduction. For CB 1954, metabolism at the 4-nitro position predominates over the 2-position. Characterization of cytochrome P-450 isoenzyme specificities is underway.

We propose that rational selection of hypoxic cell cytotoxins could be based upon enzymological characterization of the reductases present in a biopsy specimen. This will be facilitated by development of (1) conventional and flow cytometric assays for specific reductases, and (2) techniques for non-invasive identification of hypoxic cells *in vivo*. Progress in these areas will be described.

### Vasoactive agents – Clinical potential?

N.P. Rowell

*MRC Radiobiology Unit, Harwell and Radiotherapy Unit, The Royal Marsden Hospital, Sutton, Surrey, UK.*

The potential for vasodilators to enhance bioreductive cytotoxicity in man depends not only on the development of bioreductives of low toxicity, but also on the ability of vasodilators to produce the necessary reduction in tumour blood flow without serious cardiovascular sequelae.

The physiological changes necessary to produce a fall in tumour blood flow are discussed with reference to a simple mathematical model incorporating the normal physiological responses to vasodilator therapy.

A study investigating the effects of single-dose hydralazine on tumour blood flow is discussed.